# A convergent fourfold hydrogen-bonding network controls a remote metal centre

**DOI:** 10.1039/d6sc05423d

**Published:** 2026-10-27

**Authors:** Heechan Kim, Kyunghwan Min, Juhwan Shin, Dongwhan Lee

**Affiliations:** a Department of Chemistry, Seoul National University 1 Gwanak-ro, Gwanak-gu Seoul 08826 Korea dongwhan@snu.ac.kr

## Abstract

Noncovalent interactions in the secondary coordination sphere play a critical role in regulating metal-centred reactivity, enabling metal centres with structurally similar primary coordination spheres to carry out vastly different chemistry. Despite the recognized importance of secondary-sphere hydrogen bonds (HBs), their quantitative energetic consequences for metal-centred redox chemistry remain poorly understood. To address this gap, we report a series of iron(ii) bis(tridentate) complexes featuring systematically varied intramolecular HBs in the secondary coordination sphere. In these complexes, *para*-functionalized pyridyl pendants installed around a common 2,6-bis(benzimidazolyl)pyridine core establish a converging network of intramolecular N–H⋯N contacts, the strength of which is finely regulated by the electron-donating ability of remotely located substituents. Despite sharing an essentially identical primary coordination sphere, these complexes exhibit dramatic variations in solution-state magnetic moments (*µ*_eff_ = 3.52–0.92*µ*_B_), Fe^II/III^ redox potential (*E*_1/2_ = +0.41 to −0.46 V *vs.* Fc/Fc^+^), and metal-to-ligand charge-transfer transitions (*λ*_max_ = 558–608 nm) as a function of HB strength. These findings highlight the functional role of what is often marginalized as the secondary environment, providing a quantitative framework for understanding how noncovalent interactions tune the chemistry of metalloenzymes and informing the design of bioinspired catalysts with tailored redox properties.

## Abstract 2

Four convergent secondary-sphere hydrogen bonds work collectively to control the spin state, optical transition, and electron-transfer thermodynamics of a remote metal centre.

## Graphical abstract

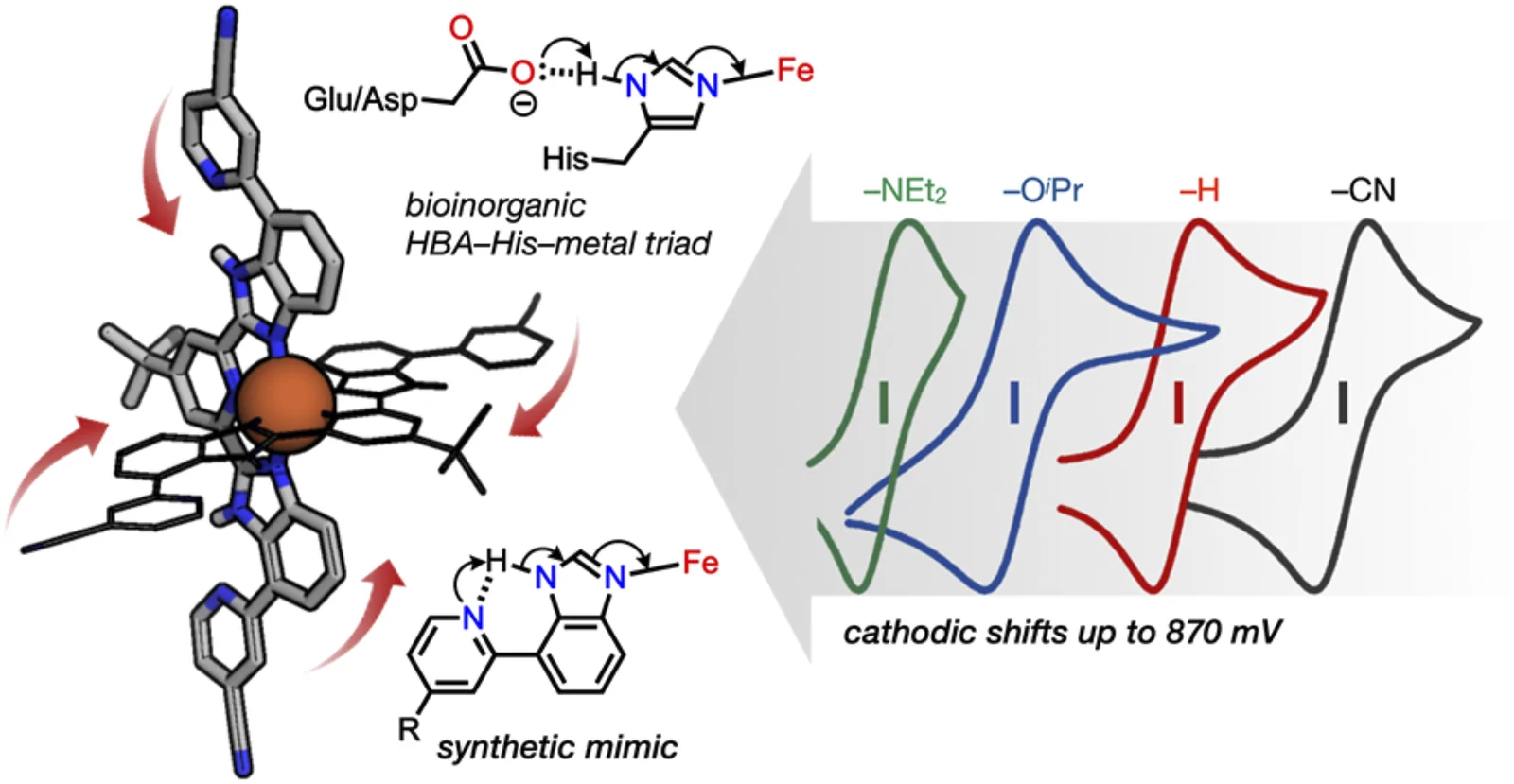

## Introduction

Metalloprotein function frequently depends on controlled changes in metal oxidation state, demanding that the protein environment precisely regulate metal-centred redox chemistry to achieve selective substrate activation and directional electron flow.^1–3^ Nature accomplishes this control across vastly diverse redox processes using only a limited repertoire of amino acid ligands, primarily imidazole (His), carboxylate (Asp/Glu), and thiolate (Cys).^4^ This apparent mismatch between chemical diversity and ligand economy highlights the functional importance of interactions beyond the primary coordination sphere. Accordingly, nature strategically positions functional groups in the secondary coordination sphere to impart specialized reactivity.^5,6^ In particular, secondary-sphere hydrogen bonds (HBs) positioned around the metal centre can organize substrates and intermediates while also tuning the donor properties of primary-sphere ligands and, consequently, the thermodynamics of electron transfer (ET).^7^

One of the most effective manifestations of this strategy is the “metal–histidine–hydrogen-bond acceptor (HBA)” triad (Fig. 1).^8–11^ In this motif, the noncoordinating N–H group of a metal-bound histidine side chain donates a hydrogen bond to a nearby acceptor, most often an anionic carboxylate. Such an interaction imparts partial imidazolate character to the histidine, thereby enhancing its electron-donating ability.^12,13^ In heme peroxidases, this enhanced donation underlies the proximal “push” mechanism (Fig. 1a): Asp247 accepts a hydrogen bond from the axial His170, increasing histidine-to-iron electron donation and assisting the formation of a high-valent iron centre.^14–18^ On the distal side, where substrate binding occurs, hydrogen-bond donors (HBDs; His42 and Arg38) instead “pull” on the terminal oxygen of a bound peroxide, facilitating heterolytic O–O cleavage. Together, these complementary interactions promote the formation and stabilization of the high-valent iron–oxo species.^9,10,13–18^

**Fig. 1:**
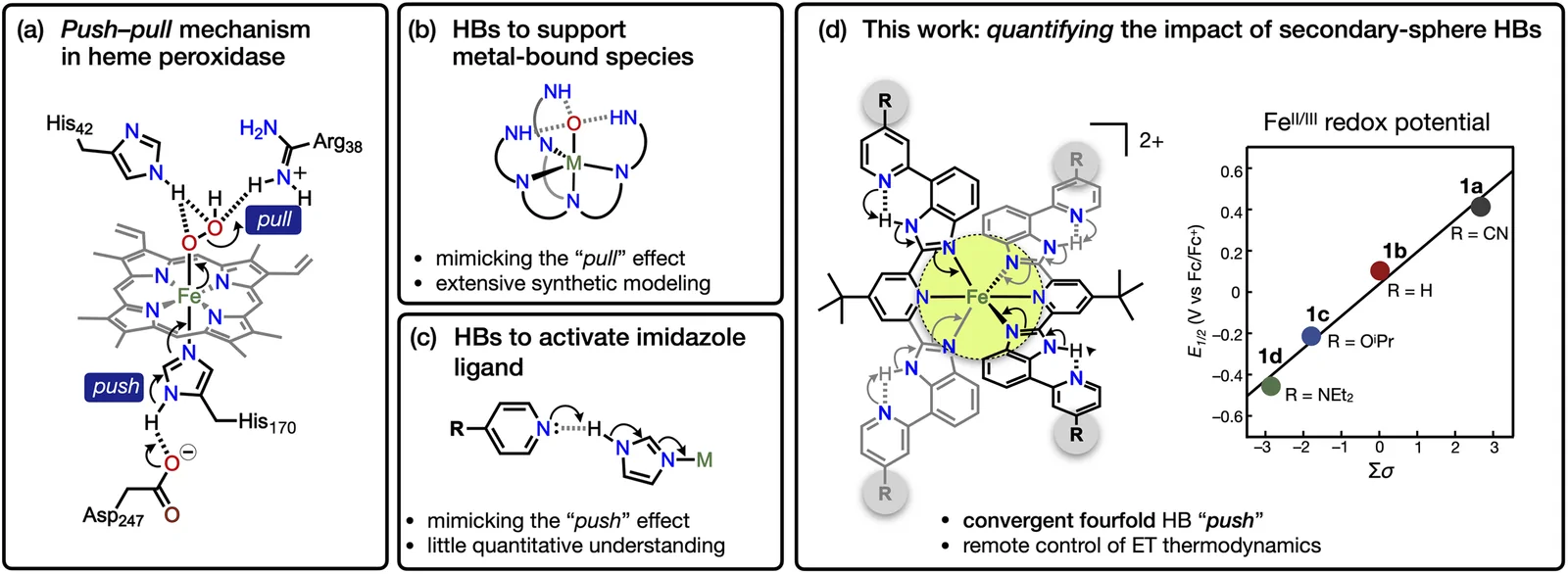
(a) Secondary-sphere HBs in heme peroxidases: a proximal “push” enhancing histidine-to-iron donation and a distal “pull” on the bound H_2_O_2_ substrate together facilitate access to high-valent Fe–oxo species. (b) Synthetic models for secondary-sphere HBs to metal-bound substrates. (c) Synthetic recapitulation of the “metal–histidine–HBA” triad. (d) A series of six-coordinate iron(ii) complexes with four imidazole donor units, enabling quantitative evaluation of secondary-sphere HB effects on Fe^II/III^ redox chemistry.

Efforts to reproduce secondary-sphere effects in synthetic mimics have largely focused on installing HBDs in the secondary sphere (Fig. 1b), where they stabilize metal–oxygen species and control their reactivity.^19–23^ These studies have established the kinetic and thermodynamic significance of secondary-sphere HBs to coordinated species.^24–34^ However, HBs acting on primary-sphere ligands, such as the noncoordinating N–H group of histidine (Fig. 1c), remain less quantitatively understood, particularly with respect to their ability to modulate ligand donor strength. This gap is especially striking because even subtle changes in ligand donor ability can perturb both redox and spin-state energetics, with profound consequences for metal-centred reactivity. These considerations raise a fundamental question: to what extent can secondary-sphere HBs control the redox potential, spin state, and electronic structure of a metal centre?

In this paper, we report a series of iron(ii) complexes (1a–d, Fig. 1d) designed to probe the impact of secondary-sphere HBs on metal spin state, electronic structure, and redox chemistry. These coordinatively saturated complexes feature a robust tridentate 2,6-bis(benzimidazolyl)pyridine scaffold with pendant pyridine groups positioned to form tight intramolecular HBs with the benzimidazole N–H HBDs. By varying the *para*-substituents on these pyridine units, the HB strength is systematically tuned while the primary coordination sphere remains essentially invariant. We postulated that a more electron-donating *para*-substituent would increase the basicity of the HB-accepting pyridyl nitrogen, thereby strengthening the intramolecular N_pyridyl_⋯H–N_imidazolyl_ contact. The more polarized N–H bond, in turn, would enhance the “imidazolate” character of the metal-bound benzimidazole N-donor, rendering the metal centre more electron rich. Such cascade effects converging at the metal centre should be manifested in a lower Fe^II/III^ oxidation potential, a stronger ligand field, and a red-shifted metal-to-ligand charge-transfer (MLCT) transition. Strikingly, secondary-sphere modulation shifts the Fe^II/III^ potential by up to 870 mV, corresponding to *ca.* 20 kcal mol^−1^, demonstrating that HB engineering can rival primary-sphere ligand modification in its impact on ET thermodynamics. These results provide a quantitative model for understanding how secondary-sphere interactions control metalloenzyme function and for designing bioinspired catalysts with tunable redox properties.

## Results and discussion

Despite the prevalence of metal–histidine–HBA triads in biology,^8–11^ analogous modules in synthetic systems remain scarce. A central challenge is that HBAs, typically Lewis-basic N- or O-donors, are themselves prone to coordinating the metal, making their precise placement in the secondary sphere challenging and often leading to undesired multinuclear byproducts. To overcome this problem, we devised benzimidazole–pyridine conjugates in which an HBA pyridyl unit is covalently linked to the benzimidazole core, geometrically preorganizing a tight intramolecular N–H⋯N contact.

Incorporation of two benzimidazole–pyridine conjugates onto a 2,6-bis(benzimidazolyl)pyridine scaffold afforded a series of structurally related ligands 5a–d, in which the HBA strength is systematically tuned by *para*-substituents of differing electronic demand (–CN, –H, –O^i^Pr, and –NEt_2_; Fig. 2). To distinguish HB-mediated noncovalent electronic effects from direct through-bond substituent effects, we also designed 5e, which has –NEt_2_ substituents on the terminal pyridyl rings as in 5d but is incapable of intramolecular HB formation.

**Fig. 2:**
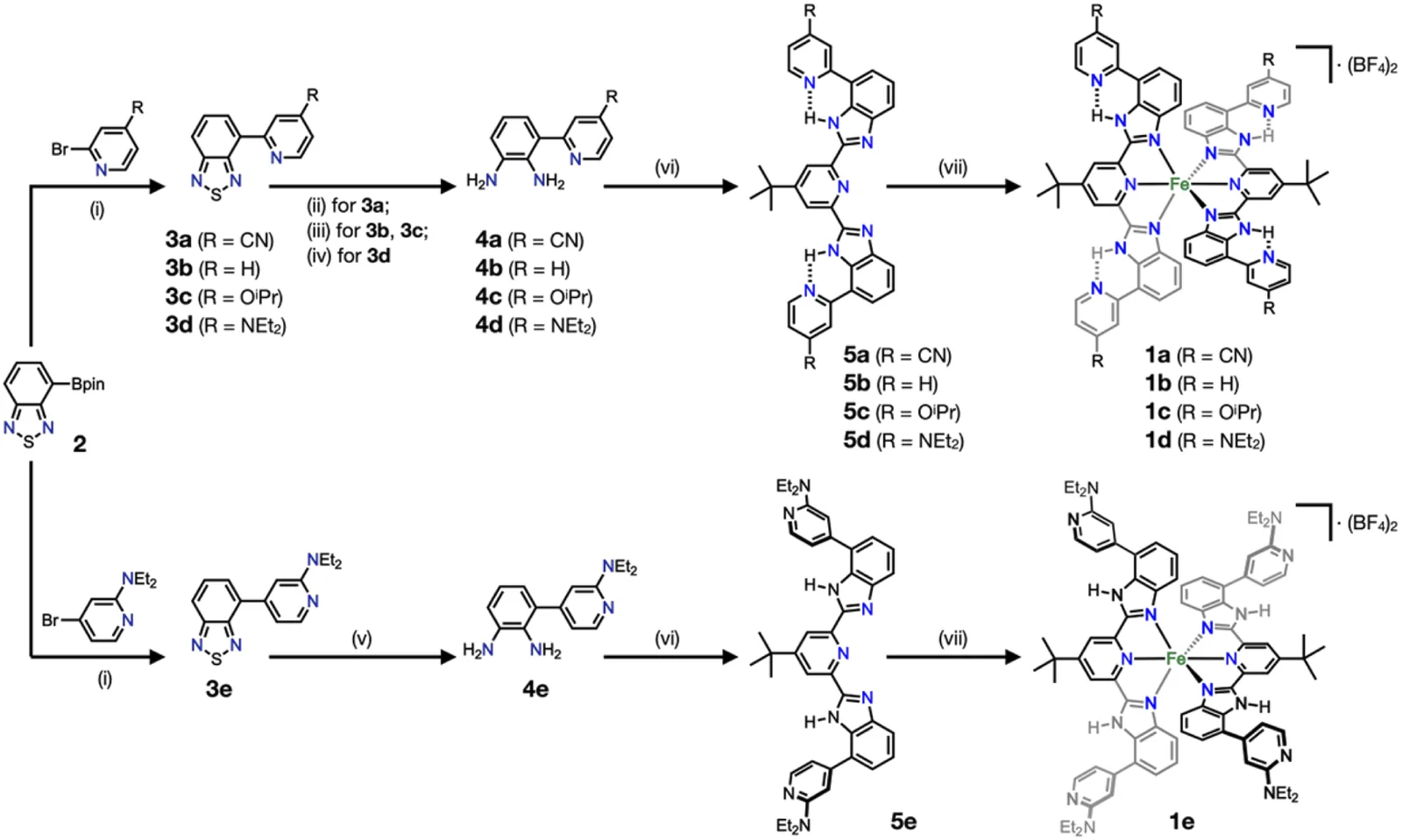
Synthetic routes to 1a–e. (i) Pd(PPh_3_)_4_, K_2_CO_3_, THF/H_2_O, Δ; (ii) Fe, HCl, EtOH, Δ; (iii) NaBH_4_, CoCl_2_·6H_2_O, EtOH, 0 °C to Δ; (iv) LiAlH_4_, THF, 0 °C to r.t.; (v) Fe, *p*-TsOH·H_2_O, EtOH, Δ; (vi) 4-(*tert*-butyl)pyridine-2,6-dicarbaldehyde, Na_2_S_2_O_5_, EtOH/H_2_O, Δ; (vii) [Fe(H_2_O)_6_][BF_4_]_2_, THF, r.t.

Suzuki–Miyaura coupling reactions of 2 with bromopyridine derivatives afforded 3a–e in high yields (81–99%). Sulfur-extrusion reactions of 3a–e provided the phenylenediamines 4a–e in moderate to high yields (53–88%), which were used in subsequent oxidative condensation reactions with *para*-substituted pyridine dialdehyde to construct the 2,6-bis(benzimidazolyl)pyridine scaffold of the desired ligands 5a–e (15–92%).

A single-crystal X-ray structure of 5d reveals a C-shaped conformation that maximizes intramolecular HBs, with N_py_⋯N_bzim_ interatomic distances of 2.727(1)–2.782(1) Å (Fig. S1). Solution NMR data are consistent with retention of this preorganized HB geometry. In CDCl_3_, the benzimidazole N–H resonances of 5a–d appear markedly downfield shifted and vary systematically across the series: *δ* 12.46 ppm for 5a; 12.74 ppm for 5b; 12.78 ppm for 5c; 12.86 ppm for 5d. These values correlate linearly with the Hammett substituent constants (*σ*_p_) of the pyridine substituents (Fig. S5), with more electron-donating groups producing more downfield N–H shifts, consistent with stronger HBA character and stronger intramolecular HBs.

IR spectroscopy provides an independent measure of the same trend: the benzimidazole N–H stretching frequencies decrease systematically from 3422 cm^−1^ for 5a to 3405, 3397, and 3327 cm^−1^ for 5b, 5c, and 5d, respectively (Fig. S6–S9). In contrast, 5e, which cannot form an intramolecular HBD⋯HBA contact, shows a sharp N–H stretch at 3637 cm^−1^ (Fig. S10), blue-shifted significantly (Δ*v* = 310 cm^−1^) relative to isomeric 5d. Together, the structural and spectroscopic data establish that the peripheral pyridyl substituents systematically tune the strength of the intramolecular HBs, a key design feature of our ligand system.

In THF, treatment of 5a–e with [Fe(H_2_O)_6_][BF_4_]_2_ afforded the corresponding iron(ii) complexes 1a–e as violet to dark-blue solids. The formulations of 1a–e were confirmed by high-resolution mass spectrometry (HRMS; Fig. S34–S38). Single-crystal X-ray diffraction studies established that both 1a and 1e adopt bis(chelate), *pseudo*-octahedral geometries (Fig. 3).^35^ Despite the presence of multiple N-donor groups, all ligands bind the metal in the intended tridentate fashion. This selective tridentate coordination contrasts sharply with a closely related ligand bearing 4-methyl substituents on the benzimidazole rings, which functions as a pentadentate donor to form a helicate instead (Fig. S16).^36^ With intraligand bite angles (∠N1–Fe–N3) of *ca.* 160° and trans interligand angles (∠N2–Fe–N2′) close to 180°, the Fe–N bond lengths (1.898(4)–1.993(2) Å; Table S3) indicate that the octahedral iron(ii) centres in both 1a and 1e adopt low-spin configurations in the solid state at 193 K and 100 K, respectively. Most importantly, 1a retains four intramolecular HBs between the benzimidazole N–H HBDs and the pendant pyridine HBAs upon metalation, with N4⋯N5 and N6⋯N7 distances ranging from 2.685(4) to 2.856(4) Å (Table S4). In contrast, X-ray diffraction analysis of 1e, an isomer of 1d, revealed the absence of such secondary-sphere HBs.

**Fig. 3:**
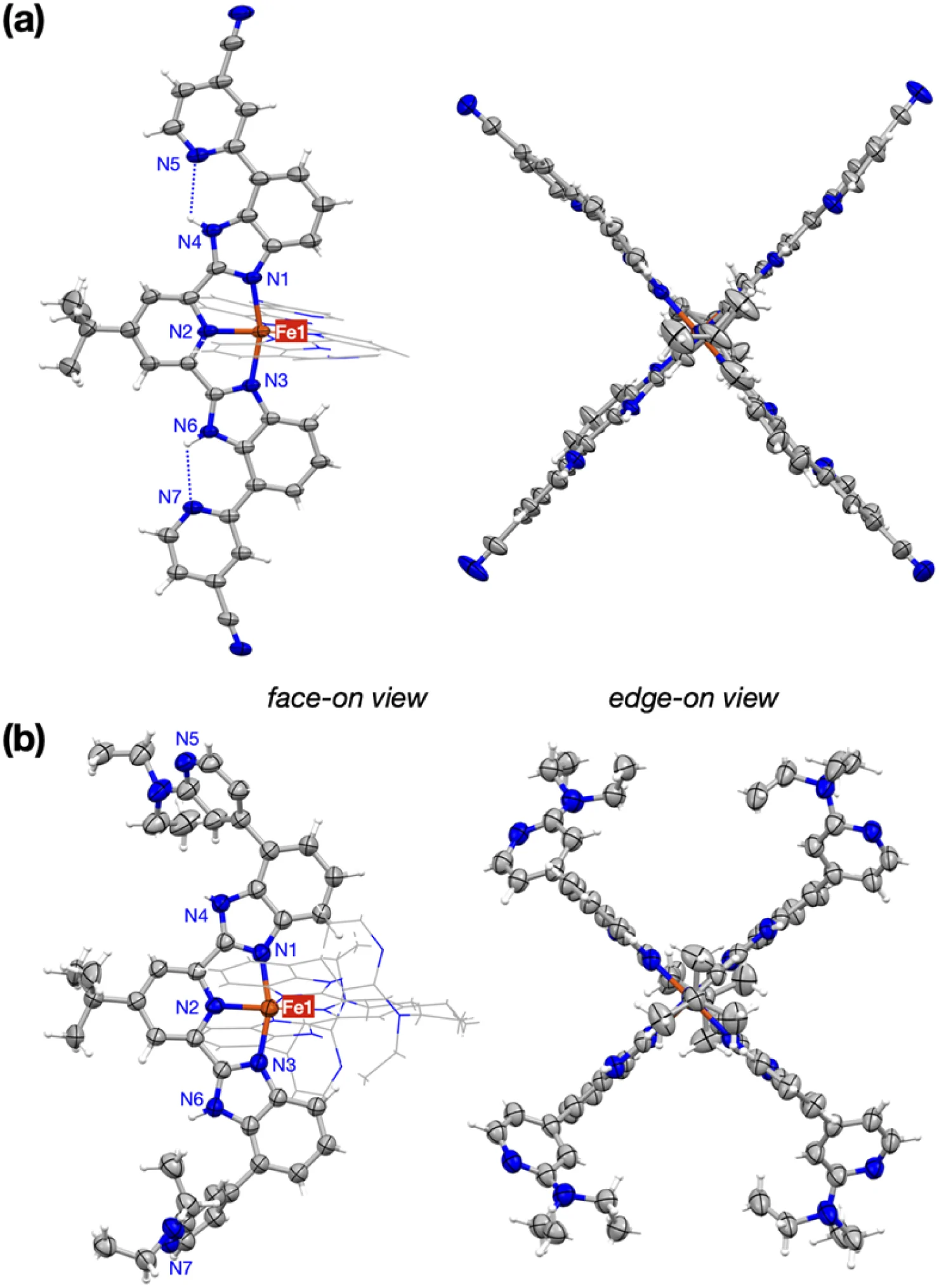
X-ray structures of (a) 1a and (b) 1e cations, shown with thermal ellipsoids at the 50% and 30% probability levels, respectively. For 1a, one of the four crystallographically independent molecules is shown; see Fig. S3 for the full asymmetric unit. In 1e, two of the four diethylamino groups on the pyridine rings are disordered over two positions; only one position is shown for clarity. Selected interatomic distances (Å) and angles (°) for 1a: Fe–N1, 1.982(2); Fe–N2, 1.900(3); Fe–N3, 1.980(3); N4⋯N5, 2.779(4); N6⋯N7, 2.685(4); N1–Fe–N3, 160.6(1). For 1e: Fe–N1, 1.960(3); Fe–N2, 1.898(3); Fe–N3, 1.961(3); N4⋯N5, 5.117(5); N6⋯N7, 5.084(5); N1–Fe–N3, 161.0(1). Solvent molecules and counteranions are omitted for clarity.

Having established that the designed HBs persist upon metalation, we proceeded to investigate how these secondary-sphere interactions affect the metal-centred electronic structure. The intramolecular HBs modulate the donor strength of the ligands, thereby altering the ligand-field strength at Fe(ii) and governing the spin states of 1a–e. Room-temperature effective magnetic moments (*µ*_eff_), determined by the Evans method in MeCN, reveal intermediate-spin behavior for 1a–c: *µ*_eff_ = 3.52*µ*_B_ for 1a; 3.39*µ*_B_ for 1b; 2.70*µ*_B_ for 1c (Fig. S31 and Table S5). Compared with the theoretical spin-only values for *S* = 2 (4.90*µ*_B_) and *S* = 1 (2.83*µ*_B_), such intermediate-spin behavior suggests a thermally populated spin-crossover equilibrium between low-spin and high-spin states in solution at room temperature. This interpretation is corroborated by the low-spin solid-state geometry of 1a determined by single-crystal X-ray diffraction at *T* = 193 K. Following the descending trend, 1d, which bears the strongest HBA, shows a markedly smaller *µ*_eff_ value of 0.92*µ*_B_, indicative of substantial low-spin character. In contrast, 1e, despite having the same NEt_2_ groups as 1d but positioned such that they cannot establish secondary-sphere HBs, deviates from this trend with *µ*_eff_ = 3.91*µ*_B_.

Electrochemical studies on 1a–d by cyclic voltammetry (CV) revealed reversible Fe^II/III^ couples with peak-to-peak separations (Δ*E*_p_) of 82–103 mV (Fig. 4a, top; see Fig. S24–S30 for full-range scans). A strong substituent dependence is observed across the series: 1a, bearing the most electron-withdrawing substituents (–CN), displays *E*_1/2_ = +0.41 V *vs.* Fc/Fc^+^, whereas 1b (–H) and 1c (–O^i^Pr) show cathodically shifted *E*_1/2_ values of +0.10 and −0.22 V, respectively. For 1d with the most electron-donating substituents (–NEt_2_), oxidation occurs at a remarkably lower potential, *E*_1/2_ = −0.46 V, despite the low-spin configuration of the metal. Because dialkylaminopyridine moieties typically undergo irreversible oxidation at substantially higher potentials (*ca.* +0.4 V *vs.* Fc/Fc^+^), the redox wave at −0.46 V is unambiguously assigned to the metal-centred Fe^II/III^ process.

**Fig. 4:**
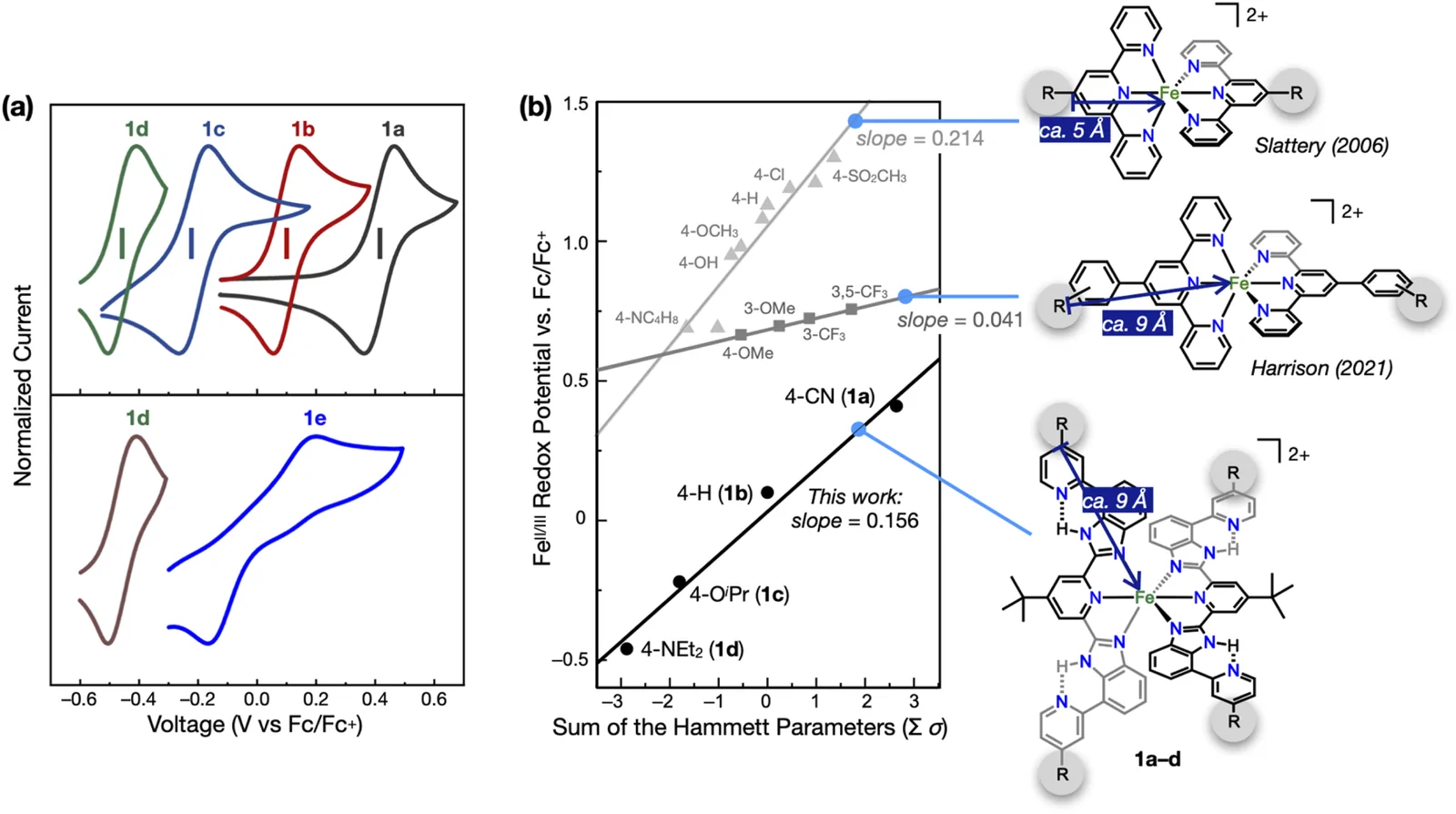
(a) Cyclic voltammograms (CVs) of 1a (black), 1b (red), 1c (deep blue), 1d (green), and 1e (blue). For each CV, the vertical bar denotes the half-wave potential (*E*_1/2_). (b) Fe^II/III^ redox potentials plotted against the summed Hammett substituent constants (Σ*σ*) for 1a–d and related bis(terpyridine) iron(ii) complexes.^37,38^

The Δ*E*_1/2_ = 0.87 V shift between 1a and 1d corresponds to an energy difference of *ca.* 20 kcal mol^−1^, underscoring the remarkable electronic effect that secondary-sphere HBs can exert on metal-centred redox chemistry. This “through-HB” substituent effect in 1a–d can be compared with two reference series of homoleptic bis(terpyridine) iron(ii) complexes, in which remote substituents modulate the Fe^II/III^ potential exclusively through covalent bonds (Fig. 4b).^37,38^ In the Slattery series,^37^ substitution at the 4-position of the central pyridine ring places the substituents in direct through-bond conjugation with the coordinating nitrogen, approximately 5 Å from the metal centre. This strong inductive and resonance coupling produces a significant dependence of *E*_1/2_ on Σ*σ* (slope = 0.21; light gray line in Fig. 4b), representative of primary-sphere functionalization. In the Harrison series,^38^ substitution at a much greater distance (*ca.* 9 Å), comparable to that in 1a–d, yields only a modest correlation (slope = 0.041; dark gray line in Fig. 4b), consistent with the attenuated through-bond influence of remote secondary-sphere functionalization. Against this reference frame, the Hammett slope of 0.16 observed for 1a–d is particularly informative: despite the comparable substituent distance, it is approximately four times larger than that of the Harrison series and approaches the value observed for primary-sphere substitution in the Slattery series. This comparison demonstrates that the intramolecular HB network effectively transmits the electronic influence of remote substituents to the metal centre, allowing secondary-sphere HB engineering to rival primary-sphere ligand substitution in its impact on redox thermodynamics.

The progressive increase in ligand electron-donating ability across the series is also reflected in the MLCT absorption bands (Fig. S32 and S33). Complex 1d, with the lowest Fe^II/III^ oxidation potential, displays the most red-shifted MLCT band (*λ*_max_ = 608 nm), followed by 1c (575 nm), 1b (561 nm), and 1a (558 nm). This behavior can be explained by the electron-donating *para*-substituents strengthening the intramolecular N⋯H–N hydrogen bond and thereby increasing the imidazolate-like character of the coordinating benzimidazole nitrogen. Because an imidazolate donor is substantially more electron-donating than a charge-neutral imidazole, this electronic modification raises the energy of the d-orbitals and increases the electron density at the iron centre. This mechanism anticipates, and is confirmed by, three correlated trends across the series 1a–d: (i) a cathodic shift in the Fe^II/III^ redox potential; (ii) a decrease in the solution-state magnetic moment toward the low-spin state; and (iii) a red-shift of the MLCT absorption band. The quantitative agreement of all three observables with the Hammett *σ*_p_ constants of the remote *para*-substituents underscores the fidelity of this through-hydrogen-bond electronic communication.

The effect of HBA positioning extends beyond redox thermodynamics: it also governs the reversibility of the ET process. In the CVs of 1d and 1e (Fig. 4a, bottom), 1d exhibits a reversible Fe^II/III^ couple, whereas 1e undergoes irreversible oxidation with an anodic peak potential (*E*_pa_) at *ca.* +0.20 V. Despite having the same –NEt_2_ substituents, 1e requires a much higher oxidation potential than 1d, which shows *E*_pa_ = −0.41 V. On the return sweep for 1e, a small cathodic feature appears at *E*_pc_ = +0.10 V, but the dominant cathodic peak occurs at *E*_pc_ = −0.17 V. We postulate that, upon oxidation, the pendant pyridine groups in 1e may act as internal bases and promote proton transfer from the benzimidazole N–H groups, thereby compromising the reversible Fe^II/III^ redox process. These comparative results underscore the importance of preorganizing HBD–HBA pairs to preserve the integrity of the local secondary-sphere environment during ET. Furthermore, the lack of intramolecular HBs in 1e is also evident from the MLCT transition (*λ*_max_ = 560 nm), which is blue-shifted by 48 nm relative to that of 1d (*λ*_max_ = 608 nm). This behavior is consistent with a larger charge-transfer energy gap associated with a less electron-rich iron centre, again indicating that, without the intramolecular HB network, the electron-donating effect of the –NEt_2_ groups is not effectively transmitted to the metal centre.

## Conclusions

Inspired by how metalloproteins create diverse functions from a limited set of biological ligands, we modeled the versatile “metal–histidine–HBA” triad to quantitatively elucidate how secondary-sphere HBs affect ligand-field strength, spin state, and redox chemistry at a metal centre. By incorporating multiple converging HBAs into well-defined, coordinatively saturated bis(tridentate) iron complexes, we systematically varied intramolecular HB strength while keeping the primary coordination sphere essentially unchanged. This design established a Hammett-type linear free-energy relationship between intramolecular HB strength and the Fe^II/III^ redox potential, with secondary-sphere substituent effects shifting the potential by as much as 870 mV (*ca.* 20 kcal mol^−1^, 1a*vs.*1d). These findings demonstrate that mechanism-based design of the secondary coordination sphere can rival primary-sphere ligand substitution in its impact on metal-centred properties. Efforts are currently underway in our laboratory to exploit the pyridyl–benzimidazole conjugate developed in this work for bioinspired redox catalysis.

## Author contributions

H. Kim: conceptualization, formal analysis, investigation, methodology, visualization, writing – original draft, writing – review & editing. K. Min: conceptualization, formal analysis, investigation, methodology, visualization, writing – original draft, writing – review & editing. J. Shin: conceptualization, formal analysis, investigation, methodology. D. Lee: conceptualization, funding acquisition, project administration, supervision, validation, writing – original draft, writing – review & editing.

## Conflicts of interest

There are no conflicts to declare.

## Supplementary Material

SC-OLF-D6SC05423D-s001

## Data Availability

CCDC 2547893–2547896 contain the supplementary crystallographic data for this paper.^39*a*–*d*^ The data supporting this article have been included as part of the supplementary information (SI). Supplementary information: experimental procedures, characterization data and SI figures, including ^1^H and ^13^C NMR spectra, crystallographic data, FT-IR spectra, HR-MS and elemental analysis. See DOI: https://doi.org/10.1039/d6sc05423d.
