# Supplementary material for: A convergent fourfold hydrogen-bonding network controls a remote metal centre
Source: Chem Sci. 2026 Oct 27. Online ahead of print. doi: 10.1039/d6sc05423d (PMC13730456; doi:10.1039/d6sc05423d)
Supplement: Supplementary file 1 [file SC-OLF-D6SC05423D-s001.pdf]

Supplementary Information (SI) for Chemical Science.
This journal is © The Royal Society of Chemistry

Electronic Supplementary Information

A convergent fourfold hydrogen-bonding network controls a remote metal centre

Heechan Kim[†], Kyunghwan Min[†], Juhwan Shin, and Dongwhan Lee[*]

Department of Chemistry, Seoul National University, 1 Gwanak-ro, Gwanak-gu, Seoul 08826, Korea

[†] These authors contributed equally to this work.
[*]e-mail: dongwhan@snu.ac.kr

Table of Contents

1. Experimental Section **2**

2. Synthetic Procedures **4**

3. Single-Crystal X-ray Diffraction Studies **16**

4. Supporting Tables **18**

5. Supporting Figures **21**

1. Experimental Section

General Considerations. All reagents were purchased from commercial suppliers and used as received unless otherwise noted. The compounds 4-(4,4,5,5-tetramethyl-1,3,2-dioxaborolan-2-yl)benzo[*c*][1,2,5]thiadiazole (**2**),[1] 2-bromo-*N*,*N*-diethylpyridin-4-amine,[2] and 4-(*tert*-butyl)pyridine-2,6-dicarbaldehyde[3] were prepared according to literature procedures or their slight modifications. Spectroscopic grade acetonitrile was used for UV–vis measurements. All air-sensitive manipulations were carried out under argon atmosphere in an MBraun LABSTAR Pro glovebox or by standard Schlenk-line techniques.

Physical Measurements. ^{1}H NMR and ^{13}C NMR spectra were recorded on a 500 MHz Varian/Oxford AS-500 spectrometer or a 400 MHz Agilent 400-MR DD2 Magnetic Resonance System. Chemical shifts were referenced to the internal standard of tetramethylsilane (as $\delta = 0.00$ ppm). High-resolution electrospray ionization (ESI) mass spectra were obtained on a Thermo Scientific LTQ Orbitrap XL mass spectrometer. FT-IR spectra were recorded on a Shimadzu IRTracer-100 FT-IR Spectrophotometer. Electronic absorption spectra were recorded on an Agilent 8453 UV–vis spectrophotometer with ChemStation software.

Elemental Analysis. Elemental analysis was performed by a PerkinElmer 2400 Series II CHNS/O Analyzer. The metal complexes can crystallize with substantial solvent-accessible void space and retain small amounts of residual solvent even after repeated drying under conditions that avoid decomposition. Because of the paramagnetic nature of the complexes, the identity and amount of such residual solvent cannot be reliably quantified by conventional NMR spectroscopy. Accordingly, the fractional solvent compositions included with the elemental analysis data should be regarded as empirical formulations consistent with the observed analytical values, rather than as uniquely established solvate stoichiometries. They are provided to indicate the likely contribution of retained solvent to the elemental analysis results.

Electrochemistry. Electrochemical studies were carried out with an Autolab model PGSTAT204 (Metrohm). A three-electrode configuration was used, consisting of a working electrode (platinum button electrode), a $Ag/AgNO_3$ (0.05 M in CH_3CN with 0.1 M n-Bu_4NPF_6) reference electrode, and a platinum coil counter electrode. All electrochemical potentials were reported with respect to the Cp_2Fe/Cp_2Fe^+ redox couple unless otherwise noted.

Electrochemical Control Experiments. To compare the effects of intramolecular vs. intermolecular hydrogen bonding on $Fe^{II/III}$ redox chemistry, CV experiments were performed on **1f**, a complex lacking pendant pyridine groups, in the presence of exogenous pyridine and compared with **1b** (Fig. S22). Complex **1f** exhibits $E_{1/2} = +0.40$ V, and addition of 4 and 10 equiv. of pyridine shifts this value cathodically to +0.15 and +0.12 V, respectively. Although the total shift of 0.28 V approaches the $E_{1/2}$ of **1b** (+0.10 V), the intramolecular design of **1b** produces a more pronounced effect. This is notable because the covalently tethered pyridine pendants in **1b** also function as electron-withdrawing substituents on the benzimidazole core, partially offsetting the electron-donating effect of the intramolecular HBs.

[1] T. Zhou, R. Hu, L. Wang, Y. Qiu, G. Zhang, Q. Deng, H. Zhang, P. Yin, B. Situ, C. Zhan, A. Qin and B. Z. Tang, *Angew. Chem. Int. Ed.*, 2020, **59**, 9952–9956.

[2] S. Kaide, M. Ono, H. Watanabe, A. Kitada, M. Yoshimura, Y. Shimizu, M. Ihara and H. Saji, *ACS Med. Chem. Lett.*, 2018, **9**, 478–483.

[3] L. M. Thierer, Q. Wang, S. H. Brooks, P. Cui, J. Qi, M. R. Gau, B. C. Manor, P. J. Carroll and N. C. Tomson, *Polyhedron*, 2021, **198**, 115044.

Beyond this thermodynamic effect, addition of exogenous pyridine to **1f** compromises electrochemical reversibility, with ΔE_p increasing from 250 to 360 mV and both the anodic and cathodic waves becoming broader. These observations are consistent with intermolecular HBD–HBA association accompanied by coupled proton transfer and speciation changes on the CV timescale. Upon oxidation to Fe^{III}, proton transfer from the benzimidazole N–H group to pyridine may become favorable, and the reverse process would then require proton back-transfer and structural reorganization to regenerate the initial Fe^{II} species. Because the external pyridine and pyridinium species are not covalently constrained, diffusional separation, intermolecular reorganization, and the formation of multiple association or protonation states may impede this return process. In contrast, treatment of **1b** and **1d** with excess pyridine results in only marginal shifts in $E_{1/2}$ (Fig. S23; $\Delta E_{1/2} = -32$ mV for **1b** and +10 mV for **1d**), with no appreciable change in ΔE_p. These observations suggest that the intramolecular N–H$\cdots$N hydrogen-bonding arrangement remains largely intact in the presence of a competing external HBA. The covalently enforced intramolecular scaffold thus amplifies the thermodynamic effect while preserving electrochemical reversibility, highlighting the importance of geometric preorganization in the secondary coordination sphere.

2. Synthetic Procedures

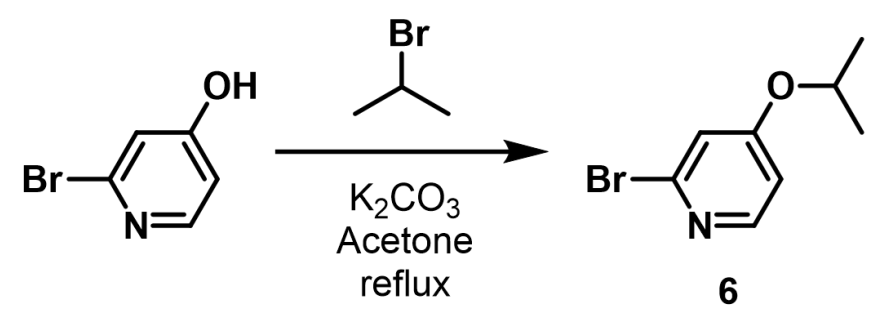

2-Bromo-4-isopropoxypyridine (6). A 100 mL round-bottom flask was charged with 2-bromopropane (1.82 g, 14.8 mmol) and acetone (30 mL). To the solution were added 2-bromopyridin-4-ol (1.20 g, 6.93 mmol) and K_2CO_3 (2.24 g, 16.2 mmol). The reaction mixture was heated at reflux for 18 h. After cooling to r.t., the mixture was diluted with EtOAc (100 mL), and washed with brine (50 mL × 3). The organic layer was dried over anhyd $MgSO_4$, filtered, and concentrated under reduced pressure. The residual material was purified by flash column chromatography on SiO_2 (hexane:EtOAc = 100:0 to 75:25, v/v) to furnish **6** as a colorless oil (1.37 g, 6.34 mmol, yield = 91%). ^1H NMR (500 MHz, $CDCl_3$, 298 K) δ 8.13 (d, J = 5.0 Hz, 1H), 6.96 (s, 1H), 6.74 (d, J = 5.0 Hz, 1H), 4.61 (m, J = 7.0 Hz, 1H), 1.36 (d, J = 7.0 Hz, 6H). ^{13}C NMR (125 MHz, $CDCl_3$, 298 K) δ 165.31, 150.62, 142.95, 114.35, 111.03, 70.84, 21.60. FT-IR (ATR, cm^{-1}): 3059, 2979, 2934, 1577, 1543, 1467, 1382, 1301, 1267, 1254, 1231, 1179, 1140, 1105, 983, 959, 882, 835, 818, 704, 696. HRMS (ESI) calcd. for $C_8H_{10}BrNONa$ $[M + Na]^+$ 237.9838, found 237.9842.

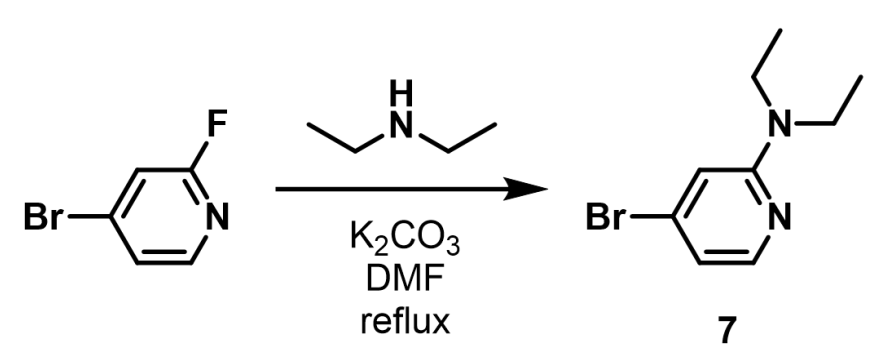

4-Bromo-*N*,*N*-diethylpyridin-2-amine (7). A 250 mL round-bottom flask was charged with 4-bromo-2-fluoropyridine (3.42 g, 19.5 mmol) and DMF (50 mL). To the solution were added diethylamine (2.86 g, 39.1 mmol) and K_2CO_3 (5.40 g, 38.9 mmol). The reaction mixture was heated at reflux for 26 h, cooled to r.t., and concentrated under reduced pressure. The residual material was extracted into EtOAc (50 mL) and washed with brine (50 mL × 3). The organic layer was dried over anhyd $MgSO_4$, filtered, and concentrated under reduced pressure. Flash column chromatography on SiO_2 (hexane:DCM = 10:0 to 8:2 v/v) furnished **7** as a yellow oil (2.17 g, 9.46 mmol, yield = 49%). ^1H NMR (500 MHz, $CDCl_3$, 298 K) δ 7.94 (d, J = 5.3 Hz, 1H), 6.62 (dd, J = 5.3, 1.5 Hz, 1H), 6.60 (d, J = 1.3 Hz, 1H), 3.48 (q, J = 7.1 Hz, 4H), 1.17 (t, J = 7.1 Hz, 6H). ^{13}C NMR (125 MHz, $CDCl_3$, 298 K) δ 158.27, 148.91, 133.28, 114.01, 108.08, 42.59, 12.82. FT-IR (ATR, cm^{-1}): 2971, 2928, 2869, 1578, 1533, 1495, 1471, 1428, 1356, 1277, 1198, 1078, 1020, 970, 924, 823, 785, 758, 681. HRMS (ESI) calcd. for $C_9H_{14}BrN_2$ $[M + H]^+$ 229.0335; found 229.0343.

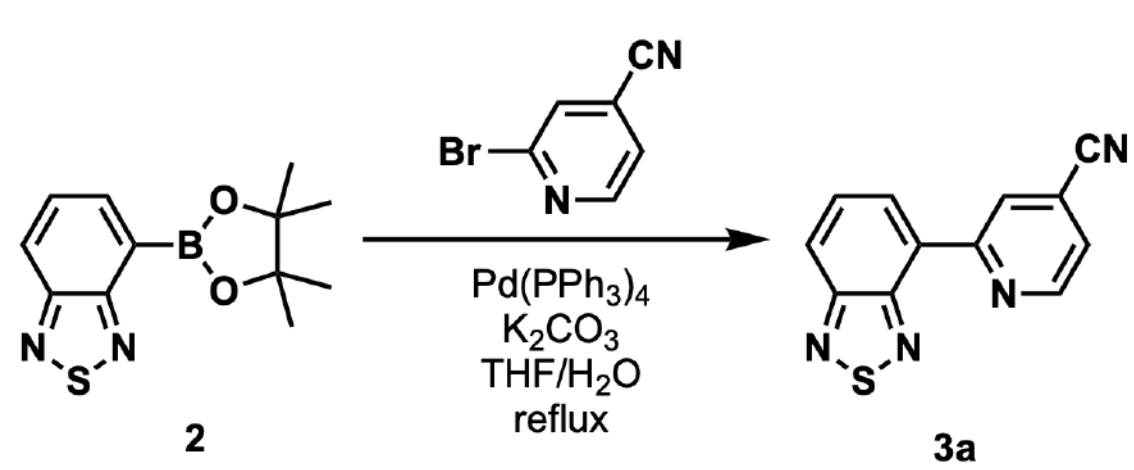

2-(Benzo[*c*][1,2,5]thiadiazol-4-yl)isonicotinonitrile (3a). A 250 mL 3-neck round-bottom flask was charged with 2-bromoisonicotinonitrile (1.40 g, 7.65 mmol), **2** (1.06 g, 4.04 mmol),

$Pd(PPh_3)_4$ (0.233 g, 0.202 mmol), and K_2CO_3 (2.00 g, 14.5 mmol) under an Ar atmosphere. To the mixture were added degassed THF (30 mL) and H_2O (5 mL). The reaction was heated at reflux for 24 h. After cooling to r.t., volatile fractions were removed under reduced pressure, and the residual material was diluted with CH_2Cl_2 (50 mL) and H_2O (50 mL). The aqueous phase was extracted into CH_2Cl_2 (50 mL × 3). The combined organic fractions were dried over anhyd $MgSO_4$, filtered, and concentrated under reduced pressure. Flash column chromatography on SiO_2 (hexane:EtOAc = 100:0 to 85:15, v/v) furnished **3a** as a yellow solid (0.960 g, 4.02 mmol, yield = 99%). ^{1}H NMR (500 MHz, $CDCl_3$, 298 K) δ 9.15 (t, J = 1.2 Hz, 1H), 8.93 (dd, J = 4.9, 1.0 Hz, 1H), 8.60 (dd, J = 7.1, 1.2 Hz, 1H), 8.15 (dd, J = 8.8, 1.1 Hz, 1H), 7.79 (dd, J = 8.7, 7.1 Hz, 1H), 7.55 (dd, J = 4.9, 1.5 Hz, 1H). ^{13}C NMR (125 MHz, $CDCl_3$, 298 K) δ 155.74, 155.12, 152.49, 150.41, 129.86, 129.70, 129.57, 126.51, 124.15, 123.48, 121.14, 116.86. FT-IR (ATR, cm^{-1}): 3097, 3066, 2237, 1600, 1587, 1553, 1538, 1469, 1413, 1382, 1329, 1268, 1126, 1102, 970, 843, 809, 750. HRMS (ESI) calcd. for $C_{12}H_7N_4S$ $[M + H]^+$ 239.0386, found 239.0388.

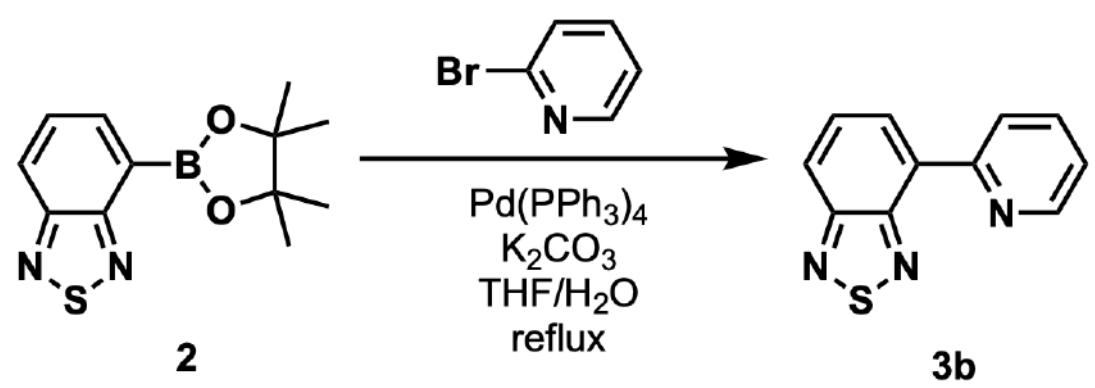

4-(Pyridin-2-yl)benzo[*c*][1,2,5]thiadiazole (3b). A 100 mL round-bottom flask was charged with 2-bromopyridine (0.362 g, 2.29 mmol), **2** (0.392 g, 1.50 mmol), $Pd(PPh_3)_4$ (0.173 g, 0.150 mmol), and K_2CO_3 (0.620 g, 4.49 mmol) under an Ar atmosphere. To the mixture were added degassed THF (20 mL) and H_2O (4 mL). The reaction was heated at reflux for 19 h. After cooling to r.t., volatile fractions were removed under reduced pressure, and the residual material was diluted with EtOAc (20 mL) and H_2O (20 mL). The aqueous phase was extracted into EtOAc (20 mL × 3). The combined organic fractions were dried over anhyd $MgSO_4$, filtered, and concentrated under reduced pressure. Flash column chromatography on SiO_2 (hexane:EtOAc = 100:0 to 50:50, v/v) furnished **3b** as a yellow solid (0.313 g, 1.47 mmol, yield = 98%). ^{1}H NMR (500 MHz, $CDCl_3$, 298 K) δ 8.79 (ddd, J = 4.8, 1.9, 0.9 Hz, 1H), 8.63 (dt, J = 8.0, 1.1 Hz, 1H), 8.42 (dd, J = 7.0, 1.2 Hz, 1H), 8.07 (dd, J = 8.7, 1.1 Hz, 1H), 7.87 (td, J = 7.7, 1.9 Hz, 1H), 7.75 (dd, J = 8.7, 7.0 Hz, 1H), 7.34 (ddd, J = 7.5, 4.8, 1.1 Hz, 1H). ^{13}C NMR (125 MHz, $CDCl_3$, 298 K) δ 155.80, 154.18, 152.96, 149.77, 136.59, 132.24, 129.70, 129.25, 124.97, 123.05, 122.11. FT-IR (ATR, cm^{-1}): 3054, 3005, 2922, 1583, 1564, 1536, 1465, 1435, 1326, 1265, 1168, 1095, 993, 900, 855, 833, 775, 752. HRMS (ESI) calcd. for $C_{11}H_8N_3S$ $[M + H]^+$ 214.0434, found 214.0434.

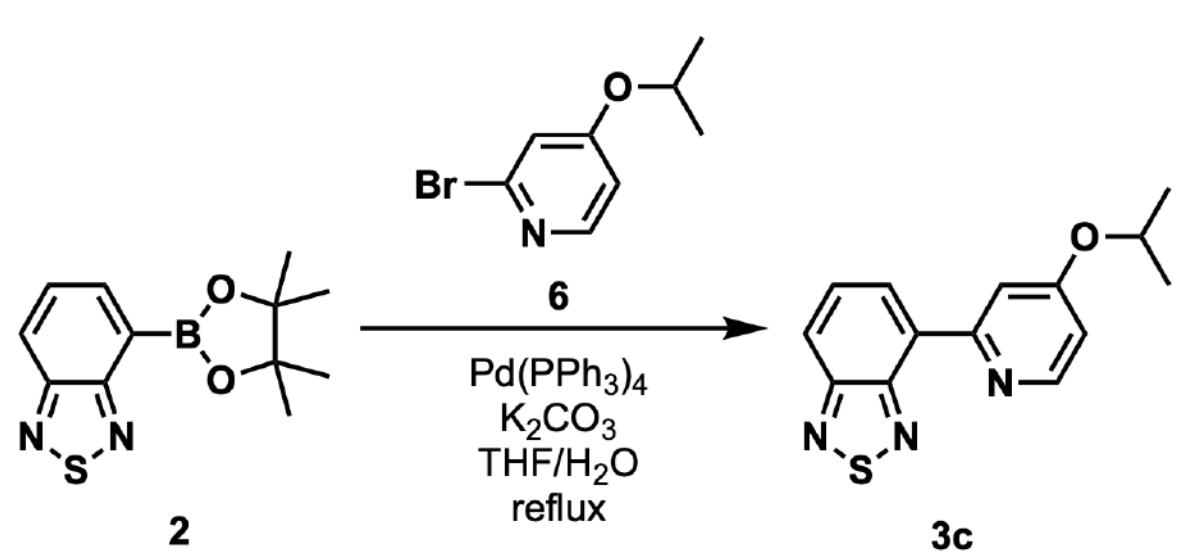

4-(4-Isopropoxypyridin-2-yl)benzo[*c*][1,2,5]thiadiazole (3c). A 100 mL round-bottom flask was charged with **6** (3.00 g, 13.9 mmol), **2** (3.56 g, 13.6 mmol), $Pd(PPh_3)_4$ (0.600 g, 0.519 mmol), and K_2CO_3 (6.00 g, 43.4 mmol) under an Ar atmosphere. To the mixture were added degassed THF (80 mL) and H_2O (15 mL). The reaction was heated at reflux for 19 h. After cooling to r.t.,

volatile fractions were removed under reduced pressure, and the residual material was diluted with EtOAc (50 mL) and H_2O (50 mL). The aqueous phase was extracted into EtOAc (50 mL × 3). The combined organic fractions were dried over anhyd $MgSO_4$, filtered, and concentrated under reduced pressure. Flash column chromatography on SiO_2 (hexane:EtOAc = 100:0 to 50:50, v/v) furnished **3c** as a yellow oil (3.35 g, 12.3 mmol, yield = 90%). ^1H NMR (500 MHz, $CDCl_3$, 298 K) δ 8.56 (d, J = 5.7 Hz, 1H), 8.43 (dd, J = 7.1, 1.0 Hz, 1H), 8.22 (d, J = 2.4 Hz, 1H), 8.07 (dd, J = 8.7, 1.0 Hz, 1H), 7.74 (dd, J = 8.7, 7.1 Hz, 1H), 6.83 (dd, J = 5.7, 2.5 Hz, 1H), 4.79 (m, J = 6.1 Hz, 1H), 1.45 (d, J = 6.1 Hz, 6H). ^{13}C NMR (125 MHz, $CDCl_3$, 298 K) δ 164.64, 155.81, 155.58, 152.94, 150.80, 132.25, 129.67, 129.23, 122.06, 112.51, 110.27, 70.20, 21.84. FT-IR (ATR, cm^{-1}): 3062, 2976, 2933, 1740, 1583, 1562, 1469, 1374, 1305, 1226, 1111, 1002, 991, 945, 876, 857, 833, 812, 754, 719, 666. HRMS (ESI) calcd. for $C_{14}H_{14}N_3OS$ $[M + H]^+$ 271.0852, found 271.0853.

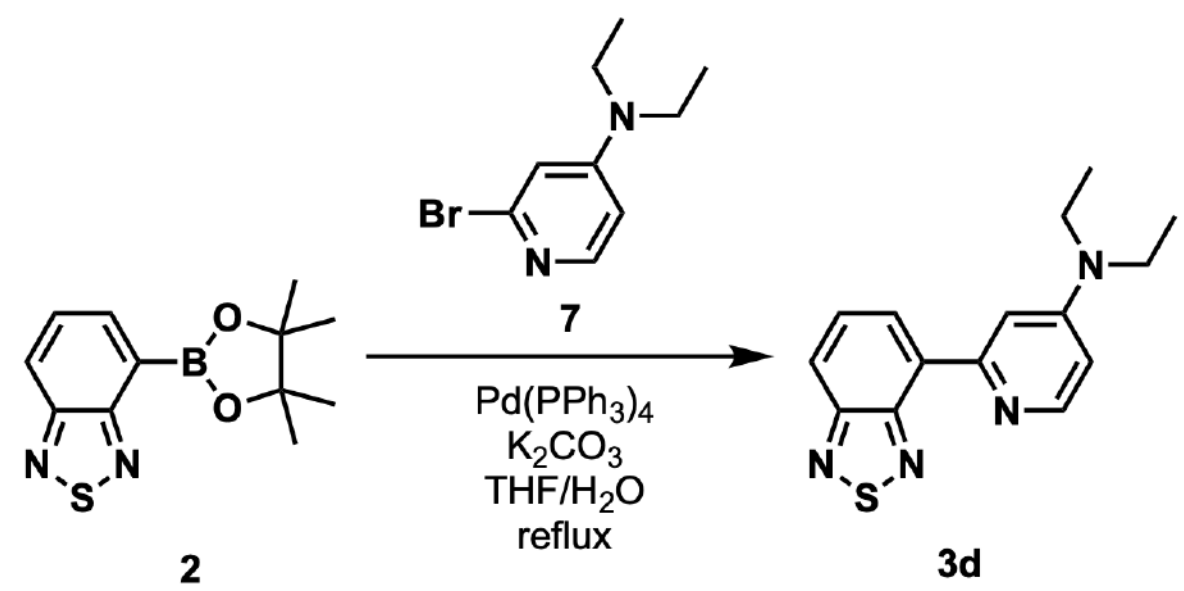

2-(Benzo[*c*][1,2,5]thiadiazol-4-yl)-*N*,*N*-diethylpyridin-4-amine (3d). A 500 mL round-bottom flask was charged with **7** (3.87 g, 16.9-mmol), **2** (4.02 g, 15.4 mmol), $Pd(PPh_3)_4$ (0.913 g, 0.769 mmol), and K_2CO_3 (6.41 g, 46.4 mmol) under an Ar atmosphere. To the mixture were added degassed THF (130 mL) and H_2O (26 mL). The reaction was heated at reflux for 19 h. After cooling to r.t., volatile fractions were removed under reduced pressure, and the residual material was diluted with EtOAc (100 mL) and H_2O (100 mL). The organic phase was washed with brine (50 mL × 3), dried over anhyd $MgSO_4$, filtered, and concentrated under reduced pressure. Flash column chromatography on SiO_2 (hexane:EtOAc = 100:0 to 0:100, v/v) furnished **3d** as a brown oil (3.54 g, 12.4 mmol, yield = 81%). ^1H NMR (500 MHz, $CDCl_3$, 298 K) δ 8.37 (s, 1H), 8.35 (s, 1H), 8.03 (dd, J = 8.7, 1.1 Hz, 1H), 7.86 (d, J = 2.6 Hz, 1H), 7.72 (dd, J = 8.7, 7.0 Hz, 1H), 6.52 (dd, J = 6.0, 2.7 Hz, 1H), 3.47 (q, J = 7.1 Hz, 4H), 1.26 (t, J = 7.1 Hz, 6H). ^{13}C NMR (125 MHz, $CDCl_3$, 298 K) δ 155.80, 154.32, 153.18, 152.61, 149.89, 133.53, 129.72, 128.93, 121.44, 108.16, 105.53, 44.15, 12.46. FT-IR (ATR, cm^{-1}): 2970, 2930, 2894, 2870, 1601, 1583, 1538, 1494, 1476, 1357, 1285, 1244, 1197, 1022, 983, 896, 855, 832, 805, 754. HRMS (ESI) calcd. for $C_{15}H_{17}N_4S$ $[M + H]^+$ 285.1169, found 285.1171.

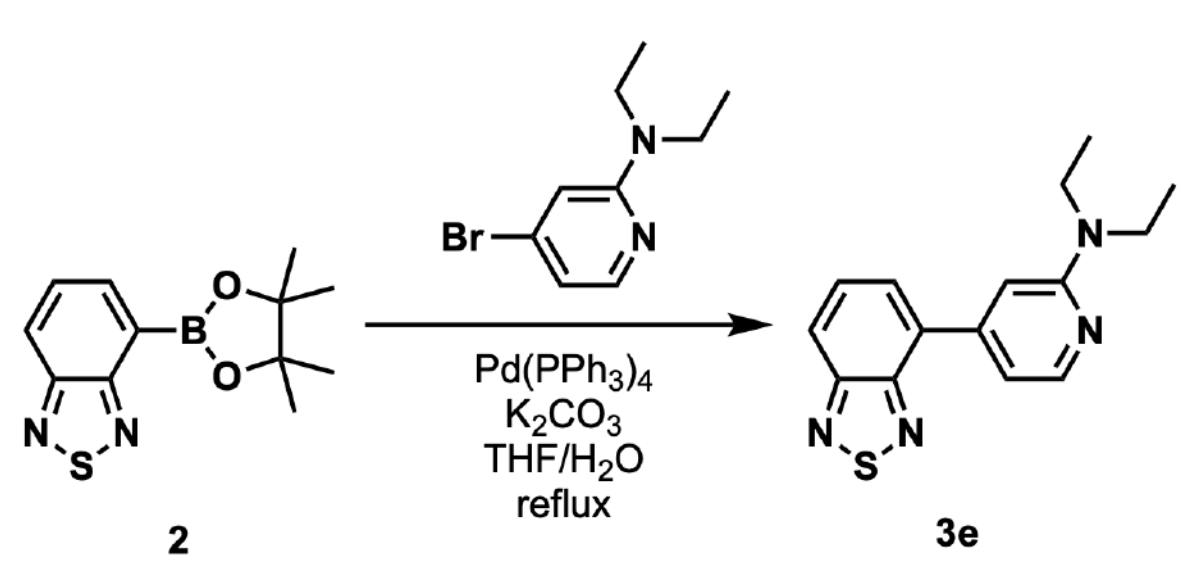

4-(Benzo[*c*][1,2,5]thiadiazol-4-yl)-*N*,*N*-diethylpyridin-2-amine (3e). A 500 mL round-bottom flask was charged with 4-bromo-*N*,*N*-diethylpyridin-2-amine (2.17 g, 9.46-mmol), **2** (2.75 g, 10.5 mmol), $Pd(PPh_3)_4$ (0.572 g, 0.495 mmol), and K_2CO_3 (3.92 g, 28.4 mmol) under an Ar

atmosphere. To the mixture were added degassed THF (100 mL) and H_2O (20 mL). The reaction was heated at reflux for 12 h. After cooling to r.t., volatile fractions were removed under reduced pressure, and the residual material was diluted with EtOAc (100 mL) and H_2O (100 mL). The organic phase was washed with brine (50 mL × 3), dried over anhyd $MgSO_4$, filtered, and concentrated under reduced pressure. Flash column chromatography on SiO_2 (hexane:EtOAc = 100:0 to 20:80, v/v) furnished **3e** as an orange oil (2.69 g, 9.46 mmol, yield = quant.). ^{1}H NMR (500 MHz, $CDCl_3$, 298 K) δ 8.29 (d, J = 5.2 Hz, 1H), 8.04 (dd, J = 8.7, 1.1 Hz, 1H), 7.74 (dd, J = 6.9, 1.1 Hz, 1H), 7.69 (dd, J = 8.7, 6.9 Hz, 1H), 7.07 (s, 1H), 6.95 (dd, J = 5.2, 1.4 Hz, 1H), 3.60 (q, J = 7.1 Hz, 4H), 1.25 (t, J = 7.1 Hz, 6H). ^{13}C NMR (125 MHz, $CDCl_3$, 298 K) δ 157.94, 155.55, 153.18, 148.45, 145.63, 133.41, 129.46, 127.89, 121.48, 111.12, 106.00, 42.73, 13.06. FT-IR (ATR, cm^{-1}): 2970, 2928, 1596, 1540, 1495, 1431, 1359, 1260, 1203, 1159, 1030, 970, 894, 853, 799, 754. HRMS (ESI) calcd. for $C_{15}H_{17}N_4S$ $[M + H]^+$ 285.1169, found 285.1170.

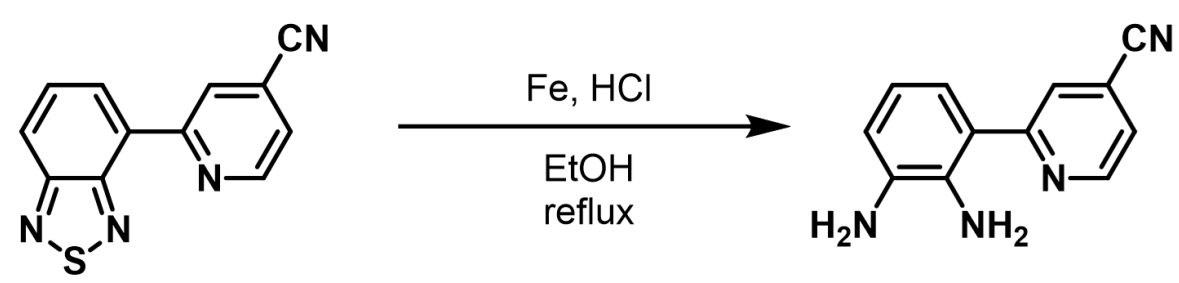

3a 4a

2-(2,3-Diaminophenyl)isonicotinonitrile (4a). A 250 mL round-bottom flask was charged with **3a** (0.960 g, 4.02 mmol), conc. HCl (6 mL), and EtOH (25 mL). To the flask was added Fe powder (1.70 g, 30.4 mmol). The reaction mixture was heated at reflux for 1.5 h under an Ar atmosphere. After cooling to 0 °C, the reaction mixture was neutralized by adding aq NaOH (2.60 g, 65 mmol) and aq Na_4EDTA (20.0 g, 52.6 mmol). The mixture was filtered through a pack of Celite, and washed thoroughly with CH_2Cl_2 (100 mL). The filtrate was extracted into CH_2Cl_2 (100 mL × 3). The combined organic extracts were dried over anhyd $MgSO_4$, filtered, and concentrated under reduced pressure. Flash column chromatography on SiO_2 (hexane:EtOAc = 100:0 to 66:34, v/v) furnished **4a** as an orange solid (0.510 g, 2.43 mmol, yield = 53%). ^{1}H NMR (500 MHz, $CDCl_3$, 298 K) δ 8.75 (d, J = 5.0 Hz, 1H), 7.89 (s, 1H), 7.35 (dd, J = 5.0, 1.3 Hz, 1H), 7.08 (dd, J = 7.9, 1.2 Hz, 1H), 6.80 (dd, J = 7.6, 1.3 Hz, 1H), 6.74 (t, J = 7.8 Hz, 1H), 5.51 (br, 2H), 3.48 (br, 2H). ^{13}C NMR (125 MHz, $CDCl_3$, 298 K) δ 160.96, 148.75, 136.68, 135.42, 124.20, 121.59, 121.22, 120.83, 120.79, 118.45, 118.25, 116.81. FT-IR (ATR, cm^{-1}): 3404, 3331, 3062, 2237, 1591, 1542, 1486, 1451, 1388, 1290, 1234, 1173, 1104, 990, 894, 839, 781, 731. HRMS (ESI) calcd. for $C_{12}H_{11}N_4$ $[M + H]^+$ 211.0978, found. 211.0981.

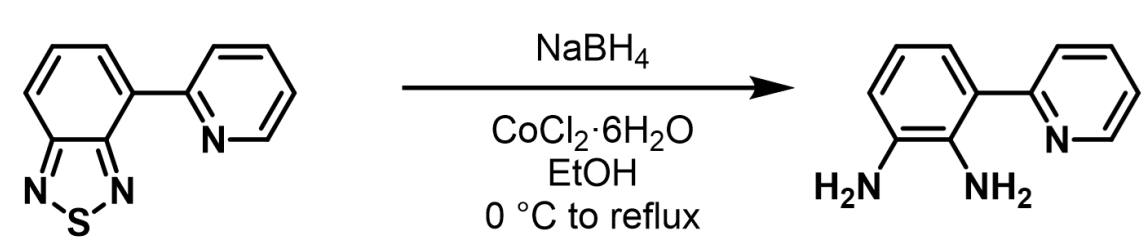

3b 4b

3-(Pyridin-2-yl)benzene-1,2-diamine (4b). A 250 mL round-bottom flask was charged with **3b** (0.742 g, 3.48 mmol), $CoCl_2\cdot6H_2O$ (95.3 mg, 0.401 mmol), and EtOH (50 mL). The solution was cooled to 0 °C, and $NaBH_4$ (1.30 g, 34.8 mmol) was added slowly. The reaction mixture was heated at reflux for 3 h under an Ar atmosphere. The mixture was cooled to 0 °C, and H_2O (50 mL) was added carefully to quench the remaining $NaBH_4$. The mixture was filtered through a pack of Celite, and washed thoroughly with EtOAc (100 mL). The organic phase was washed with brine (50 mL × 3), dried over anhyd $MgSO_4$, filtered, and concentrated under reduced pressure. Flash column chromatography on SiO_2 (hexane:EtOAc = 100:0 to 50:50, v/v) furnished **4b** as an orange oil (0.432 g, 2.33 mmol, yield = 67%). ^{1}H NMR (500 MHz, $CDCl_3$, 298 K) δ 8.61 (d, J =

4.1 Hz, 1H), 7.72 (td, J = 7.8, 1.8 Hz, 1H), 7.64 (d, J = 8.1 Hz, 1H), 7.15 (dd, J = 6.9, 5.3 Hz, 1H), 7.09 (dd, J = 7.1, 2.2 Hz, 1H), 6.73 (d, J = 2.1 Hz, 1H), 6.71 (d, J = 7.6 Hz, 1H), 5.33 (br, 2H), 3.52 (br, 2H). ^{13}C NMR (125 MHz, $CDCl_3$, 298 K) δ 159.64, 147.92, 136.93, 135.86, 135.39, 123.52, 122.66, 121.01, 120.98, 118.31, 117.10. FT-IR (ATR, cm^{-1}): 3392, 3323, 3052, 3009, 1610, 1585, 1558, 1473, 1455, 1422, 1293, 1228, 1153, 1096, 993, 912, 762, 737. HRMS (ESI) calcd. for $C_{11}H_{12}N_3$ [M + H]$^+$ 186.1026, found. 186.1026.

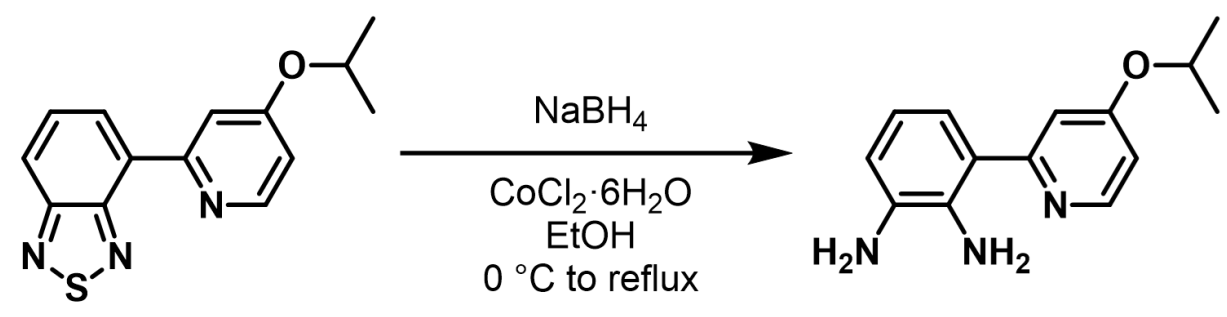

3c 4c

3-(4-Isopropoxypyridin-2-yl)benzene-1,2-diamine (4c). A 250 mL round-bottom flask was charged with **3c** (0.770 g, 2.84 mmol), $CoCl_2{\cdot}6H_2O$ (135 mg, 0.567 mmol), and EtOH (30 mL). The solution was cooled to 0 °C, and $NaBH_4$ (1.07 g, 28.3 mmol) was added slowly. The reaction mixture was heated at reflux for 2 h under an Ar atmosphere. The mixture was cooled to 0 °C, and H_2O (50 mL) was added carefully to quench the remaining $NaBH_4$. The mixture was filtered through a pack of Celite, and washed thoroughly with CH_2Cl_2 (50 mL). The filtrate was extracted into CH_2Cl_2 (50 mL × 3). The combined extracts were dried over anhyd $MgSO_4$, filtered, and concentrated under reduced pressure. Flash column chromatography on SiO_2 (hexane:EtOAc = 100:0 to 50:50, v/v) furnished **4c** as an orange oil (0.450 g, 1.85 mmol, yield = 65%). ^{1}H NMR (500 MHz, $CDCl_3$, 298 K) δ 8.42 (d, J = 5.8 Hz, 1H), 7.11 (d, J = 2.4 Hz, 1H), 7.04 (dd, J = 7.6, 1.6 Hz, 1H), 6.76 (dd, J = 7.6, 1.6 Hz, 1H), 6.73 (d, J = 7.6 Hz, 1H), 6.70 (dd, J = 5.8, 2.4 Hz, 1H), 5.33 (br, 2H), 4.70 (m, J = 6.1 Hz, 1H), 3.44 (br, 2H), 1.39 (d, J = 6.1 Hz, 6H). ^{13}C NMR (125 MHz, $CDCl_3$, 298 K) δ 164.85, 161.27, 149.37, 135.74, 135.29, 124.11, 120.93, 118.28, 116.95, 109.64, 108.98, 69.90, 21.84. FT-IR (ATR, cm^{-1}): 3400, 3327, 2977, 2932, 1588, 1554, 1455, 1404, 1384, 1294, 1258, 1233, 1106, 994, 980, 913, 865, 822, 786, 745. HRMS (ESI) calcd. for $C_{14}H_{18}N_3O$ [M + H]$^+$ 244.1444, found 244.1444.

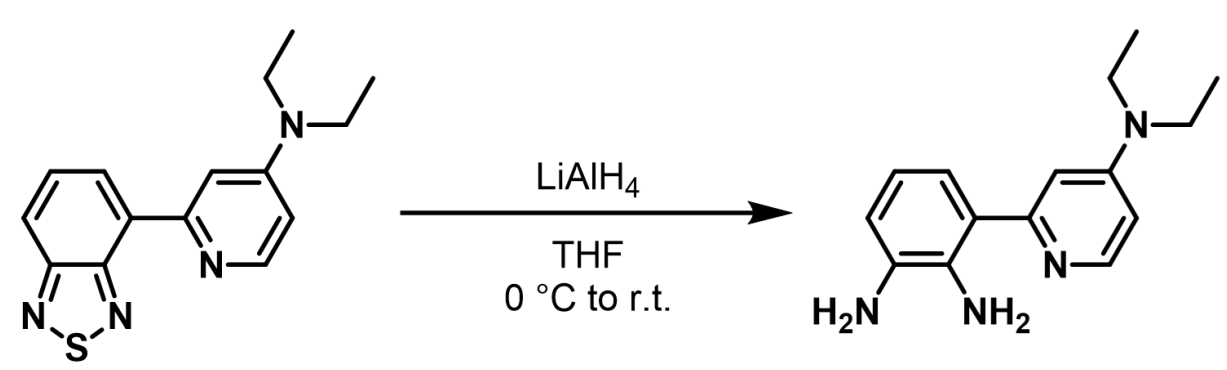

3d 4d

3-(4-(Diethylamino)pyridin-2-yl)benzene-1,2-diamine (4d). A 500 mL round-bottom flask was charged with **3d** (3.54 g, 12.6 mmol) under an Ar atmosphere. To the flask was added degassed THF (100 mL). The solution was cooled to 0 °C, and a THF solution of $LiAlH_4$ (25 mL, 50 mmol) was added dropwise. The reaction mixture was stirred at r.t. for 1 h under an Ar atmosphere. The mixture was cooled to 0 °C, and treated slowly with H_2O (2 mL), aq NaOH (15wt%, 2 mL), and H_2O (6 mL) to quench the remaining $LiAlH_4$. The mixture was dried over anhyd $MgSO_4$, filtered through a pack of Celite, and washed thoroughly with EtOAc (100 mL). The filtrate was concentrated under reduced pressure. Flash column chromatography on SiO_2 (EtOAc:MeOH = 100:0 to 75:25, v/v) furnished **4d** as a yellow solid (2.86 g, 11.1 mmol, yield = 88%). ^{1}H NMR (500 MHz, $CDCl_3$, 298 K) δ 8.22 (d, J = 6.0 Hz, 1H), 7.00 (dd, J = 6.7, 2.6 Hz, 1H), 6.74 (d, J = 2.6 Hz, 1H), 6.72 (s, 1H), 6.72 – 6.69 (m, 1H), 6.41 (dd, J = 6.1, 2.6 Hz, 1H), 5.12 (br, 2H), 3.39 (q, J = 7.1 Hz, 4H), 1.20 (t, J = 7.1 Hz, 6H). ^{13}C NMR (125 MHz, $CDCl_3$, 298 K) δ 159.88, 152.96,

148.56, 135.32, 135.19, 126.00, 120.92, 118.25, 116.45, 105.40, 104.49, 43.88, 12.43. FT-IR (ATR, cm^{-1}): 3409, 3335, 3230, 2970, 2928, 2869, 1594, 1536, 1496, 1469, 1433, 1375, 1360, 1267, 1201, 1160, 1094, 1079, 1023, 984, 925, 886, 848, 814, 780, 736. HRMS (ESI) calcd. for $C_{15}H_{21}N_4$ $[M + H]^+$ 257.1761, found 257.1764.

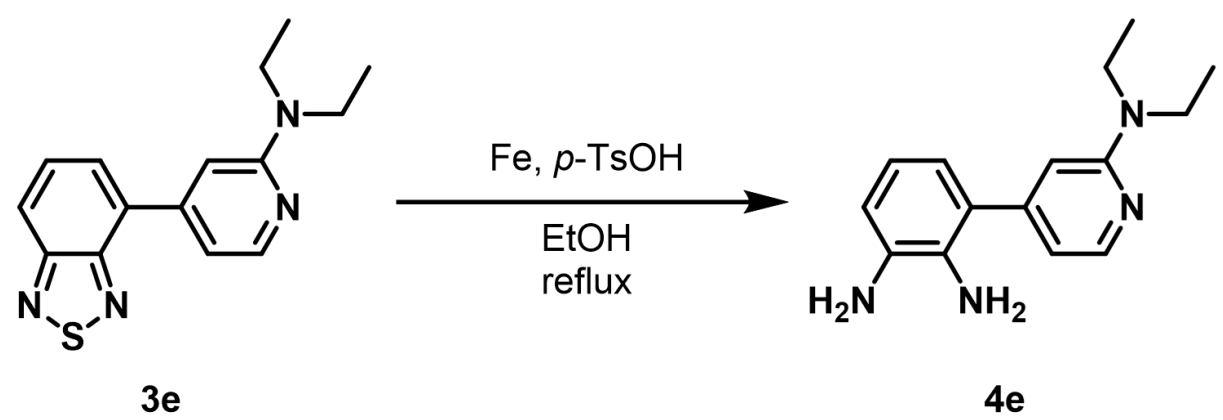

3-(2-(Diethylamino)pyridin-4-yl)benzene-1,2-diamine (4e). A 500 mL round-bottom flask was charged with **3e** (2.69 g, 9.46 mmol), *p*-TsOH (36.0 g 189 mmol), and EtOH (150 mL). To the flask was added Fe powder (1.70 g, 30.4 mmol) at r.t. The reaction mixture was heated at reflux for 2 h under an Ar atmosphere. After cooling to 0 °C, the reaction mixture was neutralized by the addition of aq NaOH (7.56 g, 189 mmol) and aq Na_4EDTA (21.6 g, 56.8 mmol). The mixture was filtered through a pack of Celite and washed thoroughly with EtOAc (150 mL). The organic phase was washed with sat'd aq K_2CO_3 (100 mL), dried over anhyd $MgSO_4$, filtered, and concentrated under reduced pressure. Flash column chromatography on SiO_2 (hexane:EtOAc = 100:0 to 50:50, v/v) furnished **4e** as an orange solid (1.66 g, 6.41 mmol, yield = 68%). ^1H NMR (500 MHz, $CDCl_3$, 298 K) δ 8.20 (d, J = 5.1 Hz, 1H), 6.78–6.71 (m, 3H), 6.57 (dd, J = 5.1, 1.3 Hz, 1H), 6.50 (s, 1H), 3.54 (q, J = 7.1 Hz, 4H), 1.19 (t, J = 7.1 Hz, 6H). ^{13}C NMR (125 MHz, $CDCl_3$, 298 K) δ 158.06, 148.65, 148.61, 134.79, 132.27, 128.02, 120.99, 119.46, 116.36, 111.54, 105.58, 42.58, 13.05. FT-IR (ATR, cm^{-1}): 3409, 3335, 3230, 2970, 2929, 1594, 1536, 1496, 1469, 1433, 1360, 1267, 1201, 1160, 1094, 1023, 945, 848. HRMS (ESI) calcd. for $C_{15}H_{21}N_4$ $[M + H]^+$ 257.1761, found 257.1761.

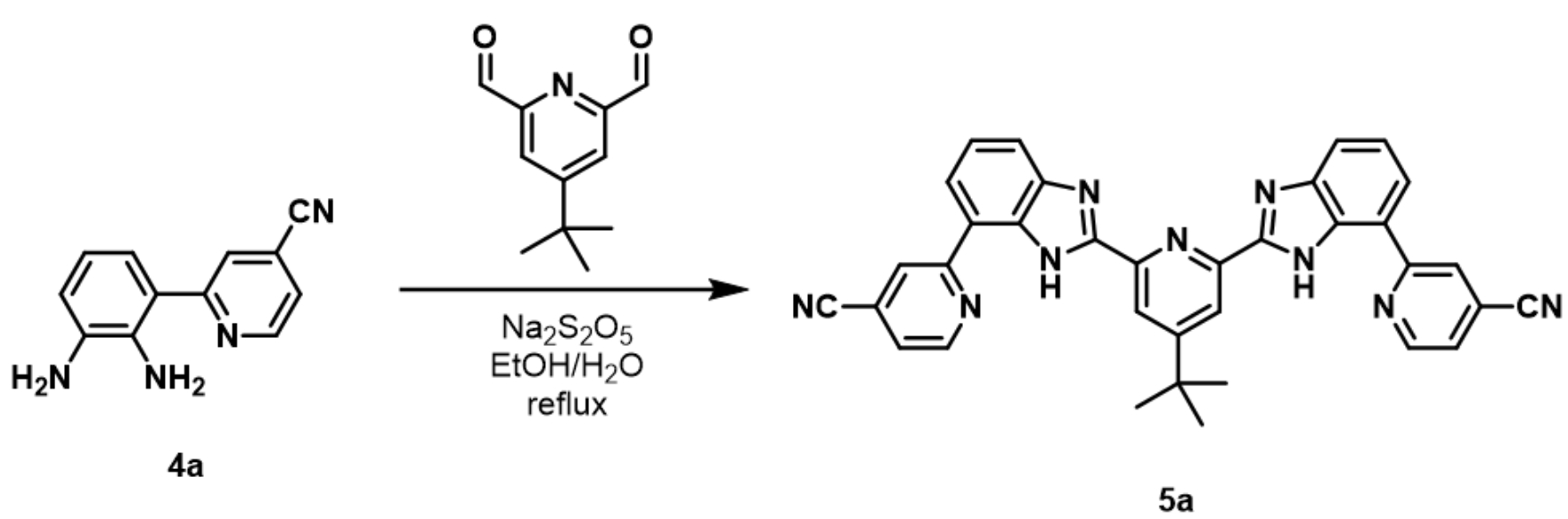

2,2′-((4-(*tert*-Butyl)pyridine-2,6-diyl)bis(1*H*-benzo[*d*]imidazole-2,7-diyl))diisonicotinonitrile (5a). A 100 mL round-bottom flask was charged with **4a** (1.12 g, 5.33 mmol) and 4-(*tert*-butyl)pyridine-2,6-dicarbaldehyde (0.438 g, 2.29 mmol). A portion of anhyd EtOH (40 mL) was added, and the mixture was stirred at 50 °C. To the solution was added aqueous solution (2 mL) of $Na_2S_2O_5$ (0.566 g, 2.98 mmol). The reaction mixture was heated at reflux for 24 h under an Ar atmosphere. After cooling to r.t., volatile fractions were removed under reduced pressure, and the residual material was diluted with CH_2Cl_2 (50 mL) and H_2O (50 mL). The organic phase was washed with brine (50 mL × 3), dried over anhyd $MgSO_4$, filtered, and concentrated under reduced pressure. The residual material was suspended in MeCN, and the precipitate was isolated by filtration to afford **5a** as a beige solid (0.585 g, 1.02 mmol, yield = 44%). ^1H NMR (500 MHz, $CDCl_3$, 298 K) δ 12.43 (br, 2H), 8.73 (d, J = 4.9 Hz, 2H), 8.55 (s, 2H), 8.33 (s, 2H), 8.07 (d, J = 8.0 Hz, 2H), 7.93 (d, J = 7.7 Hz, 2H), 7.49 (t, J = 7.8 Hz, 2H), 7.39 (d, J = 4.7 Hz, 2H), 1.52 (s, 9H). ^{13}C NMR (125 MHz, $CDCl_3$, 298 K) δ 163.51, 158.24, 151.78, 149.98,

148.12, 145.96, 133.11, 123.22, 122.95, 122.32, 121.92, 121.89, 121.35, 120.31, 120.11, 116.39, 35.62, 30.61. FT-IR (ATR, cm^{-1}): 3422, 3063, 2966, 2238, 1597, 1545, 1469, 1447, 1421, 1349, 1310, 1285, 1266, 1204, 1164, 1129, 1091, 867, 834, 736. HRMS (ESI) calcd. for $C_{35}H_{26}N_9$ [M + H]$^+$ 572.2306, found 572.2309.

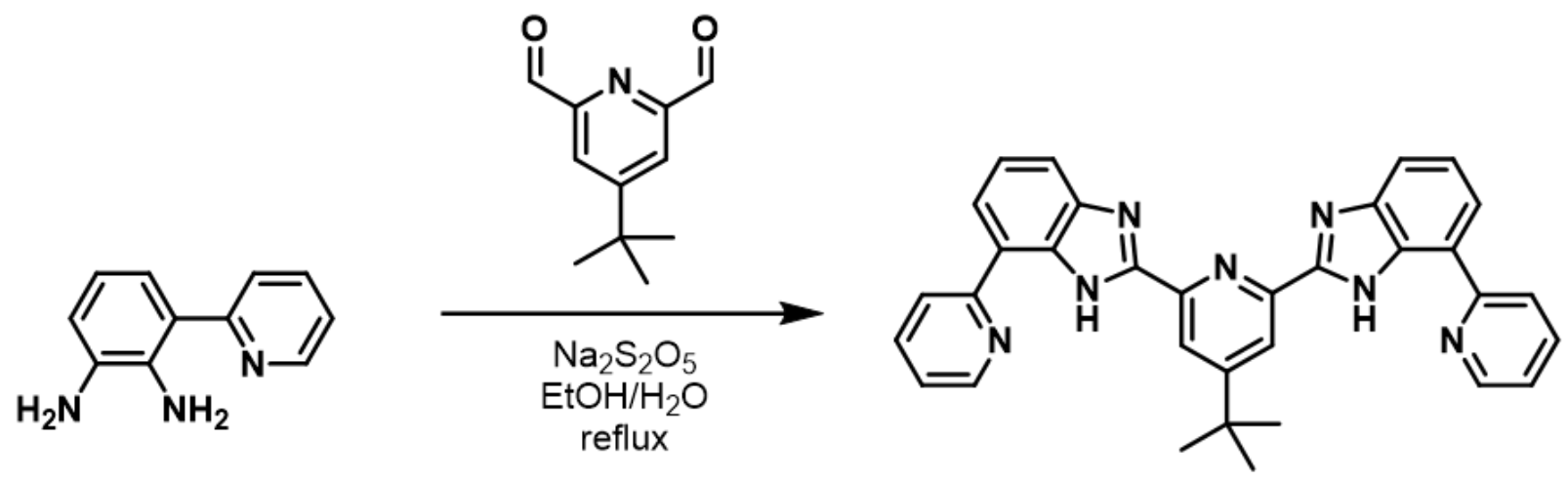

4b **5b**

2,2′-(4-(*tert*-Butyl)pyridine-2,6-diyl)bis(7-(pyridin-2-yl)-1*H*-benzo[*d*]imidazole) (5b). A 100 mL round-bottom flask was charged with **4b** (0.432 g, 2.33 mmol) and 4-(*tert*-butyl)pyridine-2,6-dicarbaldehyde (0.220 g, 1.15 mmol). A portion of anhyd EtOH (35 mL) was added, and the mixture was stirred at 50 °C. To the solution was added an aqueous solution (2 mL) of $Na_2S_2O_5$ (0.290 g, 1.53 mmol). The reaction mixture was heated at reflux for 24 h under Ar atmosphere. After cooling to r.t., volatile fractions were removed under reduced pressure, and the residual material was diluted with EtOAc (30 mL) and H_2O (50 mL). The organic phase was washed with brine (30 mL × 3), dried over anhyd $MgSO_4$, filtered, and concentrated under reduced pressure. Flash column chromatography on SiO_2 (hexane:EtOAc = 100:0 to 50:50, v/v) furnished **5b** as a beige solid (0.489 g, 0.937 mmol, yield = 82%). ^{1}H NMR (500 MHz, $CDCl_3$, 298 K) δ 12.67 (s, 2H), 8.57 – 8.55 (m, 2H), 8.53 (s, 2H), 8.03 (d, J = 8.2 Hz, 2H), 7.97 (d, J = 8.0 Hz, 2H), 7.86 (d, J = 7.4 Hz, 2H), 7.79 (td, J = 7.9, 1.8 Hz, 2H), 7.41 (t, J = 7.8 Hz, 2H), 7.09 (ddd, J = 7.4, 4.9, 0.8 Hz, 2H), 1.52 (s, 9H). ^{13}C NMR (125 MHz, $CDCl_3$, 298 K) δ 163.02, 156.61, 151.48, 149.50, 148.31, 145.66, 136.90, 133.21, 122.57, 122.24, 121.53, 121.43, 120.67, 119.84, 119.70, 35.57, 30.67. FT-IR (ATR, cm^{-1}): 3405, 3057, 2965, 2208, 1606, 1589, 1562, 1467, 1447, 1435, 1313, 1283, 1199, 1056, 908, 770, 725. HRMS (ESI) calcd. for $C_{33}H_{28}N_9$ [M + H]$^+$ 522.2401, found 522.2399.

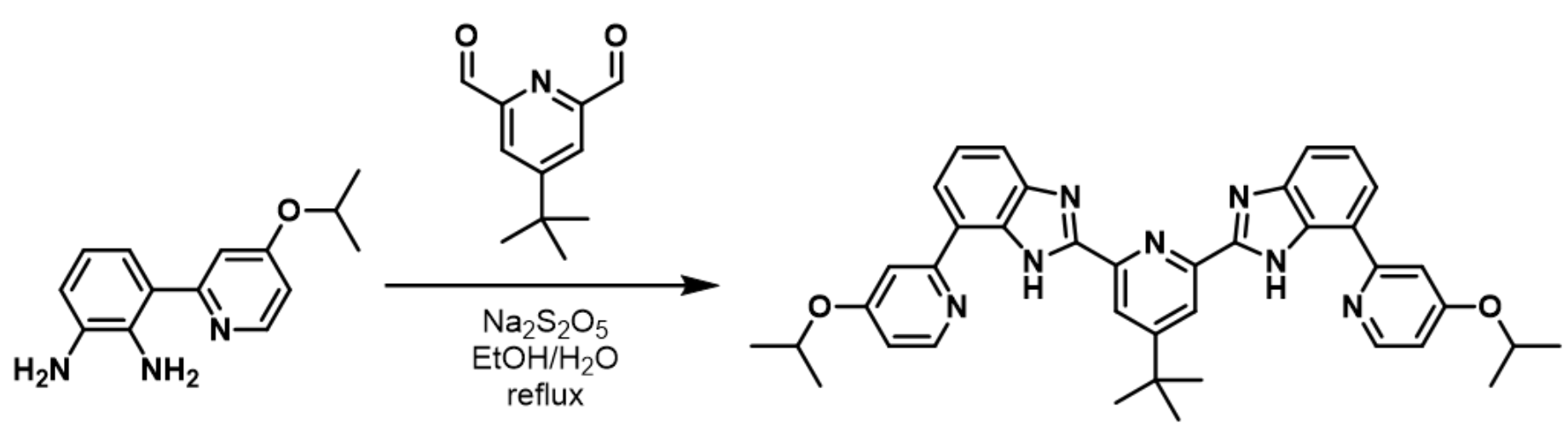

4c **5c**

2,2′-(4-(*tert*-Butyl)pyridine-2,6-diyl)bis(7-(4-isopropoxypyridin-2-yl)-1*H*-benzo[*d*]imidazole) (5c). A 100 mL round-bottom flask was charged with **4c** (0.450 g, 1.85 mmol) and 4-(*tert*-butyl)pyridine-2,6-dicarbaldehyde (0.167 g, 0.873 mmol). A portion of anhyd EtOH (20 mL) was added, and the mixture was stirred at 50 °C. To the solution was added an aqueous solution (2 mL) of $Na_2S_2O_5$ (0.220 g, 1.16 mmol). The reaction mixture was heated at reflux for 24 h under an Ar atmosphere. After cooling to r.t., volatile fractions were removed under reduced pressure, and the residual material was diluted with CH_2Cl_2 (50 mL) and H_2O (50 mL). The organic phase was washed with brine (50 mL × 3), dried over anhyd $MgSO_4$, filtered, and concentrated under reduced pressure. Recrystallization from EtOH furnished **5c** as

an off-white solid (0.330 g, 0.517 mmol, yield = 45%). ^{1}H NMR (500 MHz, $CDCl_3$, 298 K) δ 12.76 (br, 2H), 8.54 (s, 2H), 8.45 (d, J = 5.7 Hz, 2H), 7.96 (d, J = 8.0 Hz, 2H), 7.83 (d, J = 7.6 Hz, 2H), 7.54 (d, J = 1.8 Hz, 2H), 7.41 (t, J = 7.8 Hz, 2H), 6.68 (dd, J = 5.7, 2.0 Hz, 2H), 4.78 (m, J = 6.0 Hz, 2H), 1.51 (s, 9H), 1.46 (d, J = 6.1 Hz, 12H). ^{13}C NMR (125 MHz, $CDCl_3$, 298 K) δ 164.89, 162.95, 158.29, 151.48, 150.92, 148.34, 145.59, 133.46, 122.48, 122.44, 121.28, 120.45, 119.84, 109.28, 106.75, 70.16, 35.54, 30.65, 21.88. FT-IR (ATR, cm^{-1}): 3397, 2971, 1590, 1557, 1471, 1447, 1309, 1227, 1110, 1008, 994, 909, 887, 876, 800, 729. HRMS (ESI) calcd. for $C_{39}H_{40}N_7O_2$ $[M + H]^+$ 638.3238, found 638.3241.

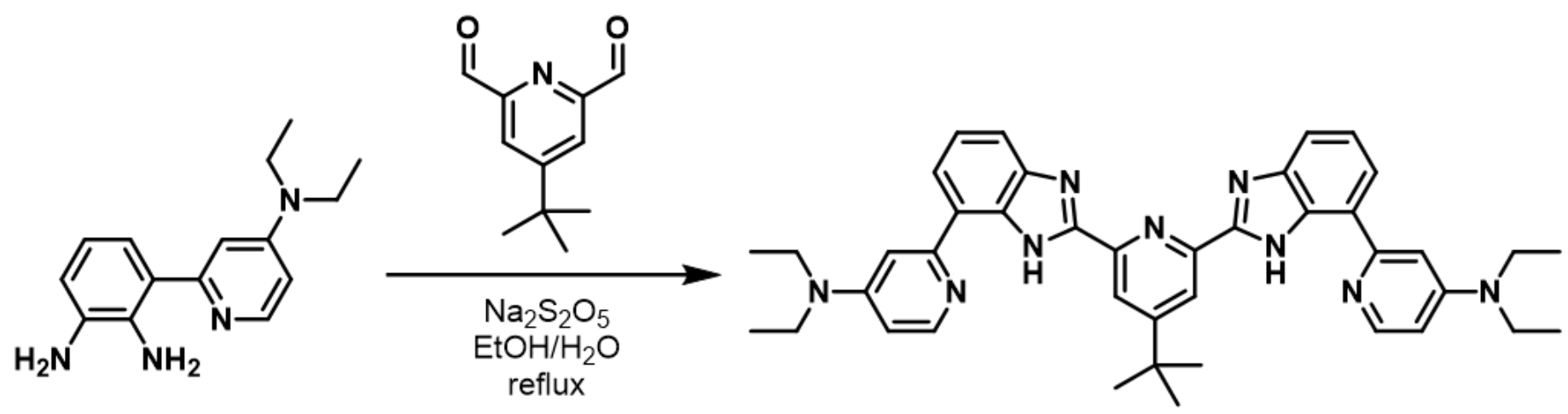

4d

5d

2,2′-((4-(*tert*-Butyl)pyridine-2,6-diyl)bis(1*H*-benzo[*d*]imidazole-2,7-diyl))bis(*N*,*N*-diethylpyridin-4-amine) (5d). A 500 mL round-bottom flask was charged with **4d** (2.86 g, 11.1 mmol) and 4-(*tert*-butyl)pyridine-2,6-dicarbaldehyde (1.00 g, 5.27 mmol). A portion of anhyd EtOH (65 mL) was added, and the mixture was stirred at 50 °C. To the solution was added an aqueous solution (4 mL) of $Na_2S_2O_5$ (1.30 g, 6.85 mmol). The reaction mixture was heated at reflux for 46 h under Ar atmosphere. After cooling to r.t., volatile fractions were removed under reduced pressure, and the residual material was diluted with EtOAc (70 mL) and H_2O (70 mL). The organic phase was washed with brine (50 mL × 3), dried over anhyd $MgSO_4$, filtered, and concentrated under reduced pressure. Flash column chromatography on SiO_2 (hexane:acetone = 100:0 to 60:40, v/v) furnished **5d** as a beige solid (0.466 g, 0.702 mmol, yield = 15%). ^{1}H NMR (500 MHz, $CDCl_3$, 298 K) δ 12.86 (s, 2H), 8.54 (s, 2H), 8.32 (d, J = 5.9 Hz, 2H), 7.93 (d, J = 7.9 Hz, 2H), 7.82 (d, J = 7.5 Hz, 2H), 7.40 (t, J = 7.8 Hz, 2H), 7.23 (d, J = 2.0 Hz, 2H), 6.42 (dd, J = 5.9, 2.3 Hz, 2H), 3.50 (q, J = 7.1 Hz, 8H), 1.51 (s, 9H), 1.30 (t, J = 7.1 Hz, 12H). ^{13}C NMR (125 MHz, $CDCl_3$, 298 K) δ 162.75, 156.88, 152.74, 151.40, 150.20, 148.44, 145.49, 133.69, 123.66, 122.27, 120.55, 120.06, 119.69, 104.87, 101.93, 44.05, 35.51, 30.65, 12.52. FT-IR (ATR, cm^{-1}): 3327, 2969, 2930, 2870, 1595, 1560, 1536, 1497, 1448, 1420, 1355, 1290, 1256, 1077, 1027, 986, 853. HRMS (ESI) calcd. for $C_{41}H_{47}N_9$ $[M + 2H]^{2+}$ 332.6972, found 332.6974.

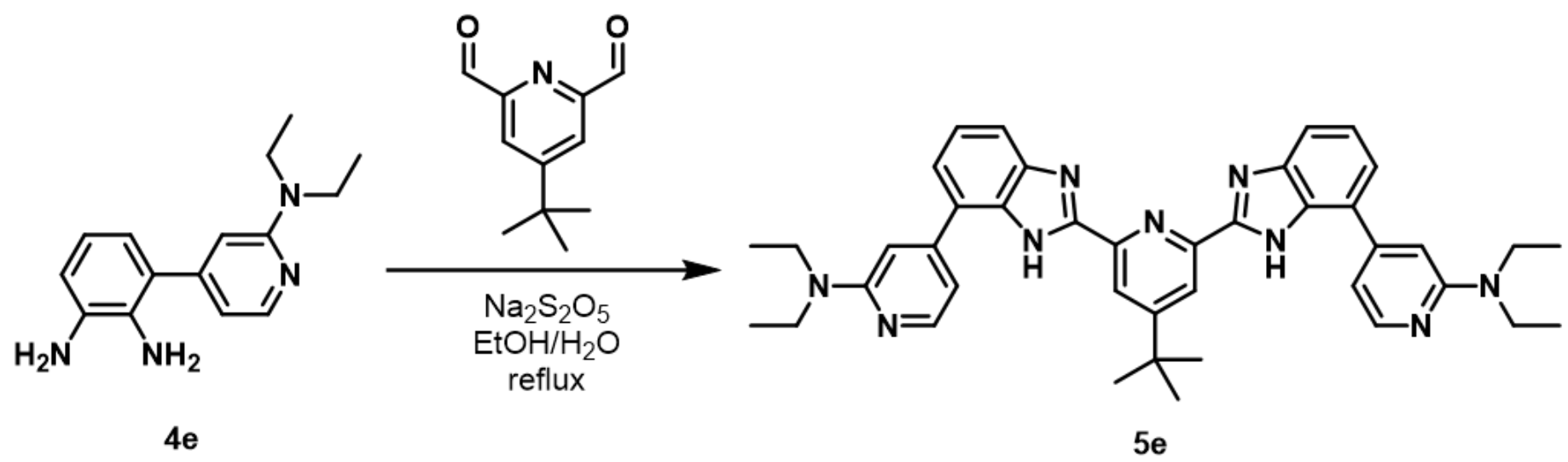

4,4′-((4-(*tert*-Butyl)pyridine-2,6-diyl)bis(1*H*-benzo[*d*]imidazole-2,7-diyl))bis(*N*,*N*-diethylpyridin-2-amine) (5e). A 500 mL round-bottom flask was charged with **4e** (1.66 g, 6.41 mmol) and 4-(*tert*-butyl)pyridine-2,6-dicarbaldehyde (0.600 g, 3.13 mmol). A portion of anhyd EtOH (90 mL) was added, and the mixture was stirred at 50 °C. To the solution was added an aqueous solution (2 mL) of $Na_2S_2O_5$ (0.780 g, 4.06 mmol). The reaction mixture was heated at reflux for 29 h under Ar atmosphere. After cooling to r.t., volatile fractions were removed under reduced pressure, and the residual material was diluted with EtOAc (200 mL) and H_2O (200 mL). The organic phase was washed with brine (200 mL × 3), dried over anhyd $MgSO_4$, filtered, and concentrated under reduced pressure. Flash column chromatography on SiO_2 (hexane:EtOAc = 100:0 to 80:20, v/v) furnished **5e** as a yellow solid (1.94 g, 2.92 mmol, yield = 92%). ^{1}H NMR (500 MHz, THF-d_8, 298 K) δ 12.31 (s, 2H), 8.53 (s, 2H), 8.16 (d, J = 5.1 Hz, 2H), 7.86 (s, 2H), 7.56 (d, J = 8.4 Hz, 2H), 7.53 (d, J = 8.4 Hz, 2H), 7.37–7.33 (m, 2H), 7.01 (d, J = 5.2 Hz, 2H), 3.70–3.66 (q, J = 7.0 Hz, 8H), 1.47 (s, 9H), 1.33 (t, J = 7.0 Hz, 12H). ^{13}C NMR (125 MHz, THF-d_8, 298 K) δ 162.72, 157.87, 151.05, 148.57, 147.84, 146.32, 142.70, 135.55, 131.12, 123.56, 120.89, 118.61, 110.98, 110.21, 106.74, 42.51, 35.11, 29.88, 12.91. FT-IR (ATR, cm^{-1}): 3182, 2966, 2929, 1593, 1537, 1493, 1477, 1446, 1358, 1281, 1257, 1242, 1202, 1033, 881, 850, 790, 749. HRMS (ESI) calcd. for $C_{41}H_{46}N_9$ [M + H]$^+$ 664.3871, found 664.3768.

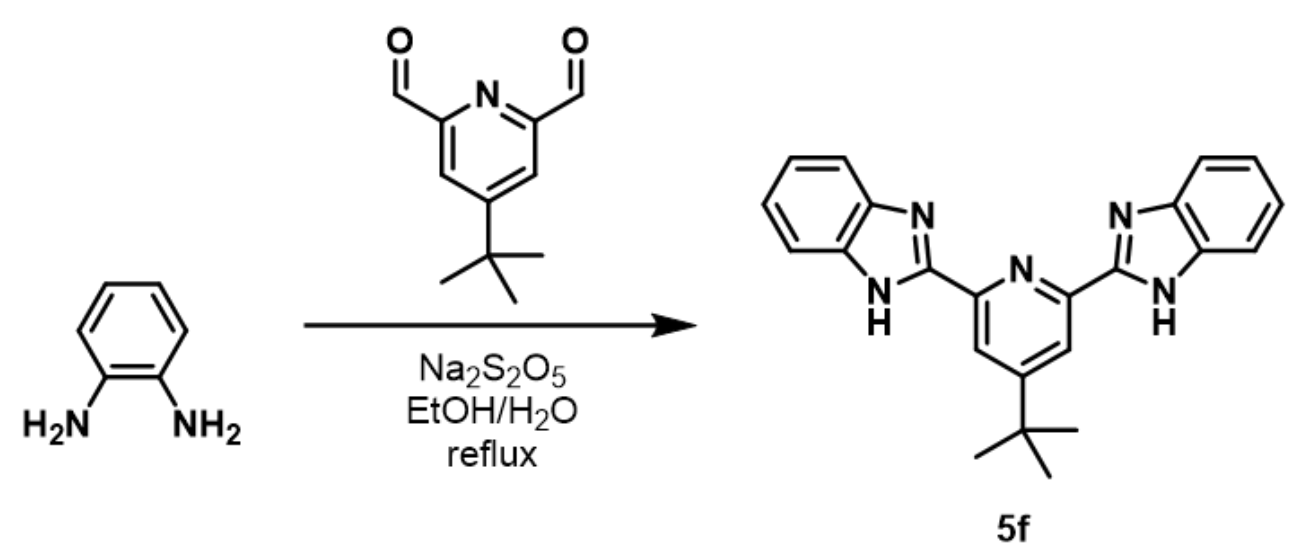

2,2'-(4-(*tert*-Butyl)pyridine-2,6-diyl)bis(1*H*-benzo[*d*]imidazole) (5f). A 100 mL round-bottom flask was charged with benzene-1,2-diamine (0.149 g, 1.38 mmol), 4-(*tert*-butyl)pyridine-2,6-dicarbaldehyde (0.304 g, 0.763 mmol), and anhyd EtOH (10 mL). The mixture was stirred at reflux under air for 24 h. After cooling to r.t., water (60 mL) was added to induce precipitation. The mixture was filtered through a sintered glass filter, and washed with excess amount of water. The isolated solid was recrystallized from EtOH to furnish **5f** as a beige solid (0.136 g, 0.248 mmol, yield = 36%). ^{1}H NMR (500 MHz, DMSO-d_6, 298 K) δ 12.98 (s, 2H), 8.35 (s, 2H), 7.80 (d, J = 8.0 Hz, 2H), 7.74 (d, J = 8.0 Hz, 2H), 7.36 (t, J = 7.5 Hz, 2H), 7.29 (t, J = 7.6 Hz, 2H), 1.45 (s, 9H). ^{13}C NMR (125 MHz, $CDCl_3$, 298 K) δ 162.70, 150.70, 147.88, 144.12, 134.37, 123.67, 122.20, 119.65, 118.13, 111.77, 35.09, 30.17. FT-IR (ATR, cm^{-1}): 3218, 2963, 1607, 1562, 1439, 1392, 1324, 1273, 1222, 1147, 1133, 1112, 1014, 997, 893, 875, 851, 767, 746, 738. HRMS (ESI) calcd. for $C_{23}H_{22}N_5$ [M + H]$^+$ 368.1870, found 368.1870.

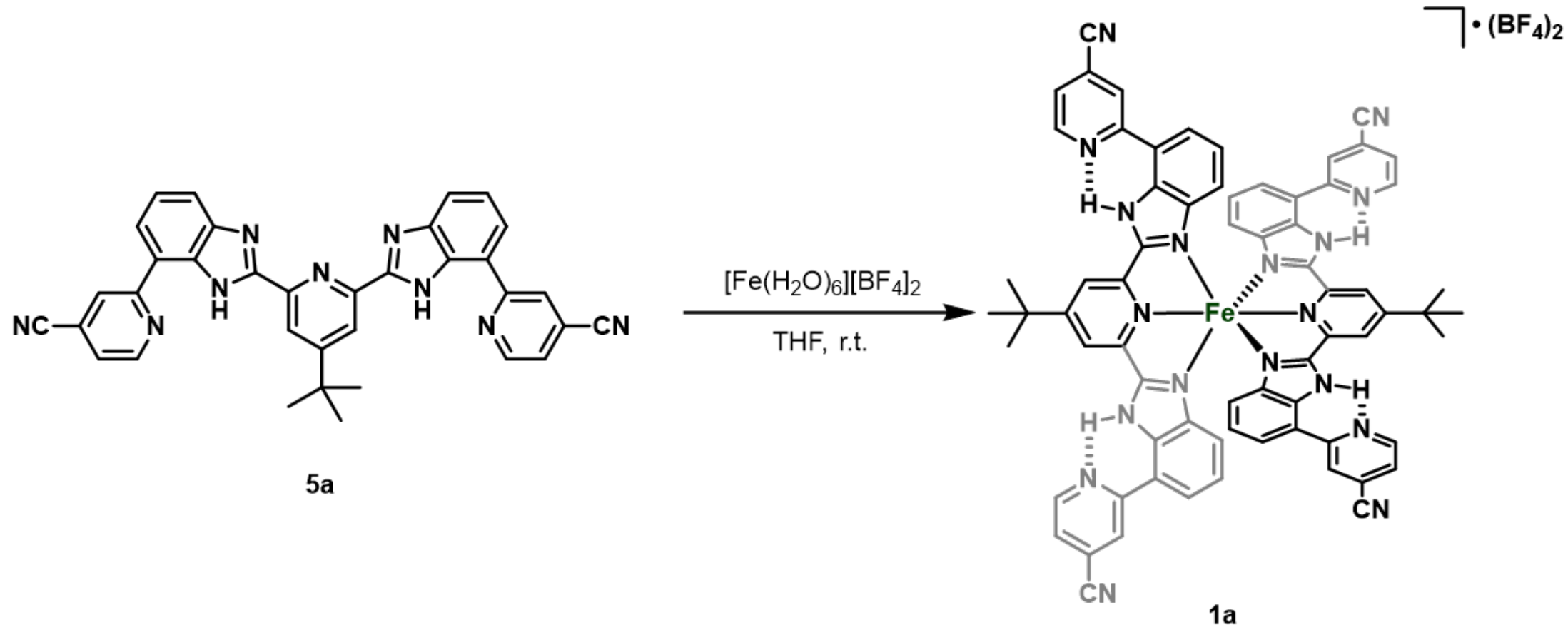

$[Fe(\mathbf{5a})_2](BF_4)_2$ (1a). A mixture of **5a** (250 mg, 0.437 mmol), $Fe(BF_4)_2{\cdot}6H_2O$ (91.0 mg, 0.270 mmol), and THF (14 mL) was stirred at r.t. for 24 h under Ar. To the mixture was added Et_2O (10 mL) and MTBE (10 mL) to induce precipitation of a violet solid, which was isolated by filtration to afford **1a**·MTBE·H_2O (248 mg, 0.168 mmol, yield = 77%). Anal. Calcd. for $FeC_{75}H_{64}B_2F_8N_{18}O_2$ (**1a**·MTBE·H_2O): C, 60.91; H, 4.36; N, 17.05. Found: C, 61.15; H, 4.69; N, 16.83.

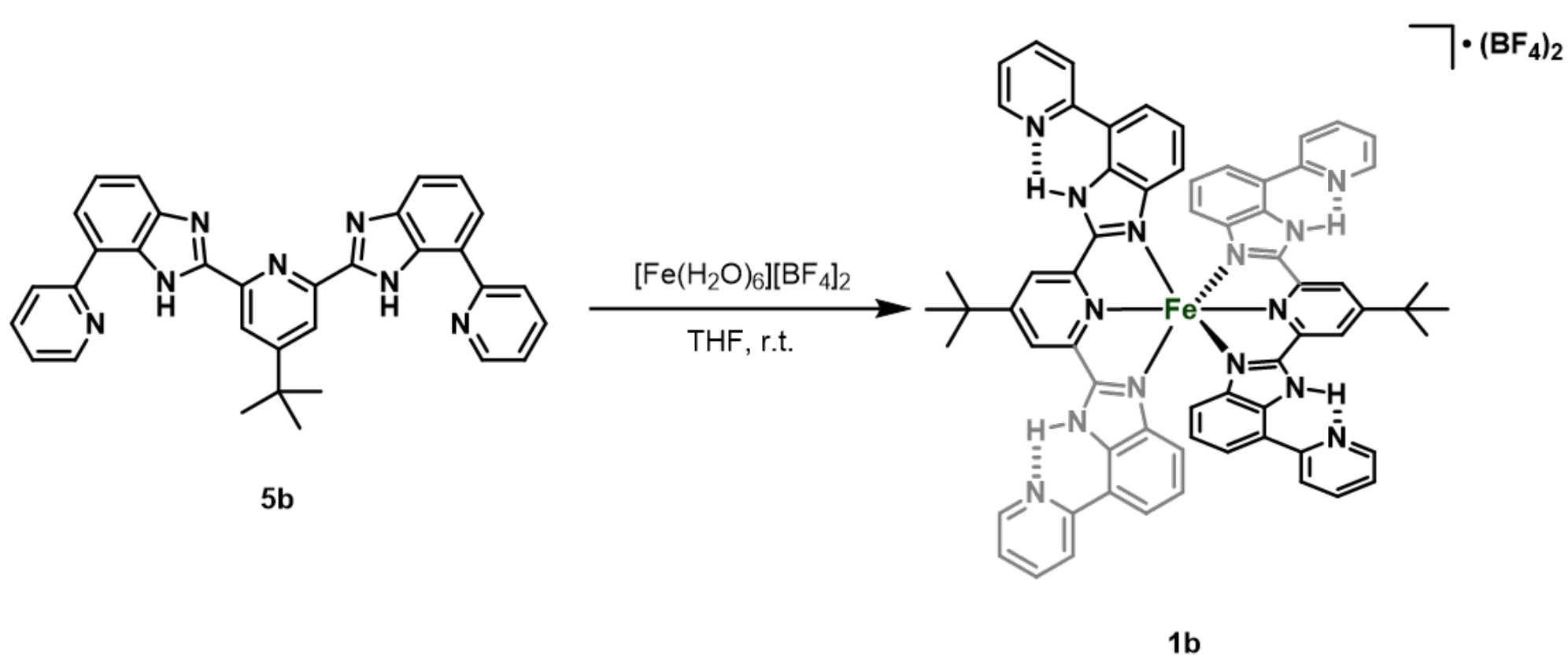

$[Fe(\mathbf{5b})_2](BF_4)_2$ (1b). A mixture of **5b** (100 mg, 0.192 mmol), $Fe(BF_4)_2{\cdot}6H_2O$ (32.3 mg, 95.7 μmol), and THF (5 mL) was stirred at r.t. for 24 h under Ar. To the mixture was added Et_2O (2 mL) and MTBE (2 mL) to induce precipitation of a violet solid, which was isolated by filtration to afford **1b**·3.5THF·3H_2O (50.0 mg, 31.7 μmol, yield = 33%). Anal. Calcd. for $FeC_{80}H_{88}B_2F_8N_{14}O_{6.5}$ (**1b**·3.5THF·3H_2O): C, 60.85; H, 5.62; N, 12.42. Found: C, 60.84; H, 5.27; N, 12.12.

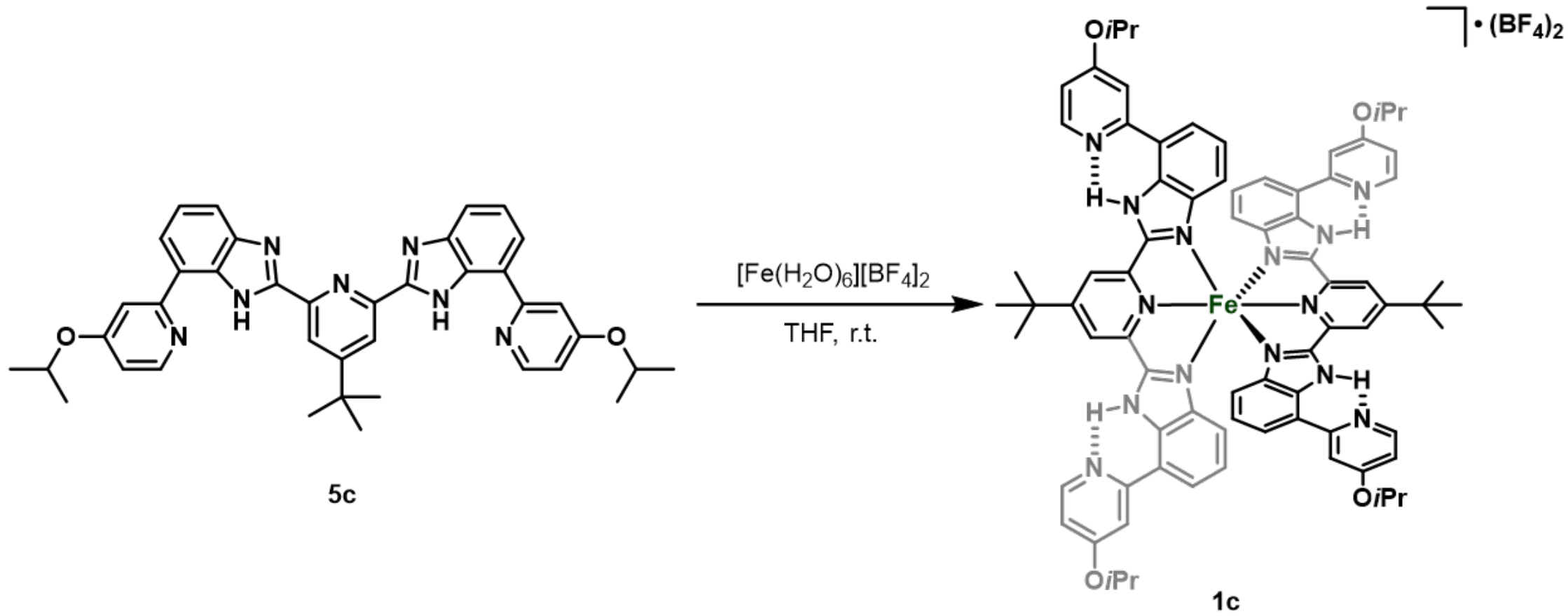

$[Fe(\mathbf{5c})_2](BF_4)_2$ (1c). A mixture of **5c** (109 mg, 0.171 mmol), $Fe(BF_4)_2{\cdot}6H_2O$ (28.8 mg, 85.3 μmol), and THF (4 mL) was stirred at r.t. for 24 h under Ar. To the mixture was added Et_2O (2 mL) and MTBE (2 mL) to induce precipitation of a dark blue solid, which was isolated by filtration to afford **1c**$\cdot$0.5THF$\cdot$2H_2O (98.0 mg, 0.0621 mmol, yield = 73%). Anal. Calcd. for $FeC_{78}H_{86}B_2F_8N_{14}O_8$ (**1c**$\cdot$0.5THF$\cdot$2H_2O): C, 59.40; H, 5.50; N, 12.43. Found: C, 59.01; H, 5.71; N, 12.77.

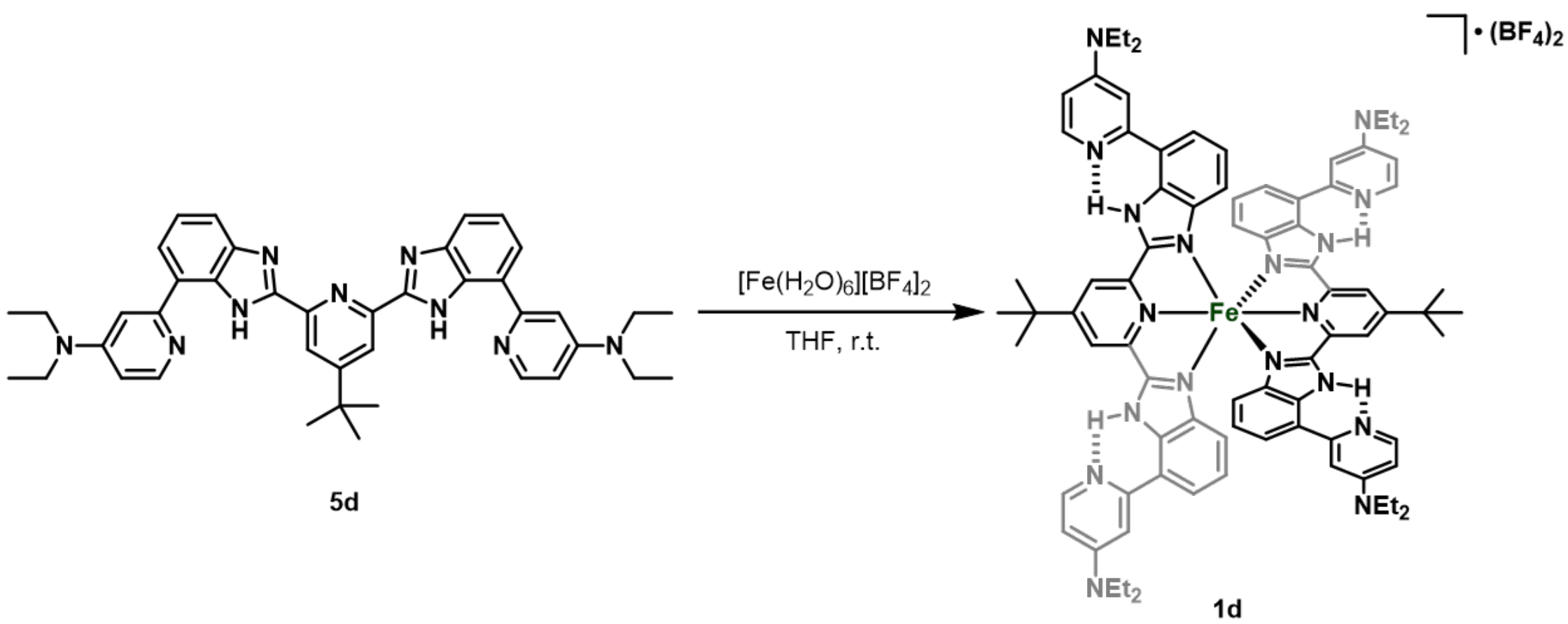

$[Fe(\mathbf{5d})_2](BF_4)_2$ (1d). A mixture of **5d** (208 mg, 0.313 mmol), $Fe(BF_4)_2{\cdot}6H_2O$ (52.8 mg, 0.156 mmol), and THF (6 mL) was stirred at r.t. for 24 h under Ar. To the mixture was added Et_2O (5 mL) and MTBE (5 mL) to induce precipitation of a dark blue solid, which was isolated by filtration to afford **1d**$\cdot$0.5THF$\cdot$2H_2O (195 mg, 0.120 mmol, yield = 77%). Anal. Calcd. for $FeC_{84}H_{98}B_2F_8N_{18}O_{2.5}$ (**1d**$\cdot$0.5THF$\cdot$2H_2O): C, 61.92; H, 6.06; N, 15.47. Found: C, 62.00; H, 5.68; N, 15.16.

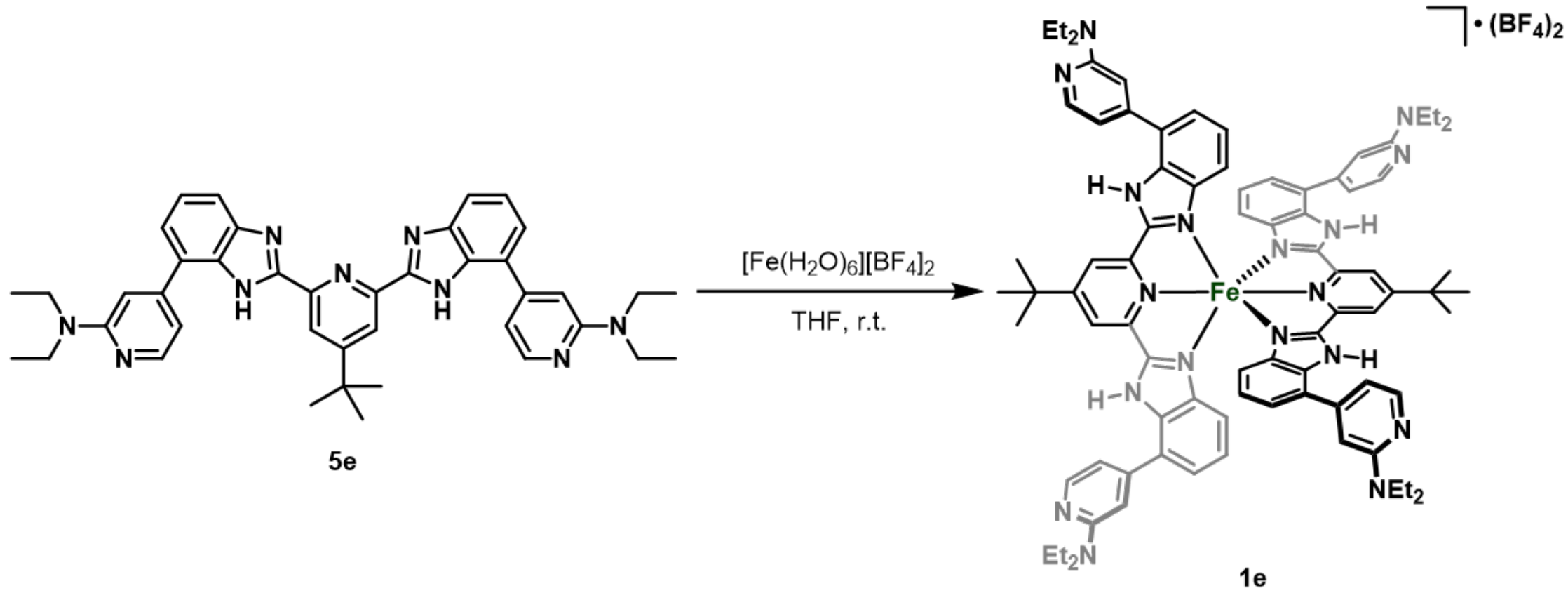

$[Fe(\mathbf{5e})_2](BF_4)_2$ (1e). A mixture of **5e** (100 mg, 0.151 mmol), $Fe(BF_4)_2{\cdot}6H_2O$ (25.5 mg, 75.5 μmol), and THF (3 mL) was stirred at r.t. for 24 h under Ar. To the mixture was added Et_2O (2 mL) to induce precipitation of a violet solid, which was isolated by filtration to afford **1e**$\cdot 3.5H_2O$ (105 mg, 64.8 μmol, yield = 86%). Anal. Calcd. for $FeC_{82}H_{97}B_2F_8N_{18}O_{3.5}$ (**1e**$\cdot 3.5H_2O$): C, 60.79; H, 6.03; N, 15.56. Found: C, 60.85; H, 5.80; N, 15.17.

To obtain better diffraction-quality single crystals, the anion-substituted variant of **1e** was prepared by stirring a mixture of **5e** (30 mg, 0.0452 mmol), $[Fe(MeCN)_6](BAr^F_4)_2$ (45.8 mg, 0.0226 mmol), and THF (3 mL) at r.t. for 24 h under Ar. The volatile fractions were removed under reduced pressure, and the resulting residue was dissolved in a minimum amount of $MeCN/CH_2Cl_2$ (1:1, v/v). Vapor diffusion of cyclohexane produced violet crystals of **1e-BAr**$^F_4$ (34 mg, 0.0109 mmol, yield = 48%).

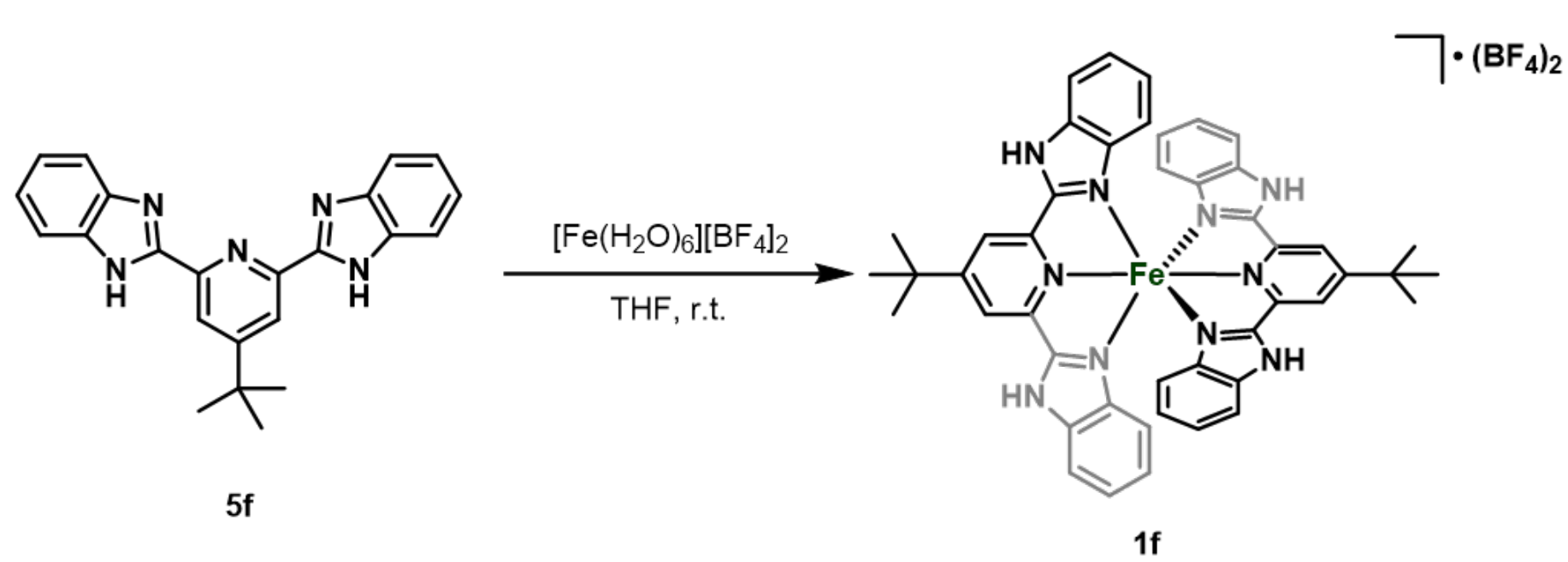

$[Fe(\mathbf{5f})_2](BF_4)_2$ (1f). A mixture of **5f** (108 mg, 0.294 mmol), $Fe(BF_4)_2{\cdot}6H_2O$ (50.1 mg, 0.148 mmol), and THF (4 mL) was stirred at r.t. for 24 h under air. A blue precipitate was isolated by filtration to afford **1f**$\cdot$THF$\cdot 2H_2O$ (112 mg, 0.104 mmol, yield = 70%). Anal. Calcd. for $FeC_{50}H_{54}B_2F_8N_{10}O_3$ (**1f**$\cdot$THF$\cdot 2H_2O$): C, 56.00; H, 5.08; N, 13.06. Found: C, 56.12; H, 5.09; N, 13.30.

3. Single-Crystal X-ray Diffraction Studies

X-ray Crystallographic Studies on 5d. Single crystals of **5d** were prepared by recrystallization of this material from hot EtOH. A colorless crystal (approximate dimensions 0.085 × 0.055 × 0.030 mm^3) was coated with Parabar 10312 (Hampton Research Inc.) to mount on the micro-loop under a cold nitrogen stream at $T = 100$ K. The diffraction data were measured using synchrotron radiation ($\lambda = 0.700$ Å) employing a PLSII-2D SMC on a Rayonix MX225HS CCD area detector with high precision one-axis goniostat at Pohang Accelerator Laboratory, Korea. The PAL BL2D-SMC program[4] was used for data collection, and HKL3000sm (Ver.717)[5] was used for cell refinement, reduction, and absorption correction. A total of 42953 reflections were measured (3.686° $\leq 2\theta \leq$ 67.312°). The structure was solved with SHELXT[6] using direct methods, and refined with SHELXL[7] refinement package of OLEX2.[8] A total of 12375 unique reflections were used in all calculations. The final $R1$ was 0.0620 ($I \geq 2\sigma(I)$) and $wR2$ was 0.1763 (all data). CCDC 2547896 contains the supplementary crystallographic data for this structure.

X-ray Crystallographic Studies on 5e. Single crystals of **5e** were prepared by recrystallization of this material from hot EtOH. A colorless crystal (approximate dimensions 0.205 × 0.145 × 0.105 mm^3) was placed onto a nylon loop with Paratone-N oil, and mounted on an XtaLAB AFC12 (RINC): Kappa dual home/near diffractometer. The data collection was carried out using Cu Kα radiation and the crystal was kept at $T = 93$ K. A total of 26364 reflections were measured (6.986° $\leq 2\theta \leq$ 158.542°). The structure was solved with SHELXT[6] using direct methods, and refined with SHELXL[7] refinement package of OLEX2.[8] A total of 7832 unique reflections were used in all calculations. The final $R1$ was 0.0394 ($I \geq 2\sigma(I)$) and $wR2$ was 0.1165 (all data). CCDC 2547893 contains the supplementary crystallographic data for this structure.

X-ray Crystallographic Studies on 1a. Single crystals of **1a** were prepared by slow diffusion of MTBE into a DMF solution of this material. A violet crystal (approximate dimensions 0.402 × 0.286 × 0.165 mm^3) was placed onto a nylon loop with Paratone-N oil, and mounted on an XtaLAB AFC12 (RINC): Kappa dual home/near diffractometer. The data collection was carried out using Cu Kα radiation and the crystal was kept at $T = 193$ K. A total of 258569 reflections were measured (4.754° $\leq 2\theta \leq$ 158.844°). The structure was solved with SHELXT[6] using direct methods, and refined with SHELXL[7] refinement package of OLEX2.[8] A total of 77333 unique reflections were used in all calculations. The final $R1$ was 0.0829 ($I \geq 2\sigma(I)$) and $wR2$ was 0.2761 (all data). CCDC 2547895 contains the supplementary crystallographic data for this structure.

X-ray Crystallographic Studies on 1e-BAr$^F_4$. Single crystals of **1e-BAr$^F_4$** were prepared by slow diffusion of cyclohexane into a CH_2Cl_2:MeCN = 1:1 solution of this material. A violet crystal (approximate dimensions 0.05 × 0.05 × 0.03 mm^3) was coated with Parabar 10312 (Hampton Research Inc.) to mount on the micro-loop under a cold nitrogen stream at $T = 100$ K. The diffraction data were measured using synchrotron radiation ($\lambda = 0.700$ Å) employing a PLSII-11C Micro-MX beamline on a Pilatus 3 6M area detector with a high-precision one-axis goniostat at the Pohang Accelerator Laboratory, Korea. The 11C Micro-MX data collection program[9] was used for

[4]J. W. Shin, K. Eom and D. Moon, *J. Synchrotron Rad.*, 2016, **23**, 369–373.

[5]Z. Otwinowski and W. Minor, *Methods Enzymol.*, 1997, **276**, 307–326.

[6]G. M. Sheldrick, *Acta Cryst. A*, 2015, **71**, 3–8.

[7]G. M. Sheldrick, *Acta Cryst. C*, 2015, **71**, 3–8.

[8]O. V. Dolomanov, L. J. Bourhis, R. J. Gildea, J. A. K. Howard and H. Puschmann, *J. Appl. Cryst.*, 2009, **42**, 339–341.

[9]D.-H. Gu, C. Eo, S.-A. Hwangbo, S.-C. Ha, J. H. Kim, H. Kim, C.-S. Lee, I. D. Seo, Y. D. Yun, W. Lee, H. Choi, J. Kim, J. Lim, S. Rah, J.-S. Kim, J.-O. Lee, Y.-G. Kim and S.-Y. Park, *J. Synchrotron Rad.*, 2021, **28**, 1210–1215.

data collection, and HKL3000 (ver. 717.6)[5] was used for cell refinement, reduction, and absorption correction. A total of 70124 reflections were measured ($1.966^\circ \leq 2\theta \leq 49.628^\circ$). The structure was solved with SHELXT[6] using direct methods, and refined with SHELXL[7] refinement package of OLEX2.[8] A total of 10168 unique reflections were used in all calculations. The final $R1$ was 0.1082 ($I \geq 2\sigma(I)$) and $wR2$ was 0.3548 (all data). CCDC 2547894 contains the supplementary crystallographic data for this structure.

4. Supporting Tables

Table S1. Summary of X-ray crystallographic data for **5d** and **5e**.

	**5d**	**5e**
CCDC number	2547896	2547893
Chemical formula	$C_{41}H_{45}N_9$	$C_{41}H_{45}N_9$
Formula weight	663.86	663.86
Temperature (K)	100	93
Crystal system	monoclinic	monoclinic
Space group	$P2_1/n$	$P2_1/c$
Color of crystal	Colorless	Colorless
a (Å)	11.752(2)	13.11380(10)
b (Å)	20.501(4)	14.56050(10)
c (Å)	15.927(3)	20.04020(10)
α (°)	-	-
β (°)	110.88(3)	105.2320(10)
γ (°)	-	-
Volume (Å^3)	3585.4(14)	3692.12(5)
Z	4	4
R(int)	0.0768	0.0320
Final R indices $[I > 2\sigma(I)]$	$R1 = 0.0620$, $wR2 = 0.1719$	$R1 = 0.0394$, $wR2 = 0.1138$
Final R indices [all data]	$R1 = 0.0671$, $wR2 = 0.1763$	$R1 = 0.0427$, $wR2 = 0.1165$
GOF	1.127	1.082

Table S2. Summary of X-ray crystallographic data for **1a** and **1e-BAr$^F_4$**.

	**1a**	**1e-BAr$^F_4$**
CCDC number	2547895	2547894
Chemical formula	$C_{78}H_{68.75}B_2F_8FeN_{20.25}O_{2.5}$	$C_{146}H_{114}B_2F_{48}FeN_{18}$
Formula weight	1559.25	3110.03
Temperature (K)	193	100
Crystal system	triclinic	monoclinic
Space group	$P\bar{1}$	$I2/a$
Color of crystal	Violet	Violet
a (Å)	25.8624(2)	21.975(4)
b (Å)	26.8839(2)	40.818(8)
c (Å)	28.8831(2)	22.703(5)
α (°)	92.4070(10)	-
β (°)	115.7160(10)	95.94(3)
γ (°)	93.6580(10)	-
Volume (Å^3)	18001.7(3)	20255(7)
Z	8	4
R(int)	0.0580	0.1064
Final R indices $[I > 2\sigma(I)]$	$R1 = 0.0831$, $wR2 = 0.2481$	$R1 = 0.1082$, $wR2 = 0.3297$
Final R indices [all data]	$R1 = 0.1021$, $wR2 = 0.2663$	$R1 = 0.1289$, $wR2 = 0.3548$
GOF	1.081	1.337

Table S3. Selected Fe–N bond lengths (Å) for four crystallographically independent molecules of **1a** in the asymmetric unit. Ligands A and B denote the two tridentate ligands coordinated to each Fe center (py = pyridyl; bzim = benzimidazolyl).

	Molecule 1	Molecule 2	Molecule 3	Molecule 4
$Fe - N(py)_A$	1.900(3)	1.900(3)	1.912(3)	1.905(3)
$Fe - N(bzim)_A$	1.982(2)	1.974(3)	1.993(2)	1.986(2)
$Fe - N(bzim')_A$	1.980(3)	1.981(3)	1.987(2)	1.981(2)
$Fe - N(py)_B$	1.901(3)	1.899(3)	1.901(3)	1.902(3)
$Fe - N(bzim)_B$	1.960(2)	1.964(3)	1.974(3)	1.975(3)
$Fe - N(bzim')_B$	1.972(1)	1.961(2)	1.988(3)	1.978(3)

Table S4. Selected intraligand N···N distances (Å) for the intramolecular N–H···N hydrogen bonds in the four crystallographically independent molecules of **1a** in the asymmetric unit. Ligands A and B denote the two tridentate ligands coordinated to each Fe center. (py = peripheral pyridine; bzim = benzimidazole)

	Molecule 1	Molecule 2	Molecule 3	Molecule 4
$N(py) \cdots N(bzim)_A$	2.779(4)	2.856(4)	2.698(3)	2.711(4)
$N(py) \cdots N(bzim')_A$	2.685(4)	2.754(4)	2.799(4)	2.815(3)
$N(py) \cdots N(bzim)_B$	2.765(4)	2.756(4)	2.789(5)	2.803(4)
$N(py) \cdots N(bzim')_B$	2.805(4)	2.769(4)	2.733(4)	2.745(4)

Table S5. Effective solution-state magnetic moments of **1a–e**. The magnetic moments were calculated with the solvates taken into account.

Complex	Paramagnetic shift (ppm, 500 MHz)	Concentration (mM)	μ_{eff} (μ_B)
**1a**	0.145	6.65	3.52
**1b**	0.077	3.80	3.39
**1c**	0.106	8.24	2.70
**1d**	0.003	2.02	0.92
**1e**	0.093	3.45	3.91

5. Supporting Figures

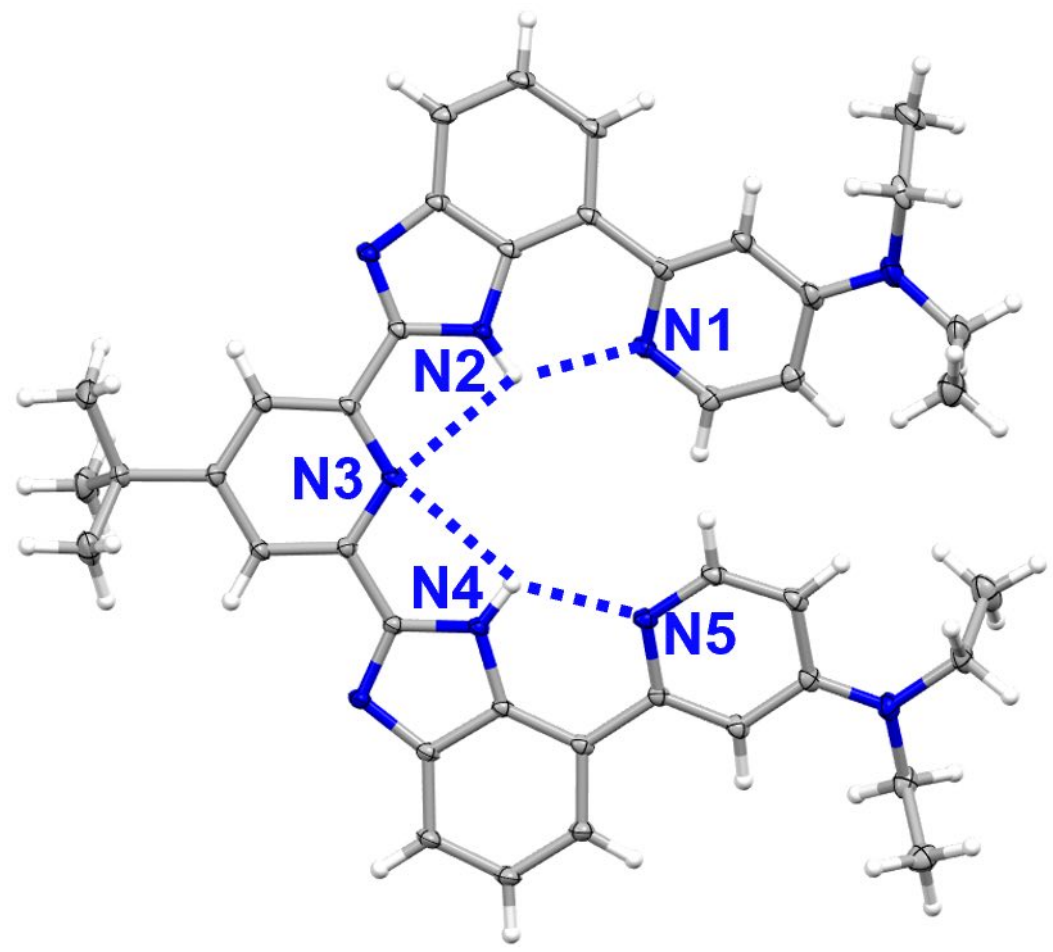

Fig. S1. X-ray structure of **5d** with thermal ellipsoids at the 50% probability level. Selected interatomic distances (Å): N1···N2, 2.745(1); N2···N3, 2.765(1); N3···N4, 2.782(1); N4···N5, 2.727(1).

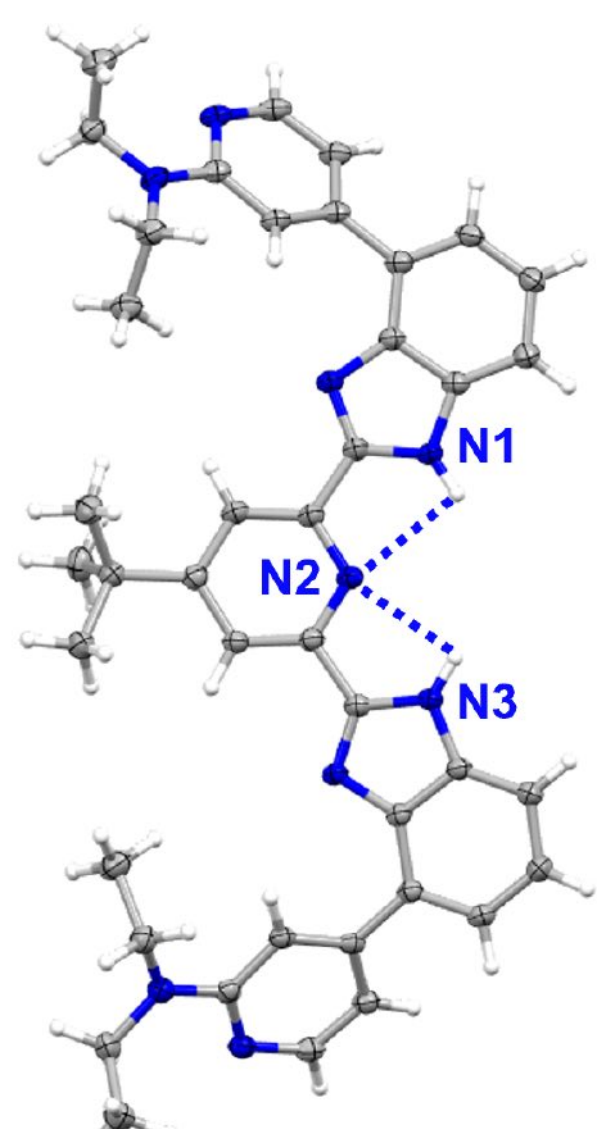

Fig. S2. X-ray structure of **5e** with thermal ellipsoids at the 50% probability level. Selected interatomic distances (Å): N1···N2, 2.722(1); N2···N3, 2.706(1).

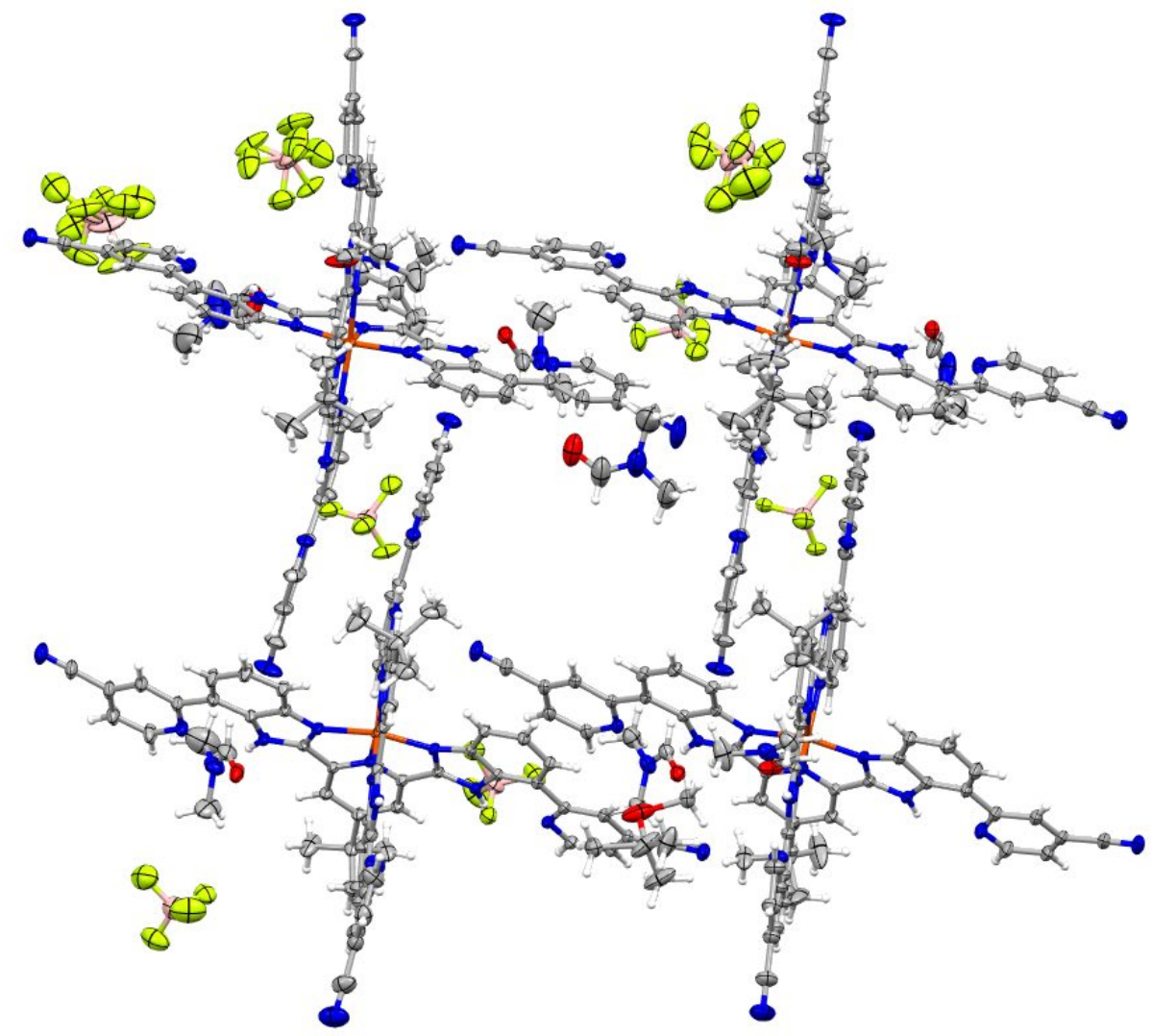

Fig. S3. X-ray crystal structure of **1a** showing the full asymmetric unit, which contains four crystallographically independent molecules of **1a** along with the associated counteranions (BF_4^-) and solvent molecules (DMF and MTBE). Thermal ellipsoids are shown at the 50% probability level.

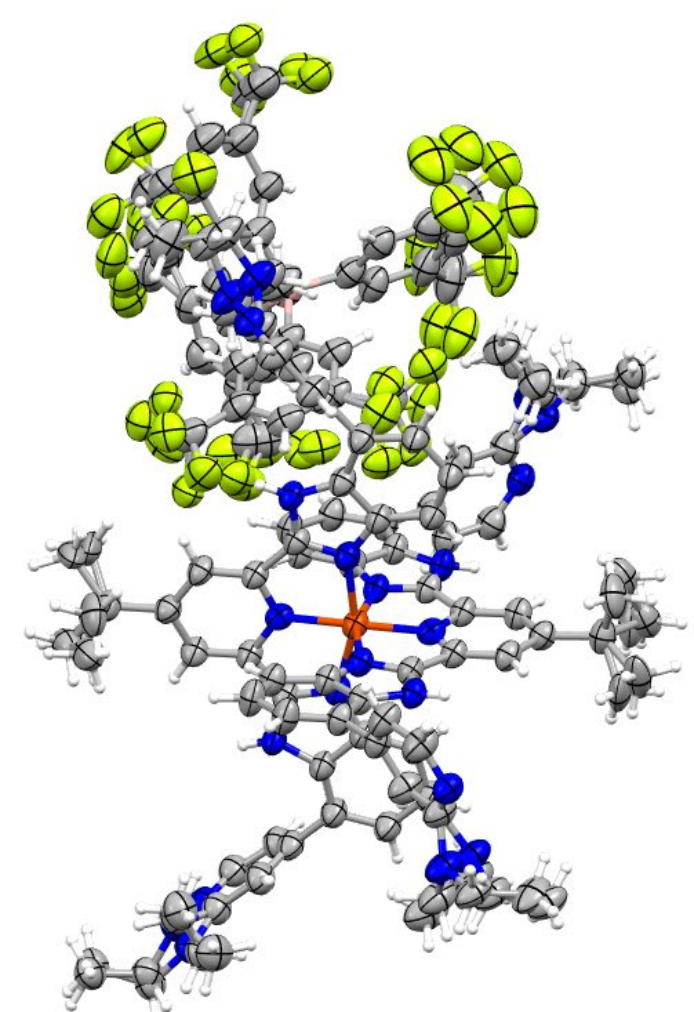

Fig. S4. X-ray crystal structure of **1e-BAr$_4^F$** shown with the associated counteranion (BAr^{F-}). Thermal ellipsoids are shown at the 50% probability level.

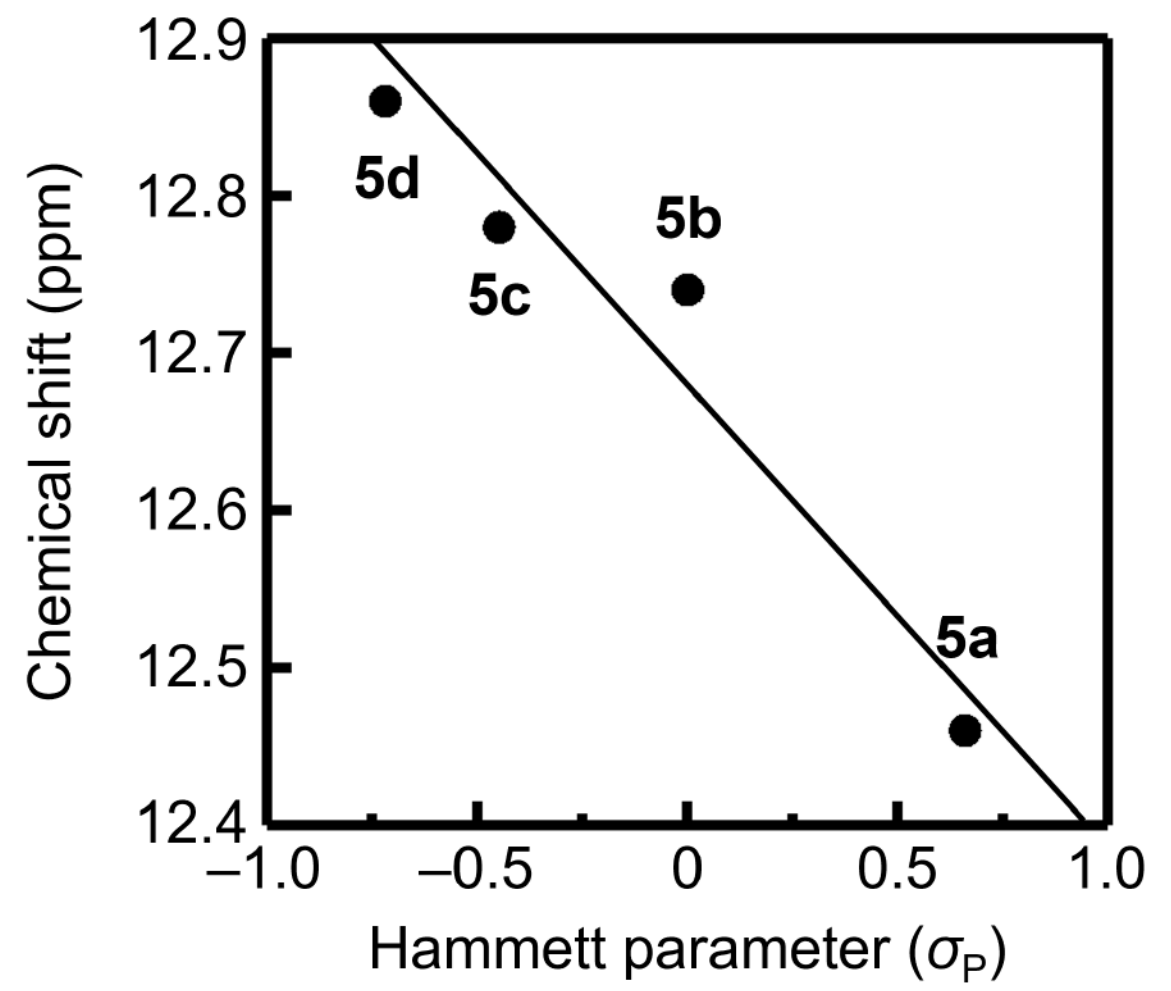

Fig. S5. Correlation between the benzimidazole N–H proton resonances (δ, ppm) of **5a–d** and Hammett parameters (σ_p) of the *para*-substituents on the pendant pyridine rings.

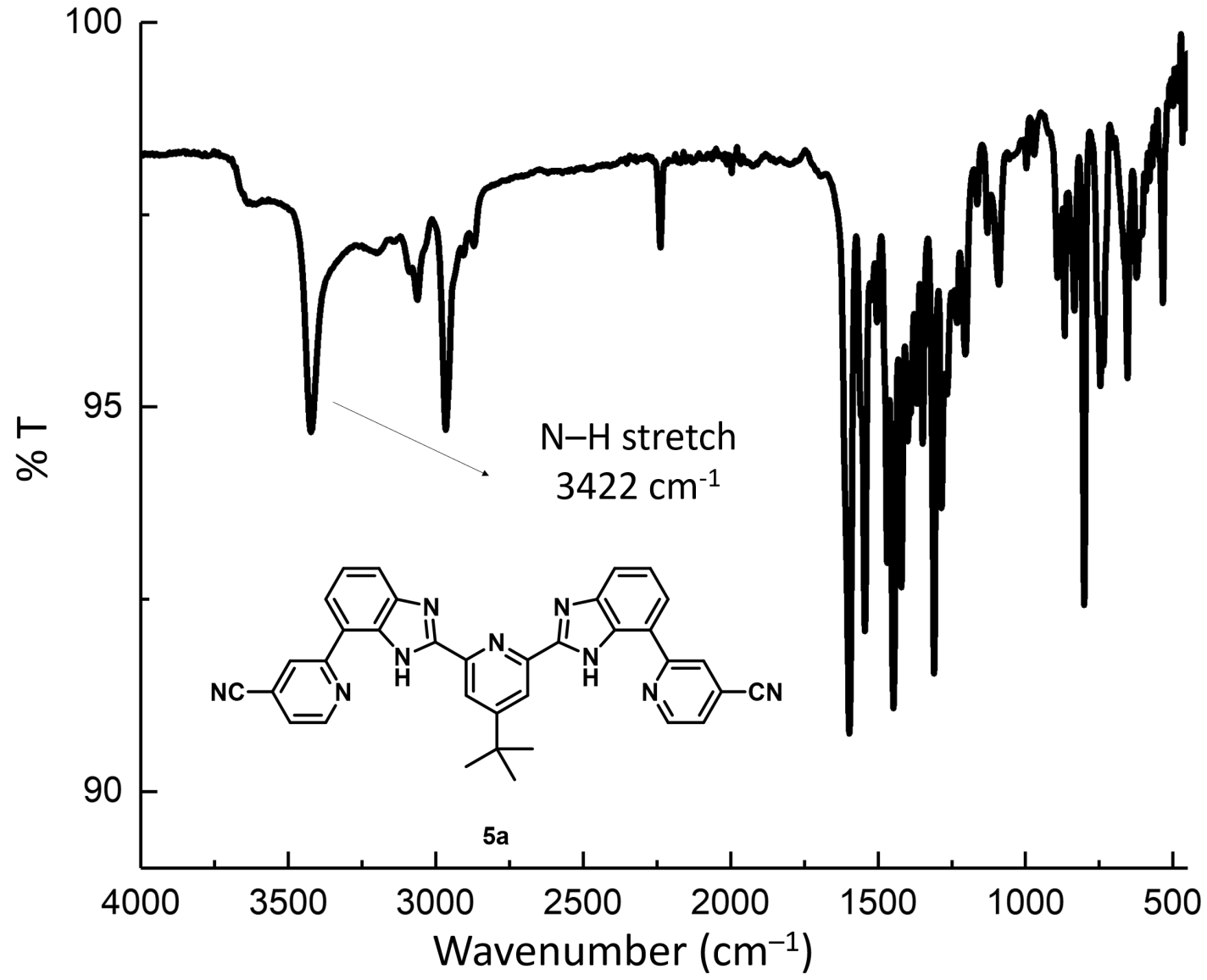

Fig. S6. FT-IR (ATR) spectrum of **5a**.

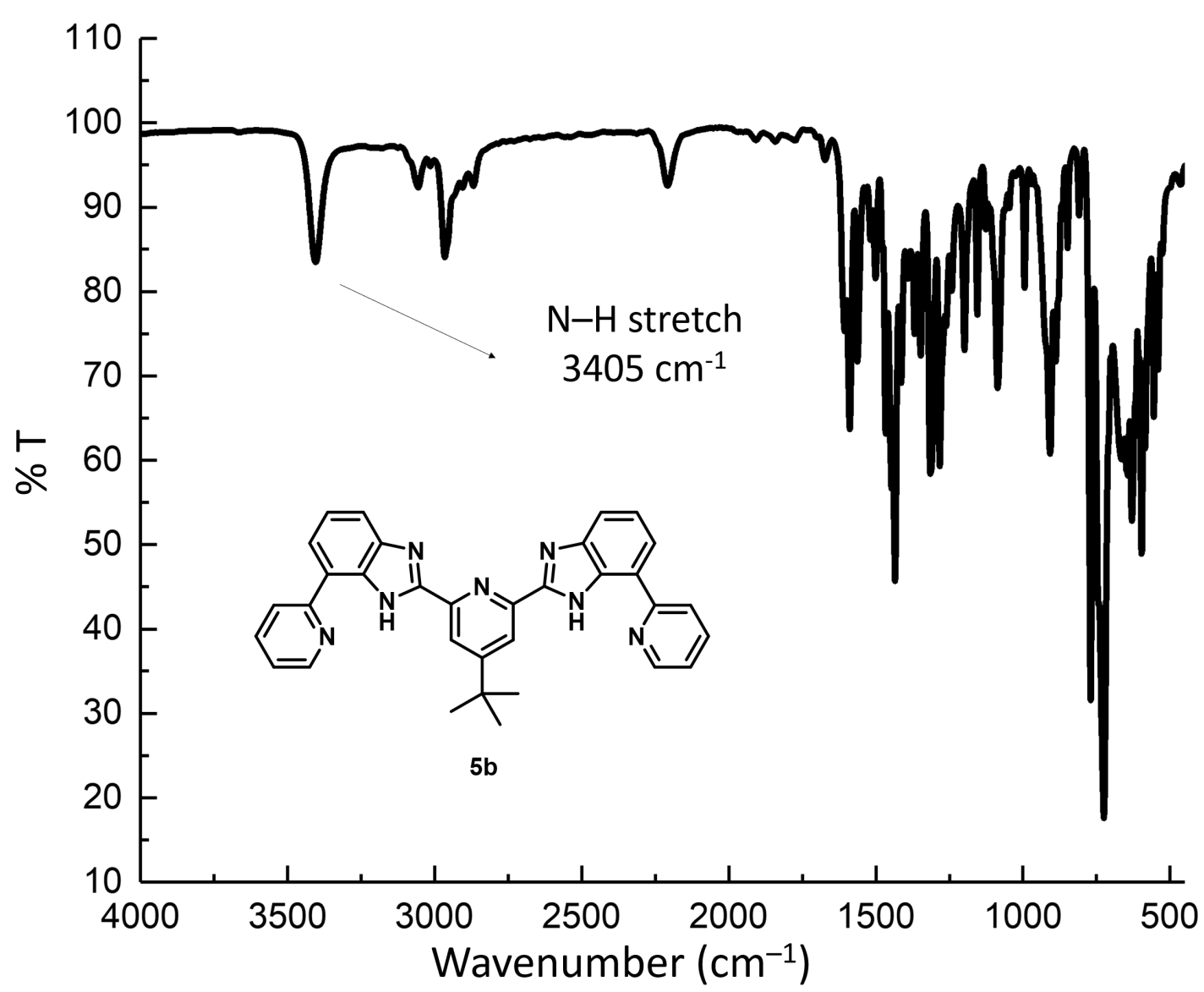

Fig. S7. FT-IR (ATR) spectrum of **5b**.

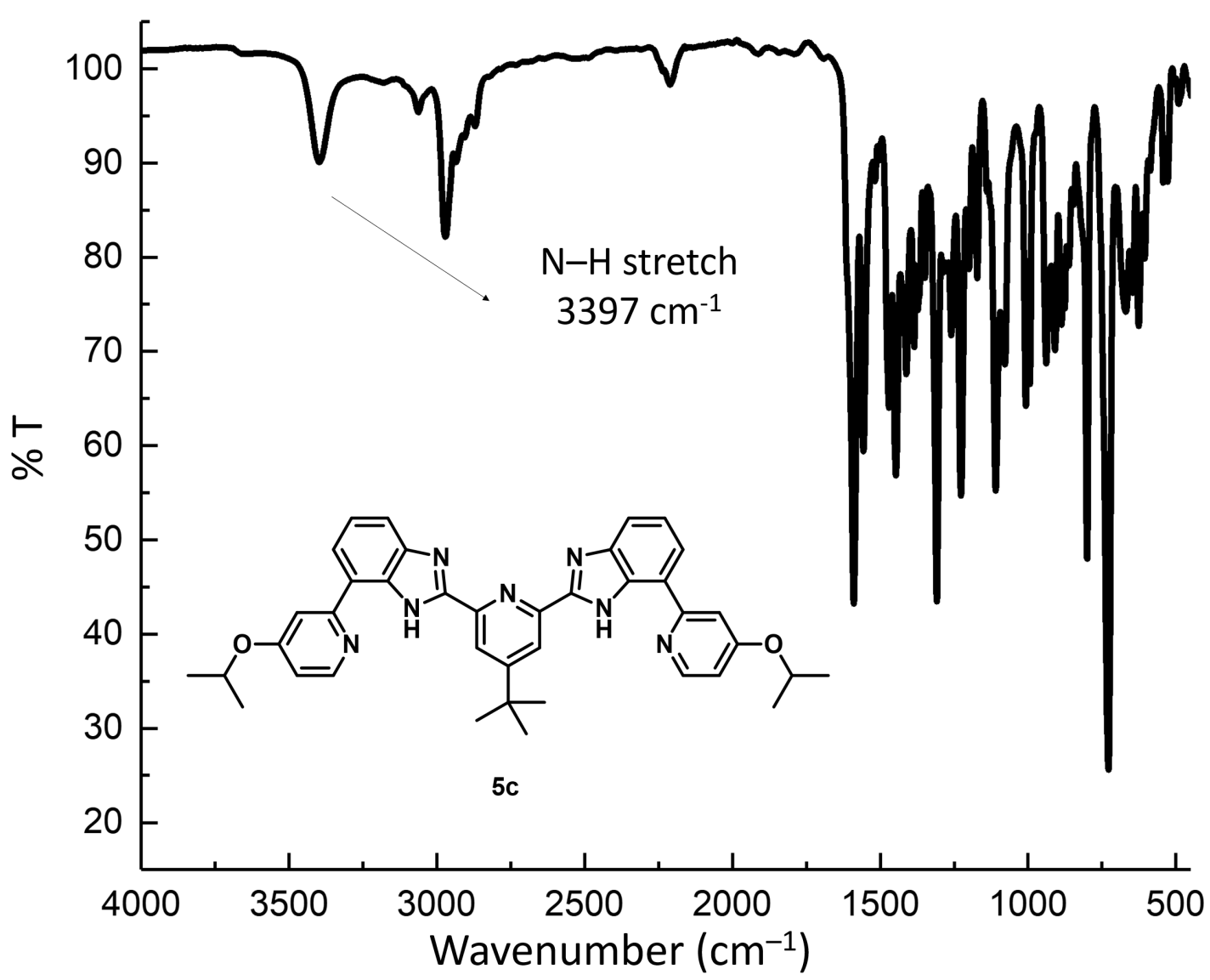

Fig. S8. FT-IR (ATR) spectrum of **5c**.

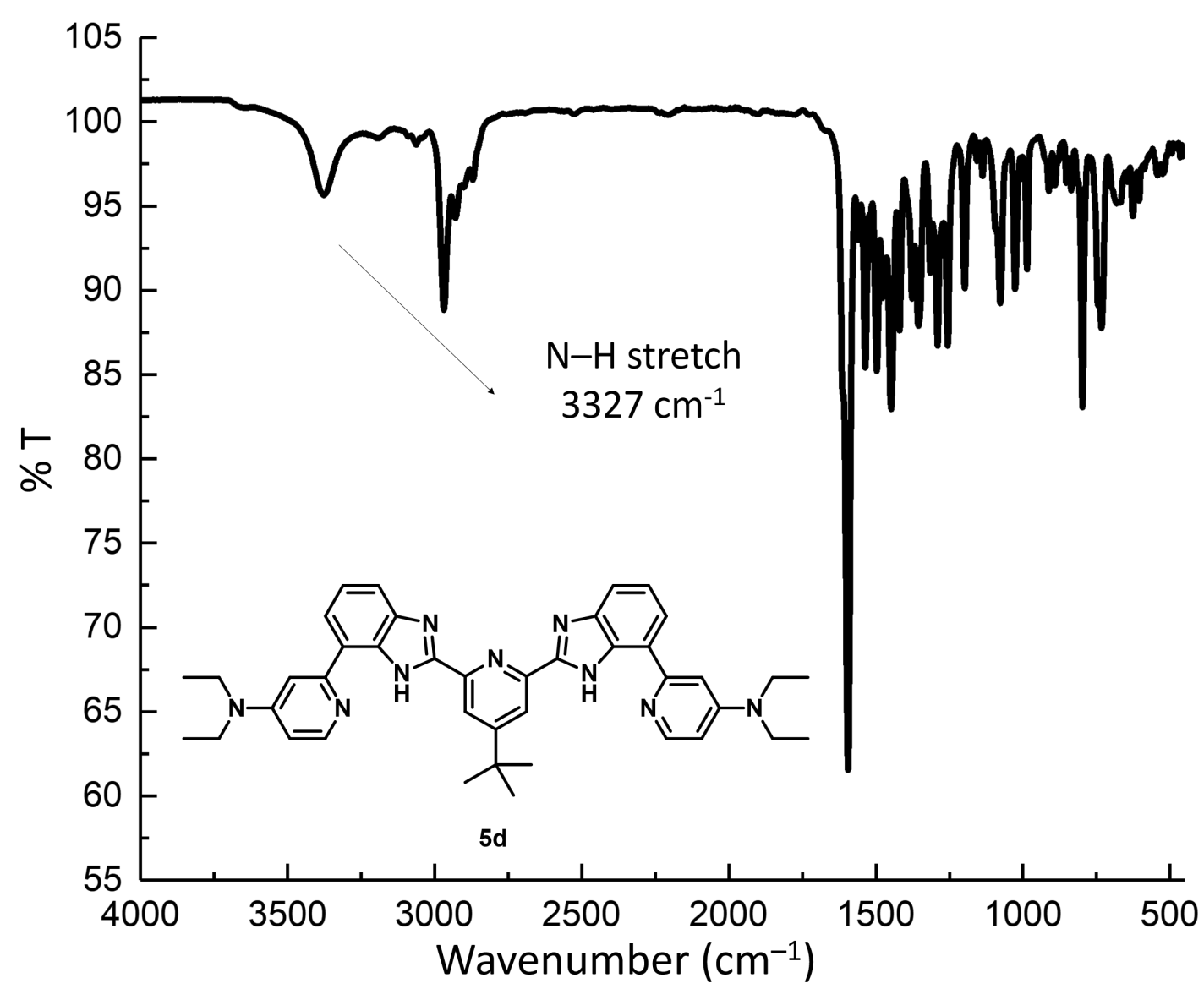

Fig. S9. FT-IR (ATR) spectrum of **5d**.

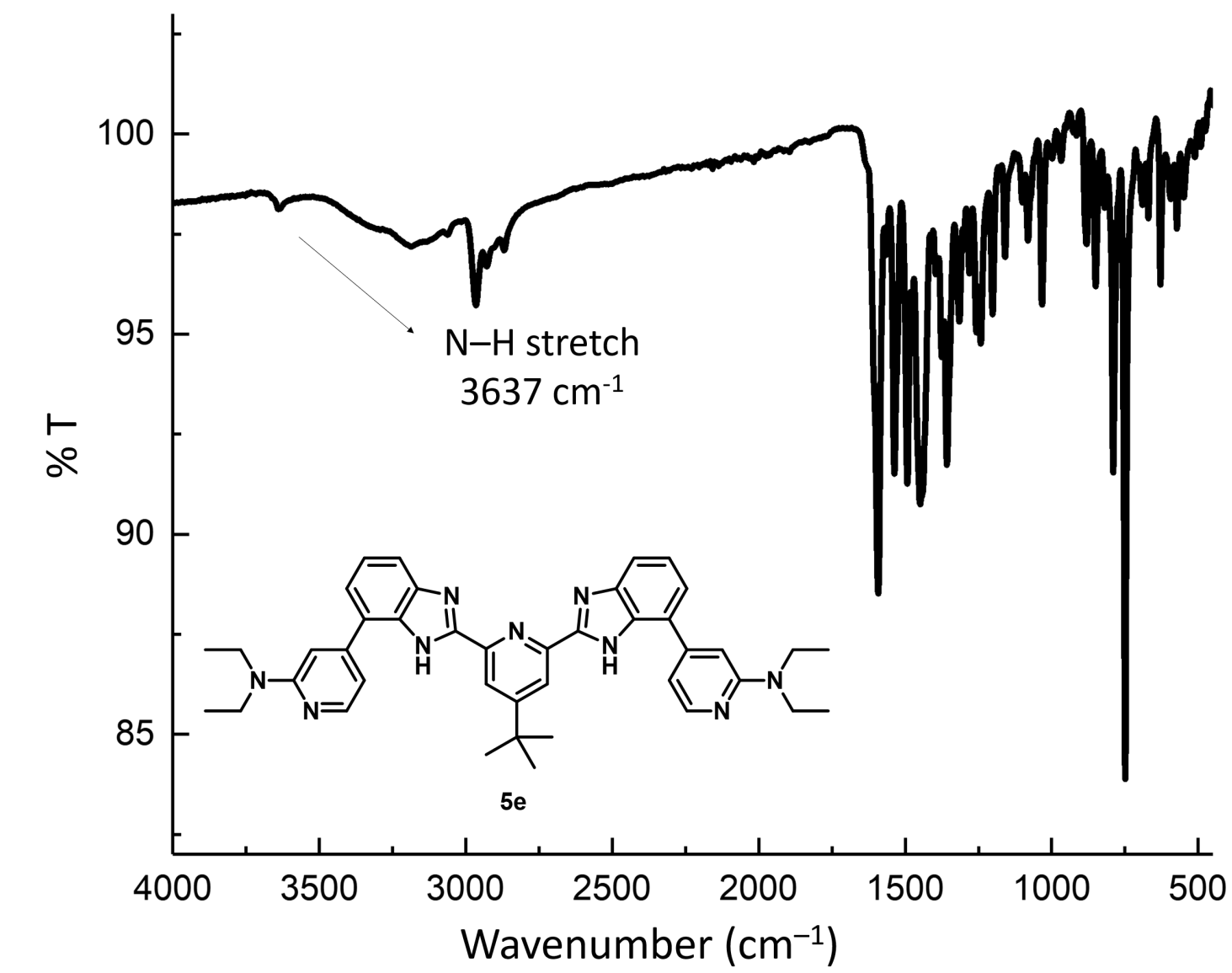

Fig. S10. FT-IR (ATR) spectrum of **5e**.

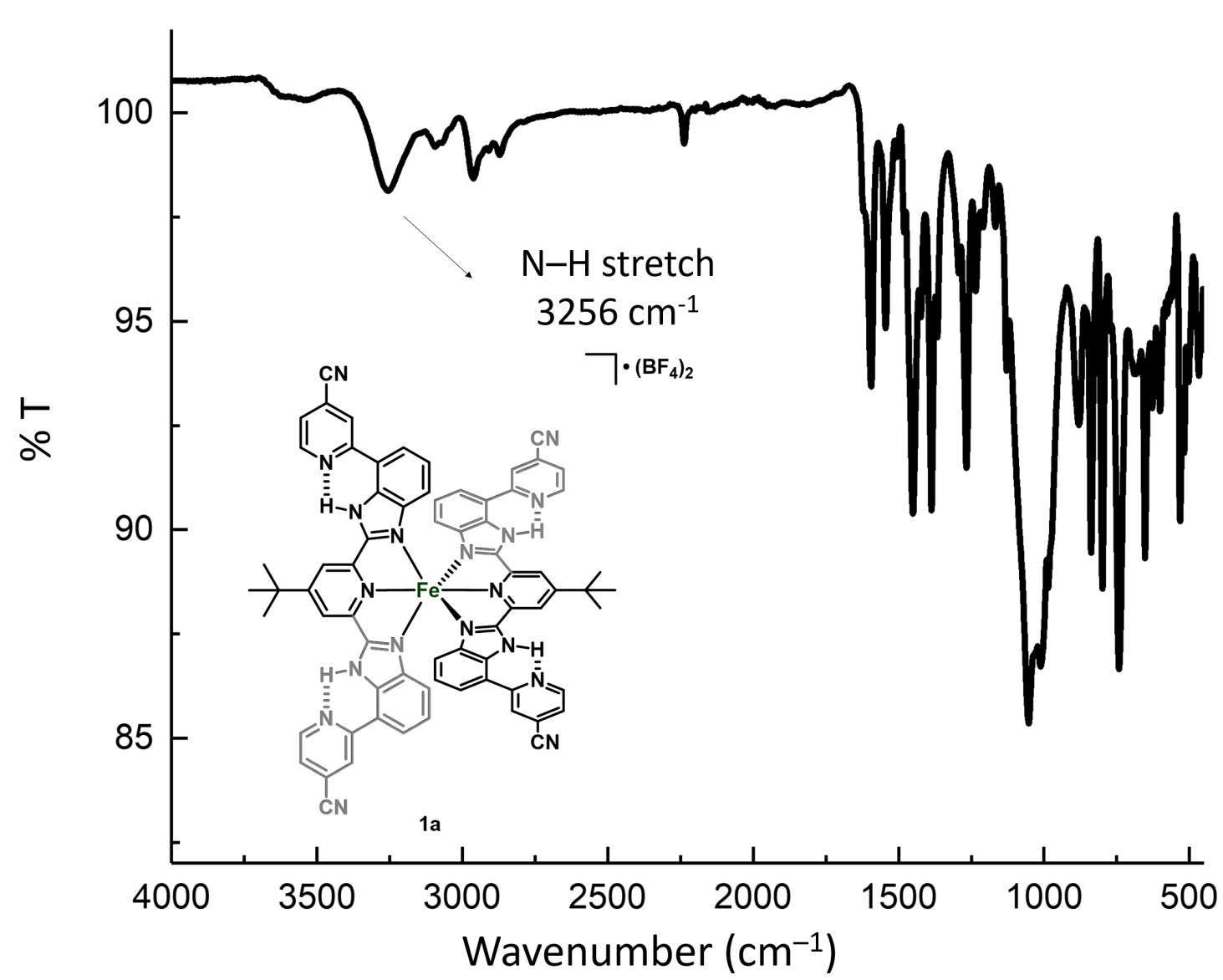

Fig. S11. FT-IR (ATR) spectrum of **1a**.

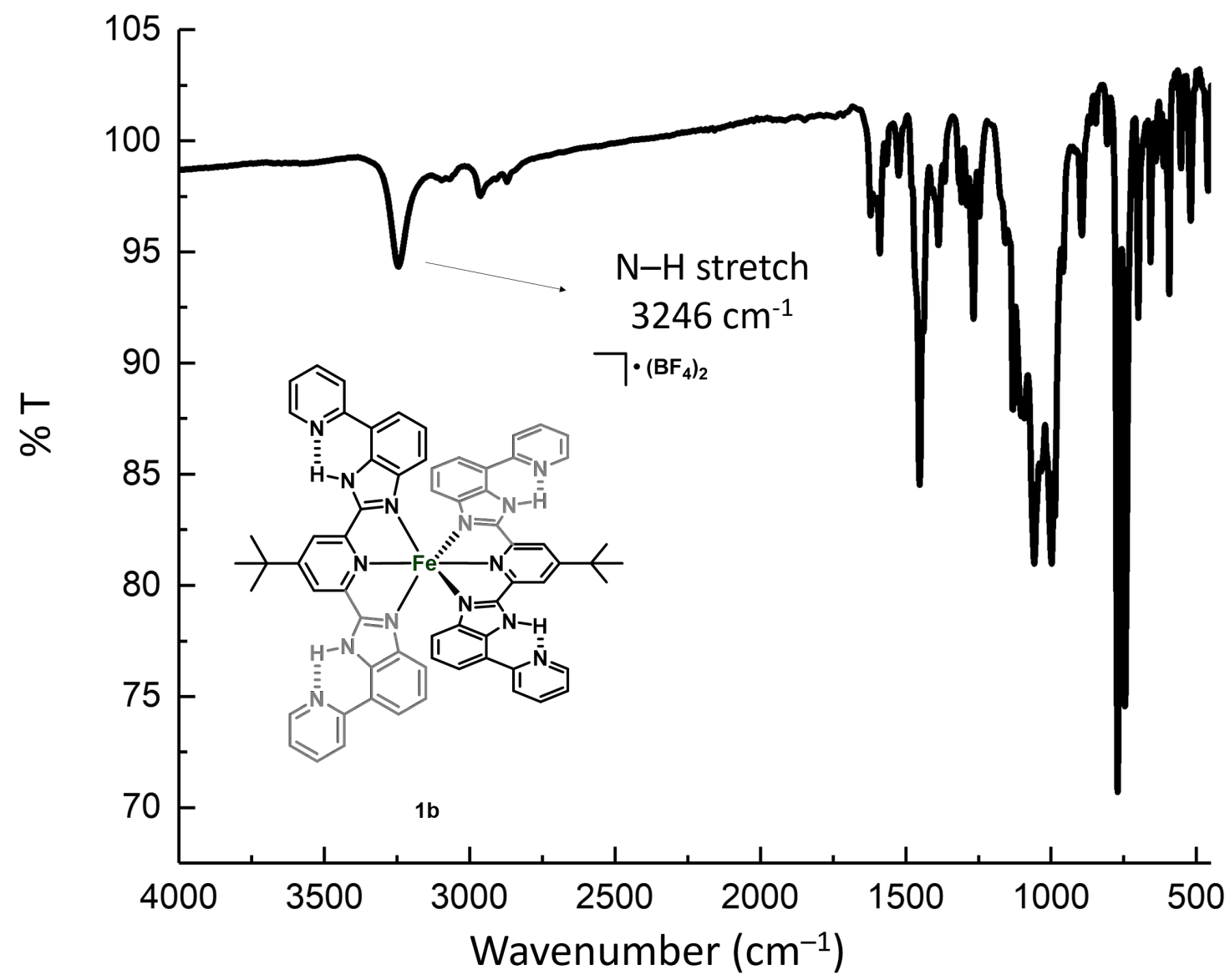

Fig. S12. FT-IR (ATR) spectrum of **1b**.

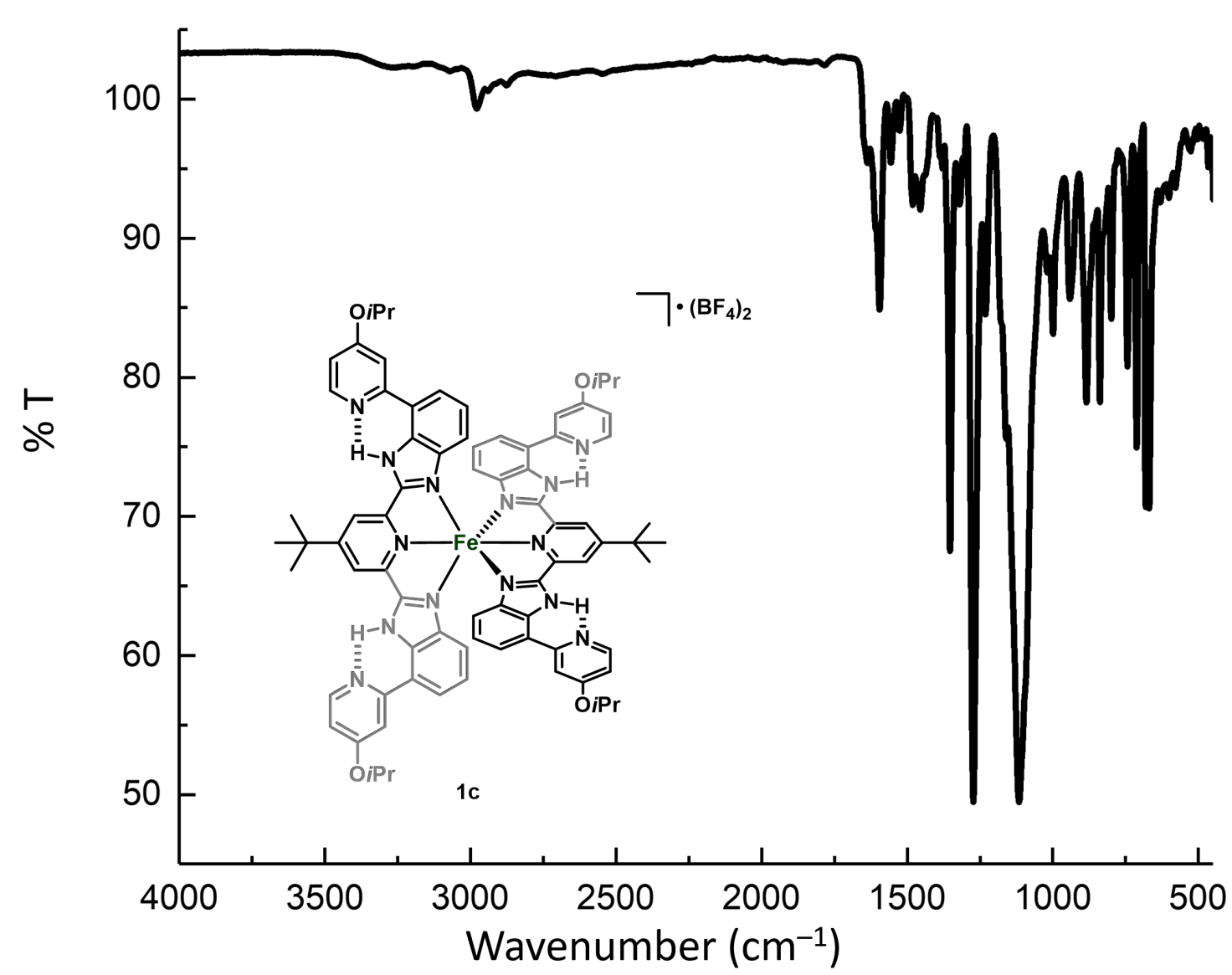

Fig. S13. FT-IR (ATR) spectrum of **1c**. The N–H stretching modes could not be resolved owing to the extensive band broadening arising from anharmonic coupling of strong intramolecular N–H···N hydrogen bonds.

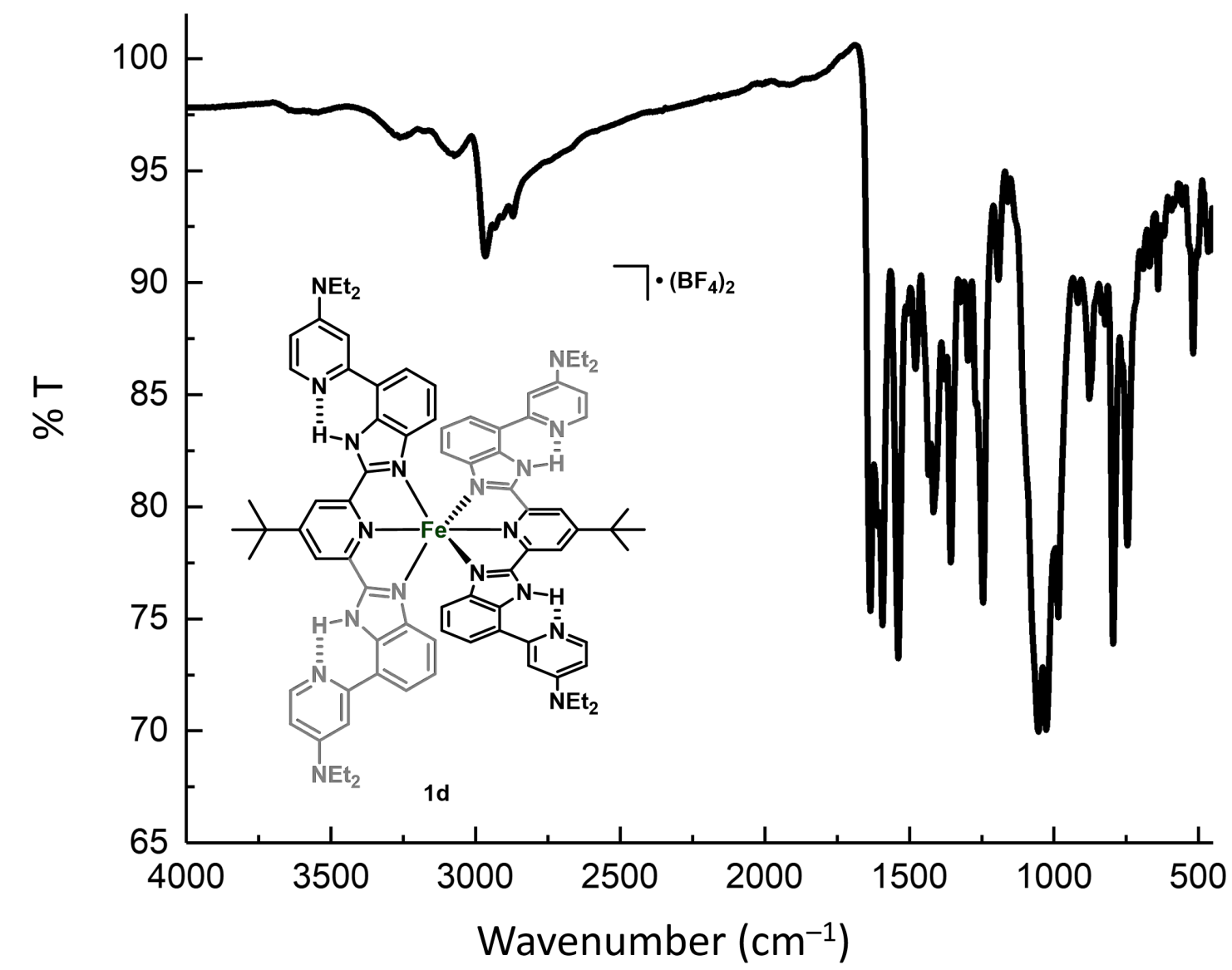

Fig. S14. FT-IR (ATR) spectrum of **1d**. The N–H stretching modes could not be resolved owing to the extensive band broadening arising from anharmonic coupling of strong intramolecular N–H···N hydrogen bonds.

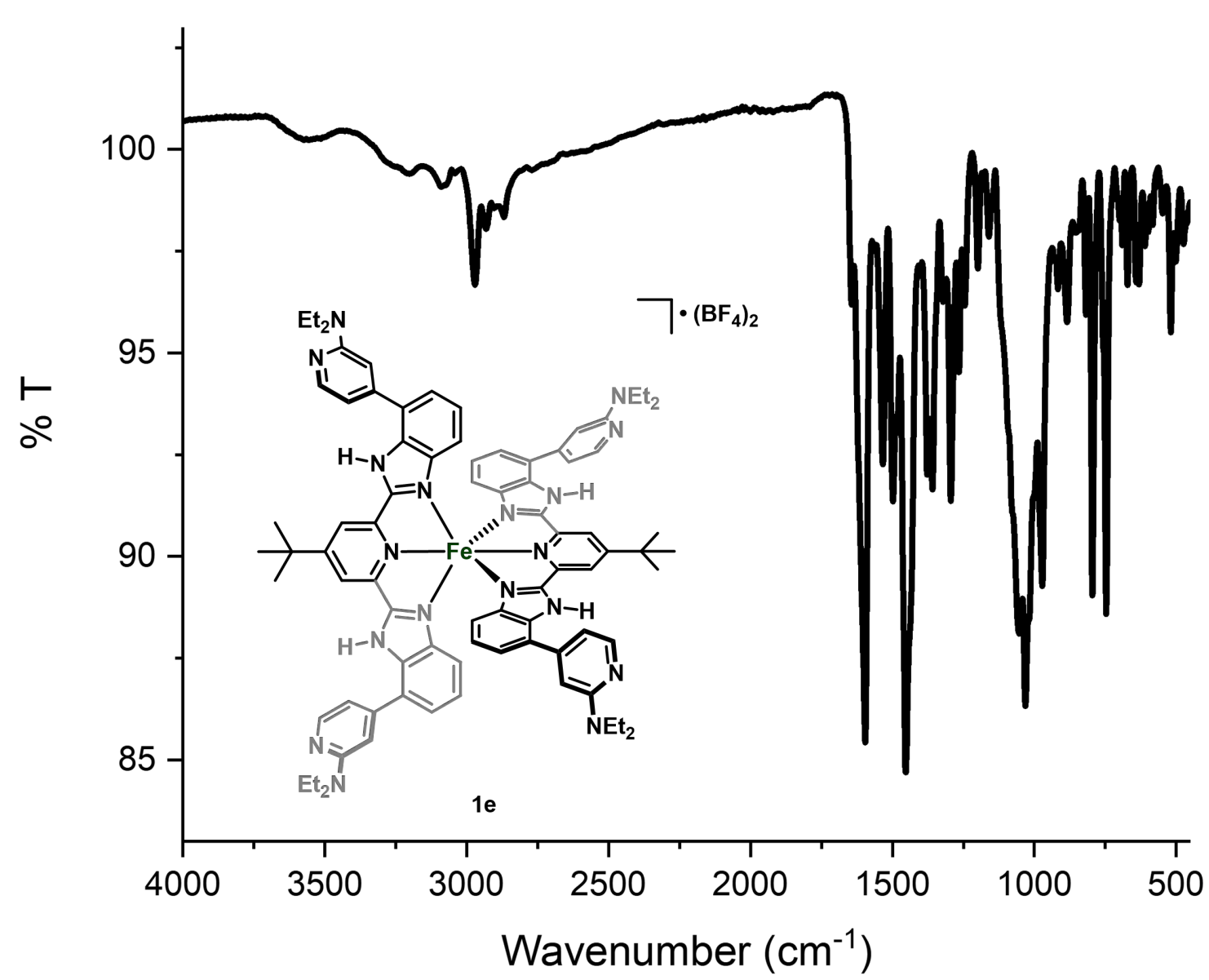

Fig. S15. FT-IR (ATR) spectrum of **1e**.

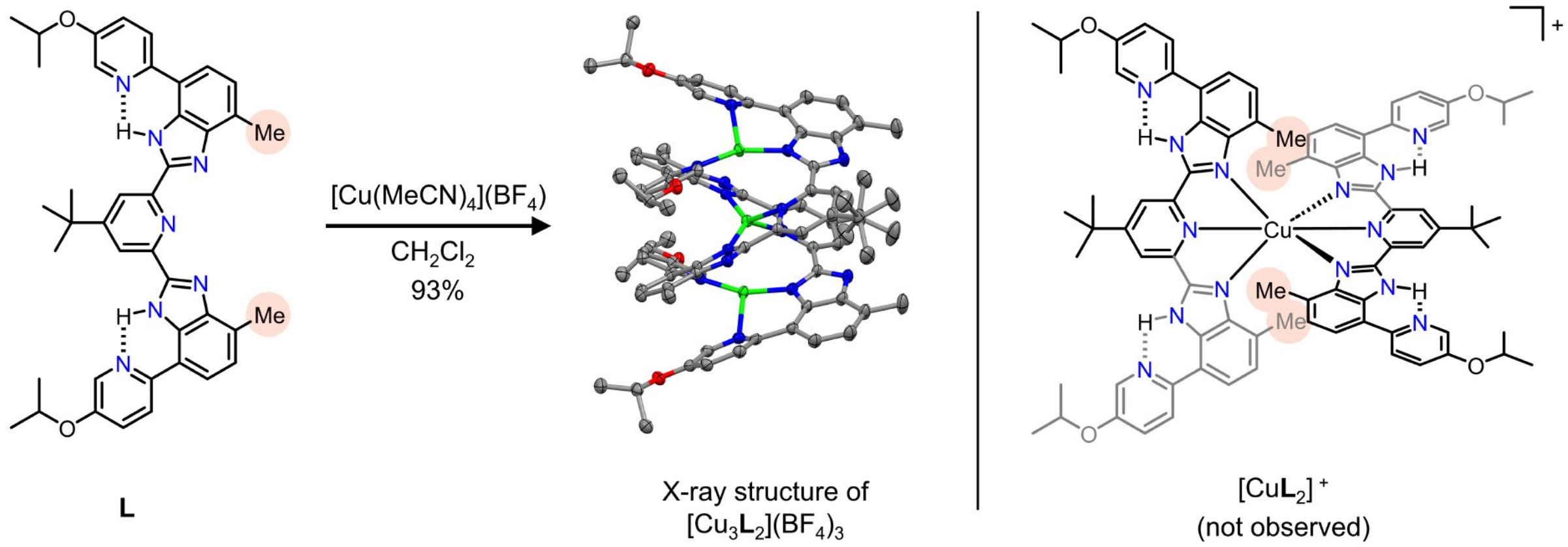

Fig. S16. Synthesis of the tricopper(I) helicate $[Cu_3(\mathbf{L})_2][BF_4]_3$.[10] Reaction of **L**, a ligand analogous to **5a–d** but bearing two additional methyl groups, with a Cu(I) source affords a trinuclear double helicate rather than a mononuclear bis(chelate) complex. The steric constraints imposed by the methyl groups prevent the bis(tridentate) coordination mode.

[10] H. Kim, J. Shin, S. Kim and D. Lee, *Chem. Sci.*, 2023, **14**, 3265–3269.

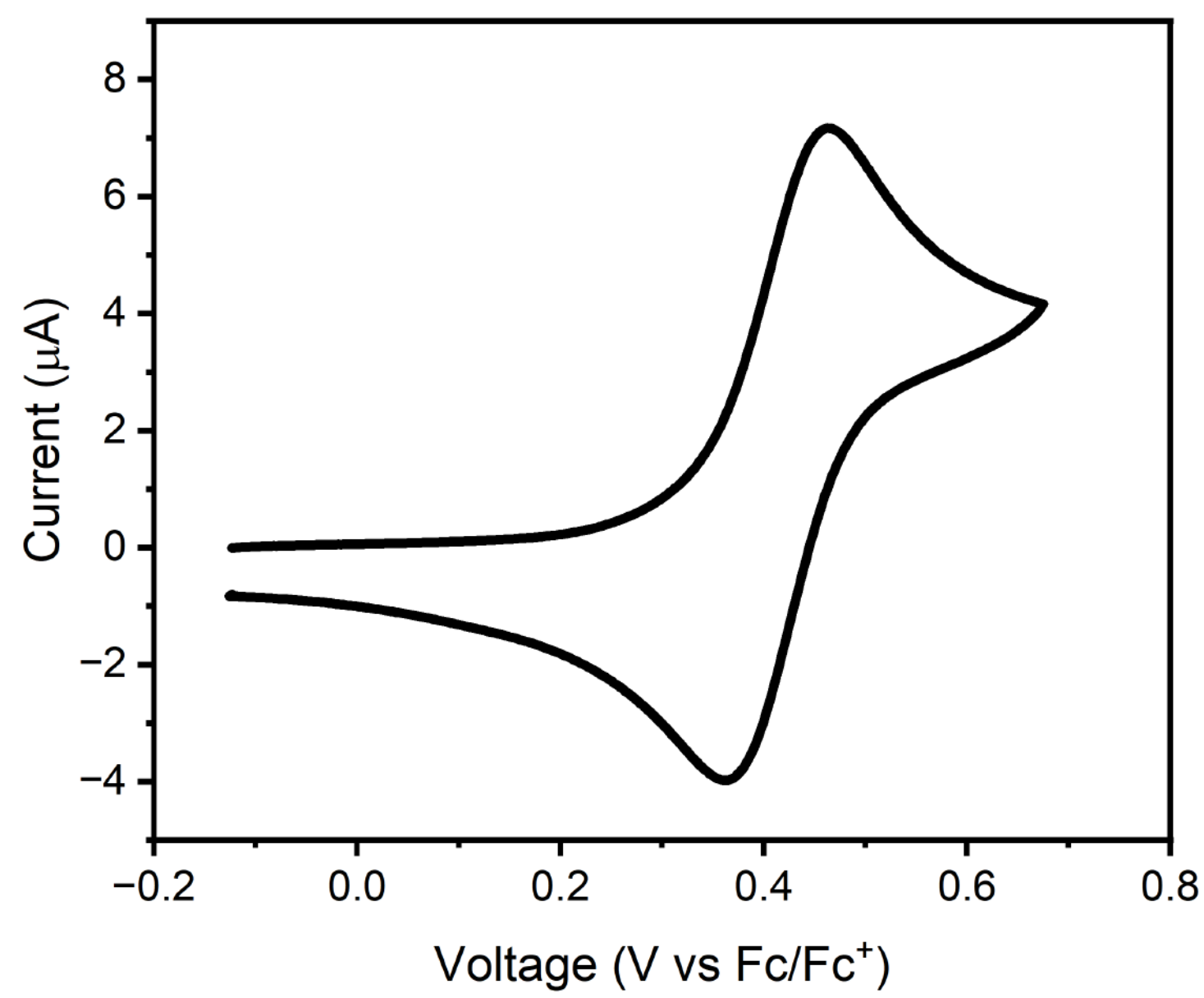

Fig. S17. Cyclic voltammogram of **1a** in MeCN (sample concentration = 1 mM) on the Pt working electrode with n-Bu_4NPF_6 (0.1 M) as the supporting electrolyte. Scan rate = 100 mV/s.

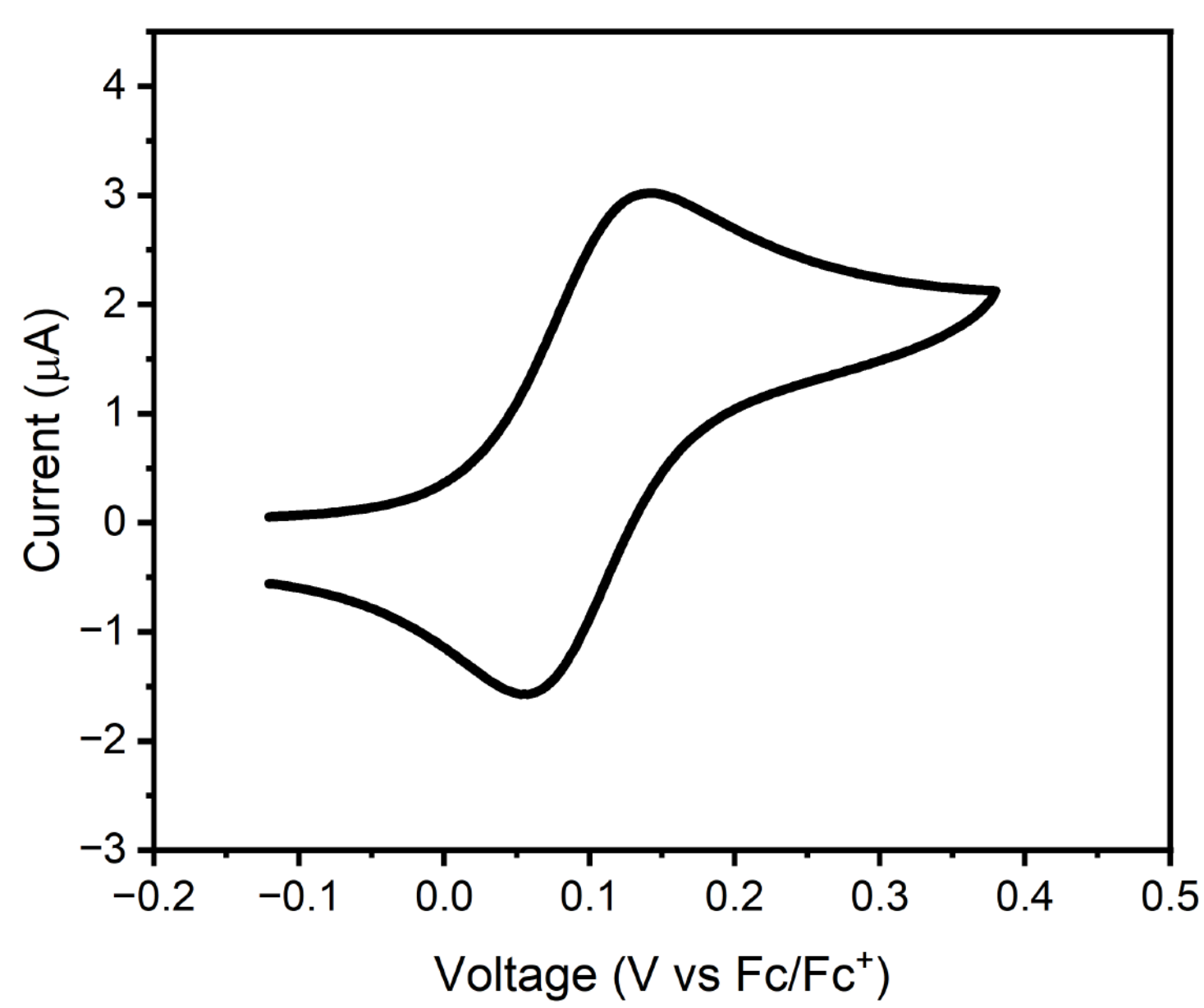

Fig. S18. Cyclic voltammogram of **1b** in MeCN (sample concentration = 1 mM) on the Pt working electrode with n-Bu_4NPF_6 (0.1 M) as the supporting electrolyte. Scan rate = 100 mV/s.

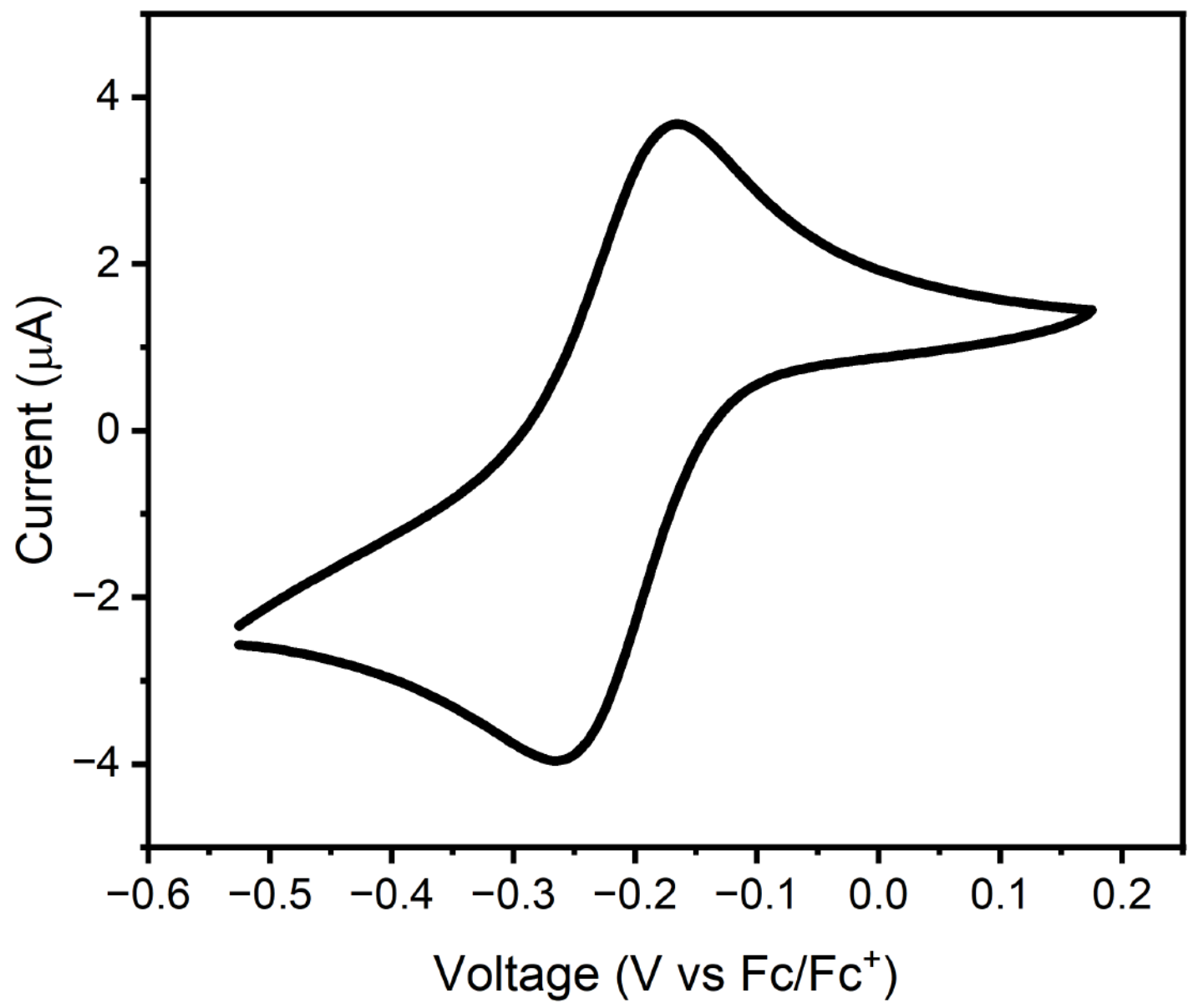

Fig. S19. Cyclic voltammogram of **1c** in MeCN (sample concentration = 1 mM) on the Pt working electrode with n-Bu_4NPF_6 (0.1 M) as the supporting electrolyte. Scan rate = 100 mV/s.

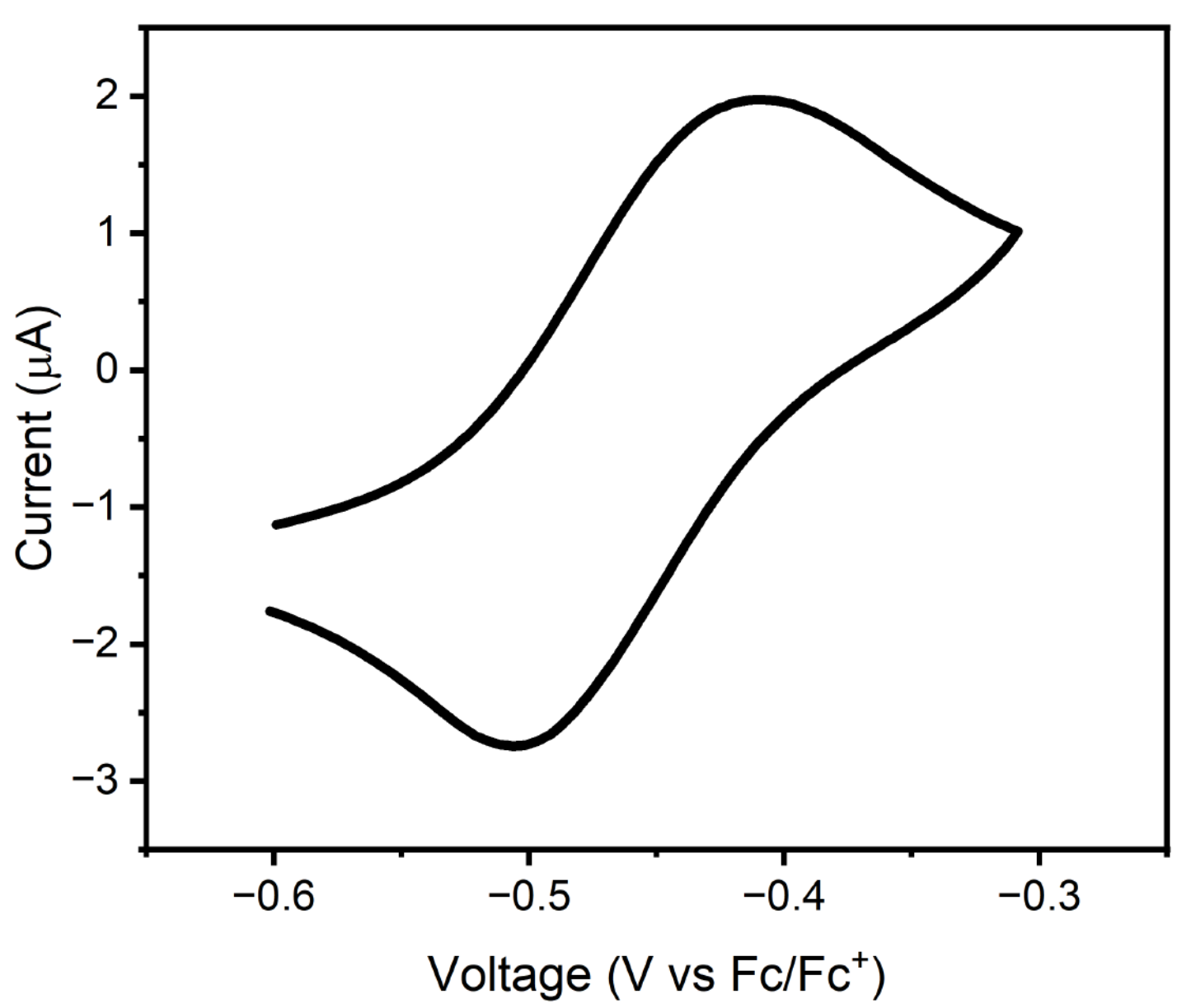

Fig. S20. Cyclic voltammogram of **1d** in MeCN (sample concentration = 1 mM) on the Pt working electrode with n-Bu_4NPF_6 (0.1 M) as the supporting electrolyte. Scan rate = 100 mV/s.

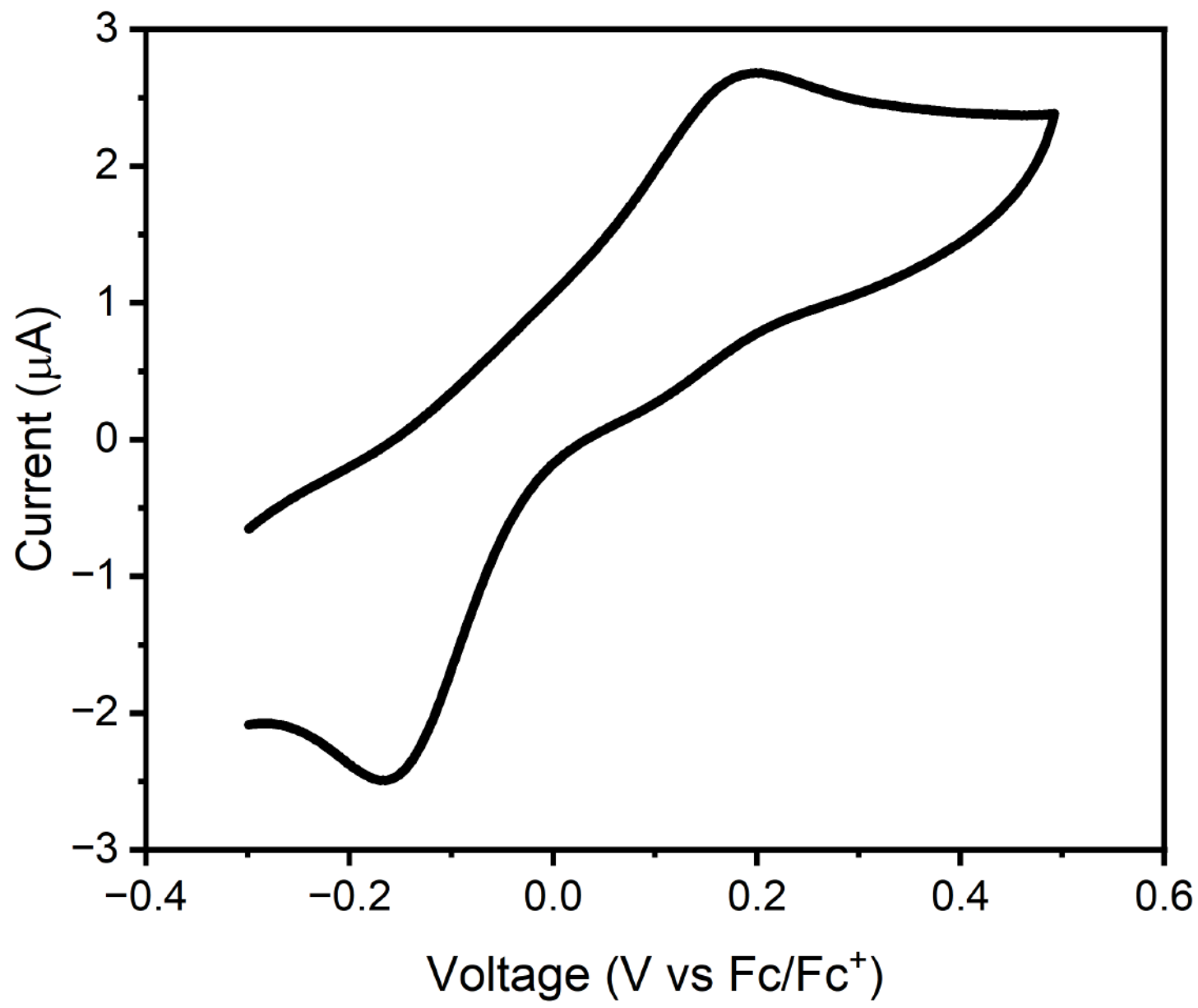

Fig. S21. Cyclic voltammogram of **1e** in MeCN (sample concentration = 1 mM) on the Pt working electrode with n-Bu_4NPF_6 (0.1 M) as the supporting electrolyte. Scan rate = 100 mV/s.

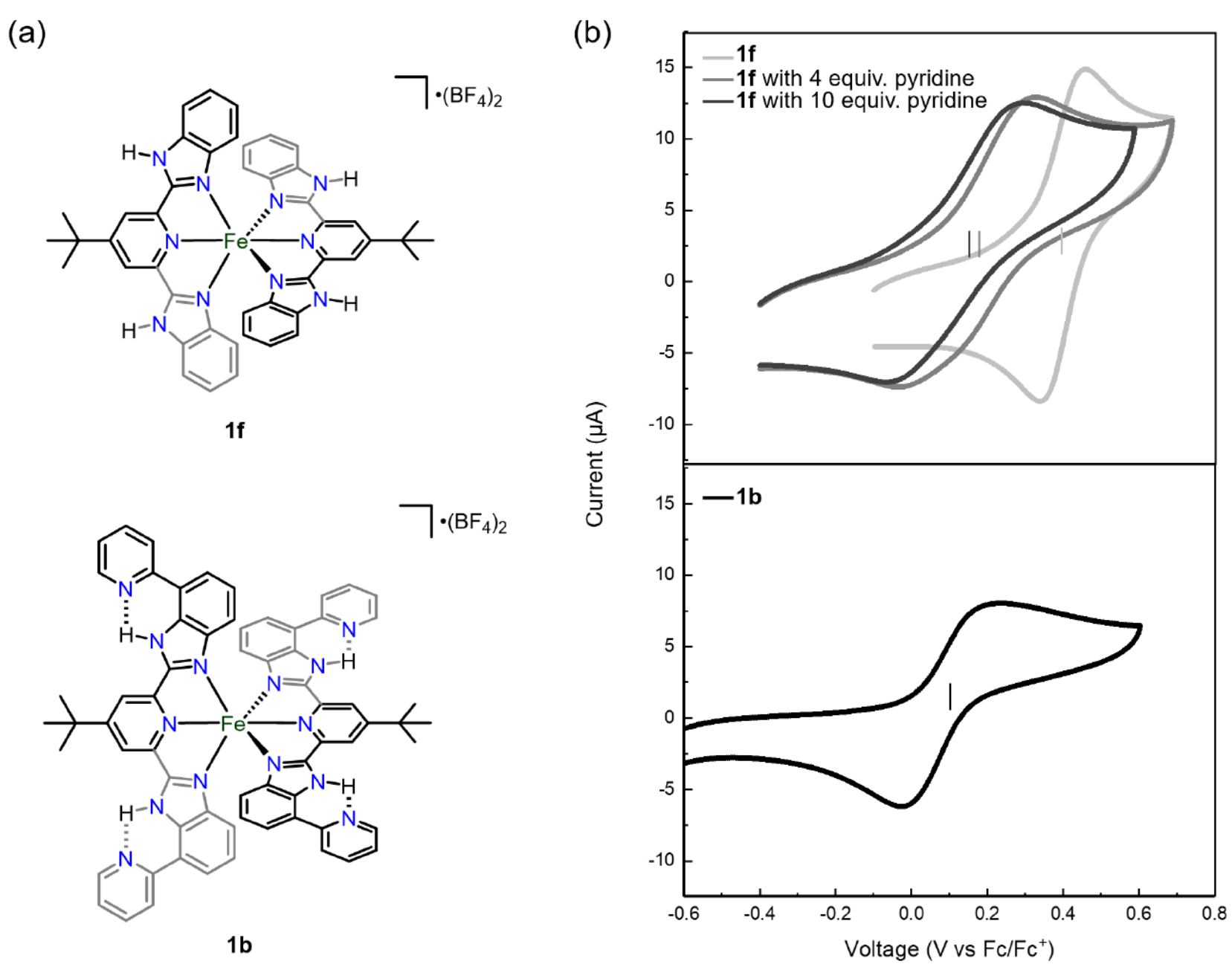

Fig. S22. (a) Chemical structures of **1b** and **1f**. (b) Cyclic voltammograms of **1f** in MeCN in the absence and presence of exogenous pyridine (0, 4, and 10 equiv.; top) and of **1b** in MeCN (bottom). Vertical bars denote the half-wave potential ($E_{1/2}$) of each voltammogram. Sample concentration = 1 mM; glassy carbon working electrode; n-Bu_4NPF_6 (0.1 M) supporting electrolyte; scan rate = 100 mV/s.

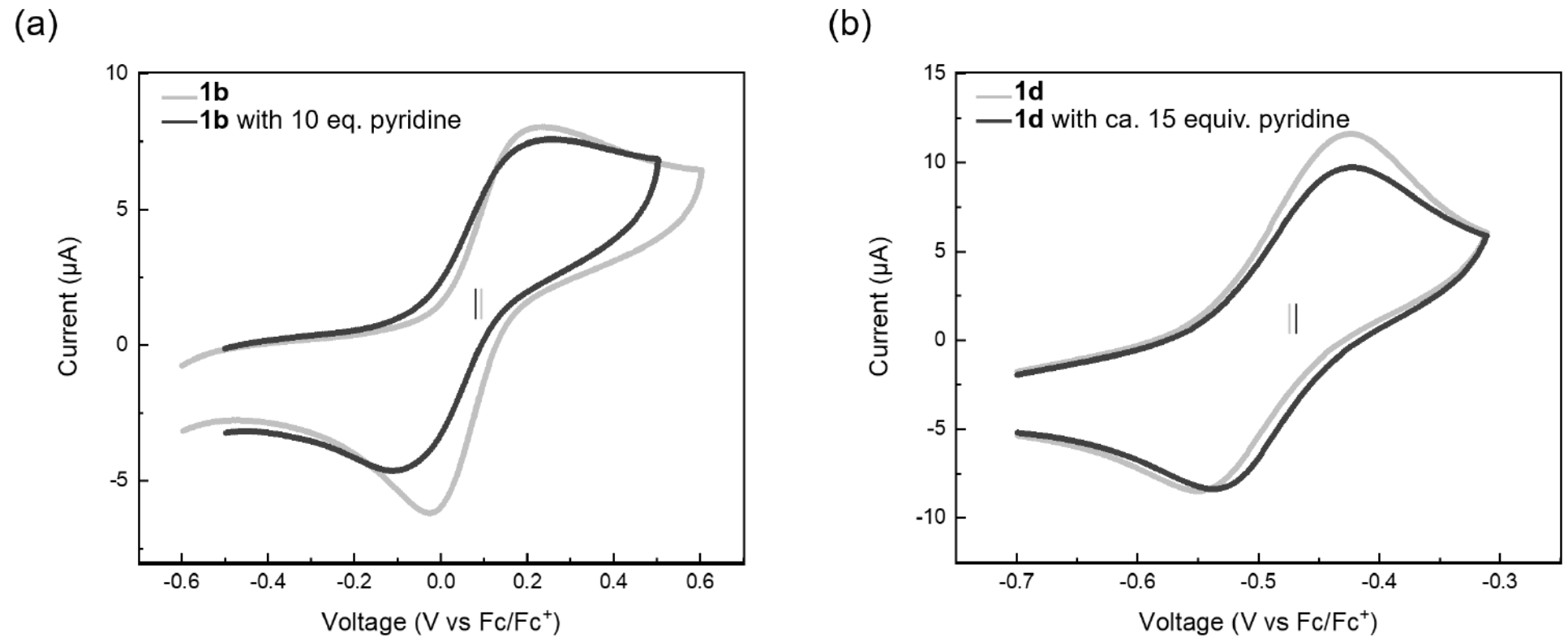

Fig. S23. Cyclic voltammograms of (a) **1b** and (b) **1d** in MeCN in the absence and presence of exogenous pyridine. Vertical bars denote the half-wave potential ($E_{1/2}$) of each voltammogram. Sample concentration = 1 mM; glassy carbon working electrode; n-Bu_4NPF_6 (0.1 M) supporting electrolyte; scan rate = 100 mV/s.

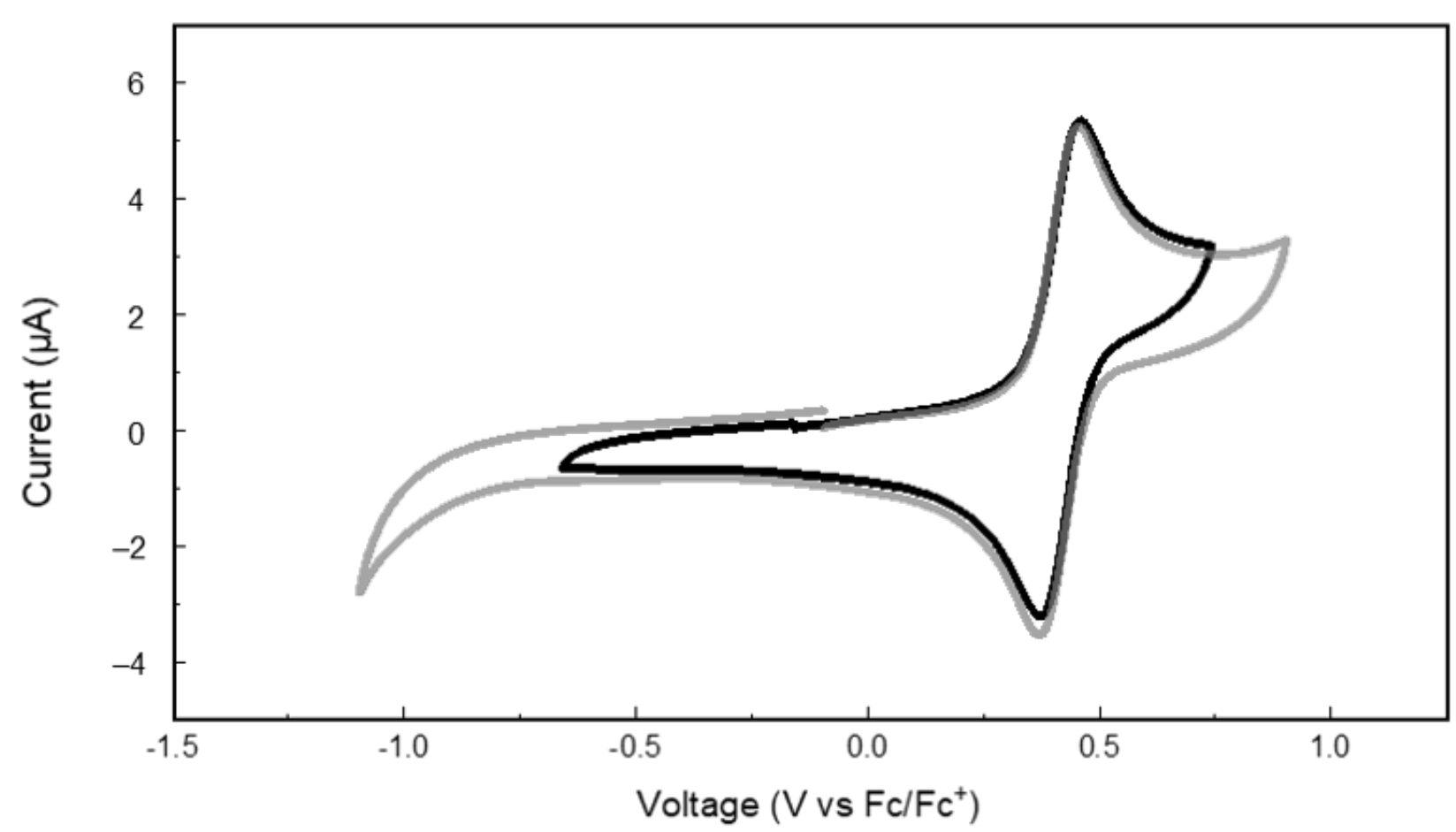

Fig. S24. Cyclic voltammogram of **1a** in MeCN (sample concentration = 1 mM) on the Pt working electrode with n-Bu_4NPF_6 (0.1 M) as the supporting electrolyte. The full-range voltammogram (grey) and the voltammogram over a narrower potential window (black) are overlaid. Scan rate = 100 mV/s.

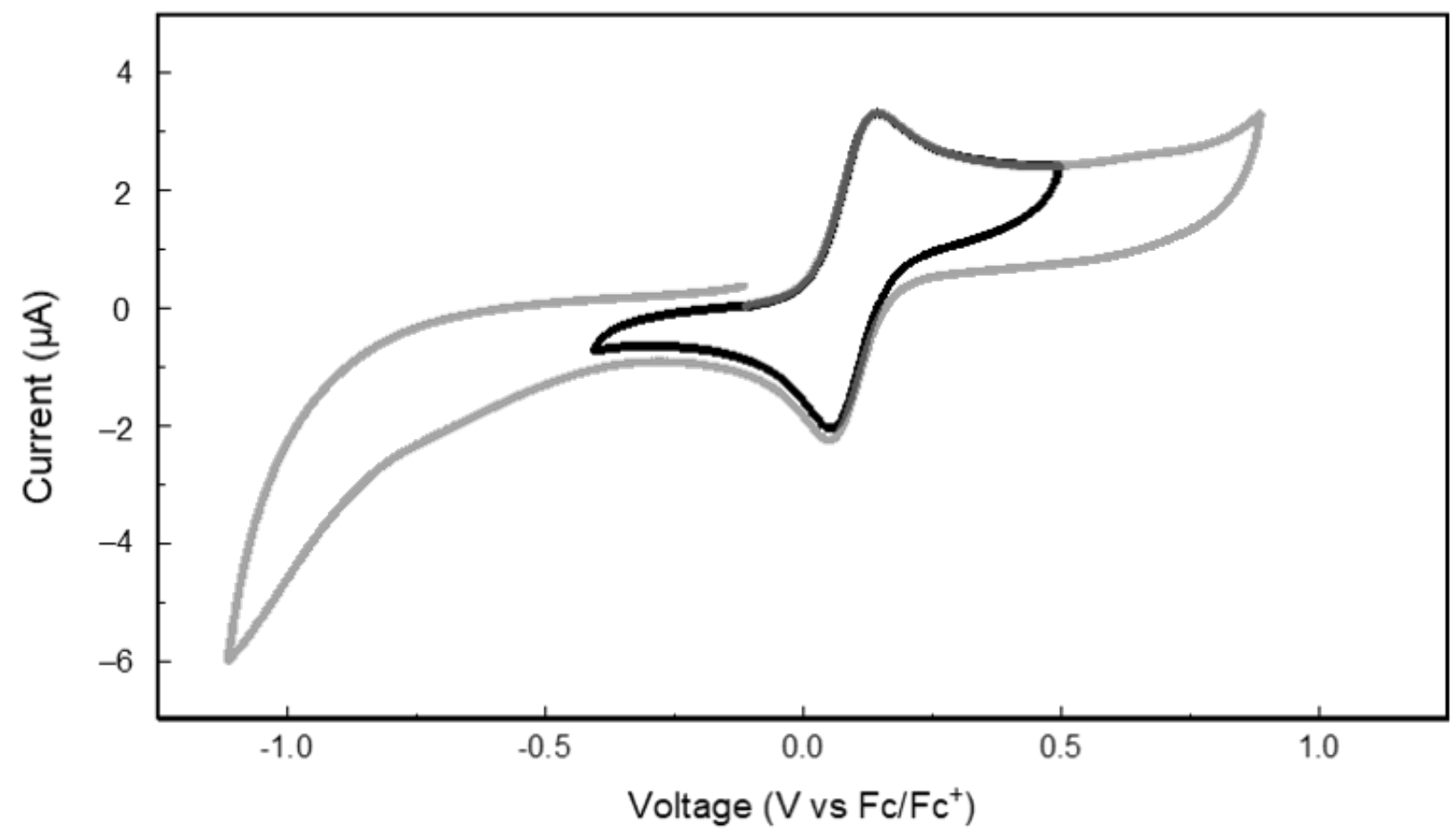

Fig. S25. Cyclic voltammogram of **1b** in MeCN (sample concentration = 1 mM) on the Pt working electrode with *n*-Bu_4NPF_6 (0.1 M) as the supporting electrolyte. The full-range voltammogram (grey) and the voltammogram over a narrower potential window (black) are overlaid. Scan rate = 100 mV/s.

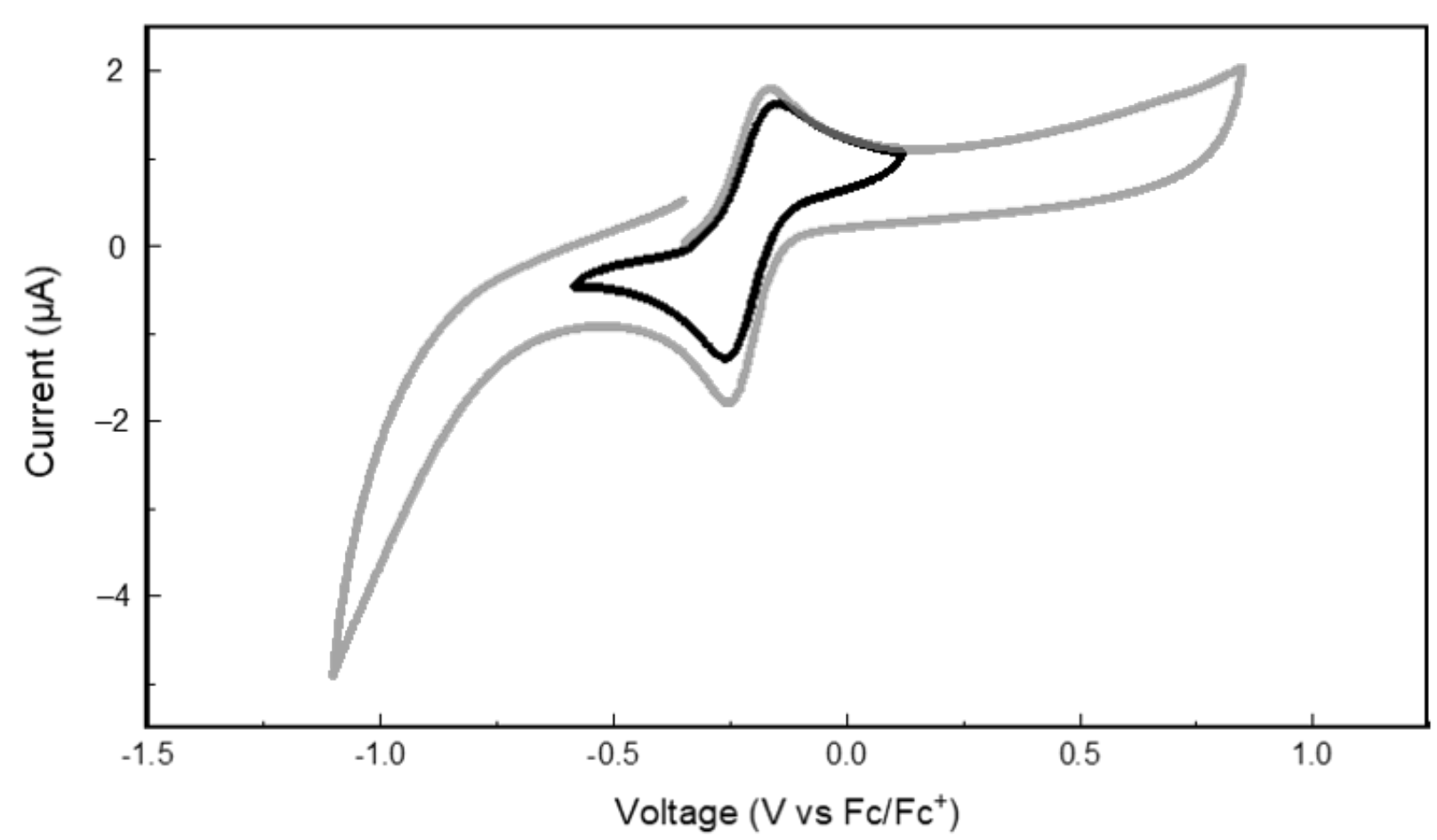

Fig. S26. Cyclic voltammogram of **1c** in MeCN (sample concentration = 1 mM) on the Pt working electrode with *n*-Bu_4NPF_6 (0.1 M) as the supporting electrolyte. The full-range voltammogram (grey) and the voltammogram over a narrower potential window (black) are overlaid. Scan rate = 100 mV/s.

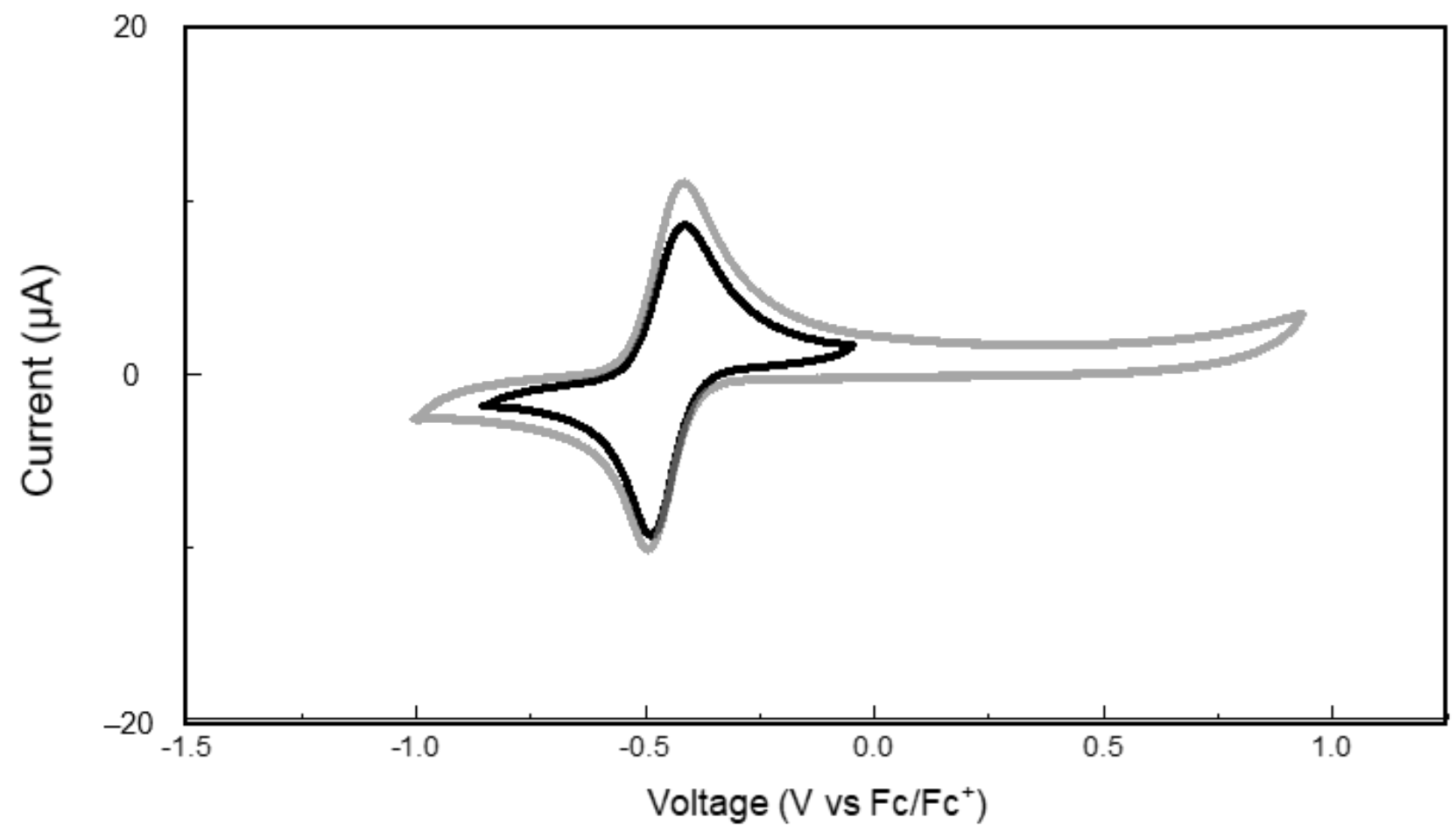

Fig. S27. Cyclic voltammogram of **1d** in MeCN (sample concentration = 1 mM) on the Pt working electrode with n-Bu_4NPF_6 (0.1 M) as the supporting electrolyte. The full-range voltammogram (grey) and the voltammogram over a narrower potential window (black) are overlaid. Scan rate = 100 mV/s. T = 40 °C.

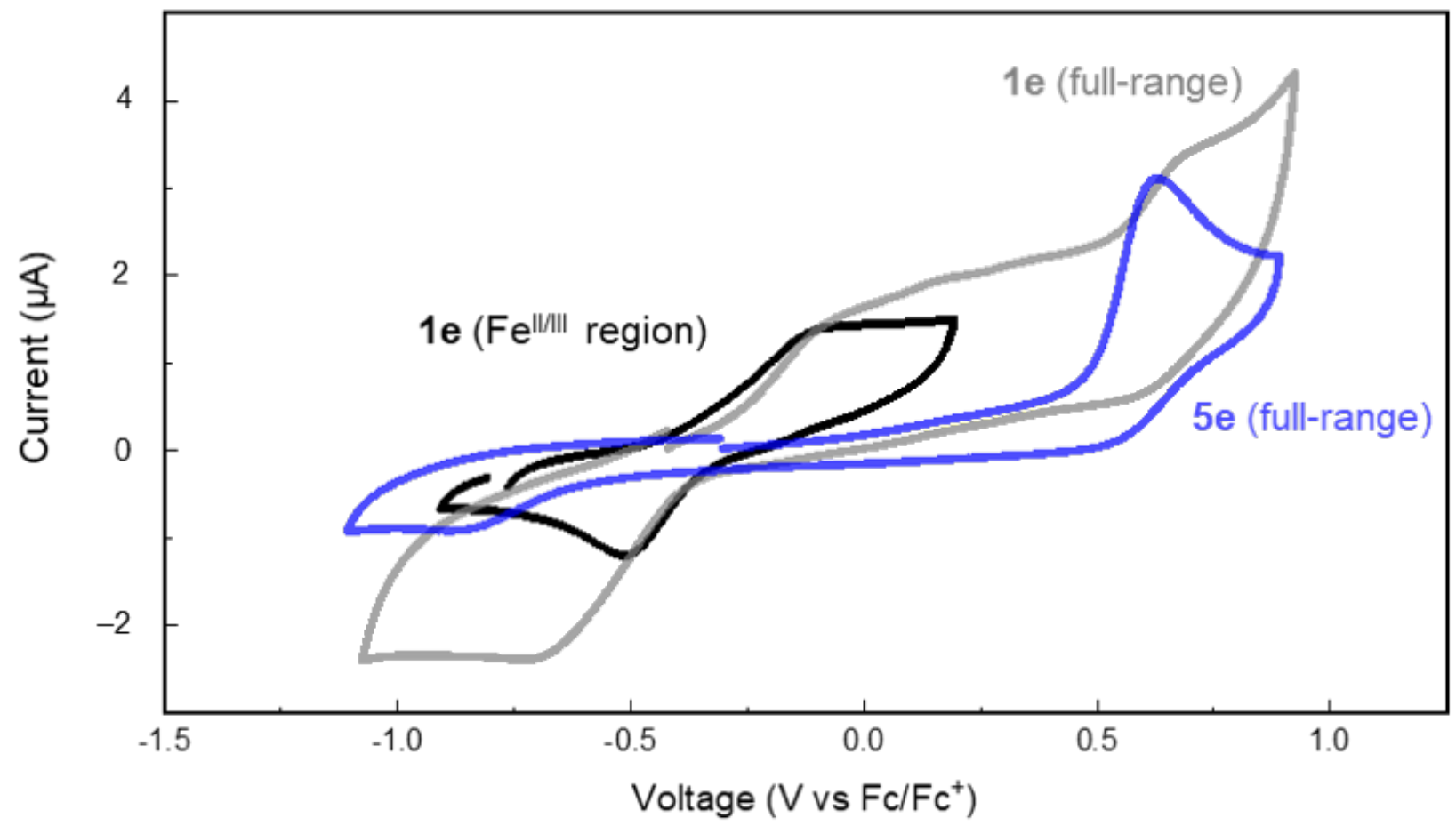

Fig. S28. Cyclic voltammograms of **1e** (grey for the full range; black for a narrower potential window) and its corresponding free ligand **5e** (blue), recorded separately in MeCN (sample concentration = 1 mM) on a Pt working electrode with n-Bu_4NPF_6 (0.1 M) as the supporting electrolyte. Scan rate = 100 mV/s. The irreversible Fe-centered oxidation of **1e** appears at $E_{p,a}$ = +0.20 V. The additional anodic wave at $E_{p,a}$ = +0.65 V is close to the irreversible oxidation of free ligand **5e** ($E_{p,a} \approx$ +0.60 V), supporting its assignment to oxidation of the metal-bound, –NEt_2-containing ligand. Unlike **1d**, **1e** lacks intramolecular N–H···N hydrogen bonds, so its –NEt_2 groups are not electronically coupled to the Fe center through this pathway. The ligand-centered oxidation of **1e** remains near the free-ligand potential and is well separated from its Fe-centered oxidation, consistent with the absence of electronic coupling through this hydrogen-bonding pathway.

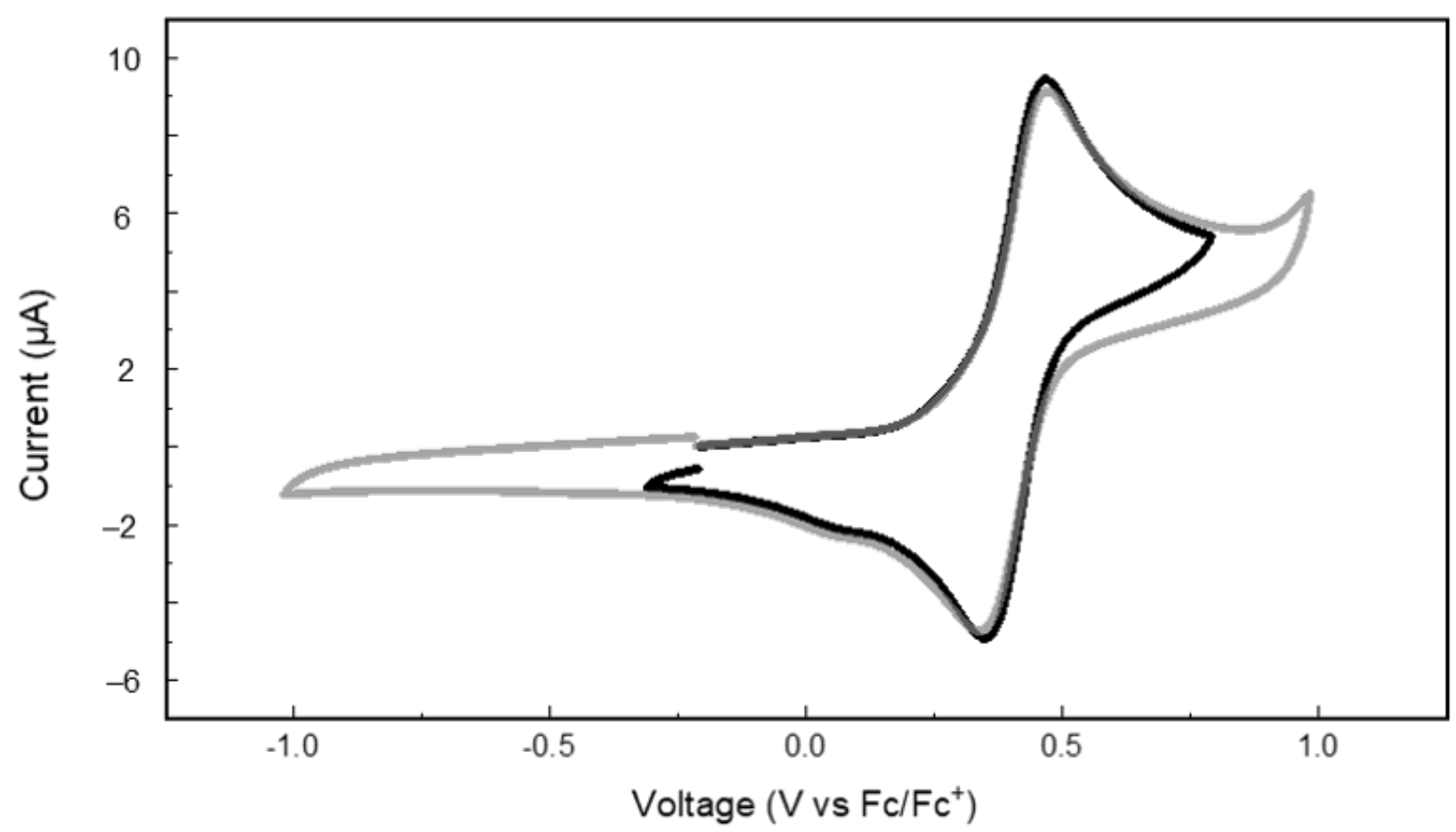

Fig. S29. Cyclic voltammogram of **1f** in MeCN (sample concentration = 1 mM) on the Pt working electrode with n-Bu_4NPF_6 (0.1 M) as the supporting electrolyte. The full-range voltammogram (grey) and the voltammogram over a narrower potential window (black) are overlaid. Scan rate = 100 mV/s.

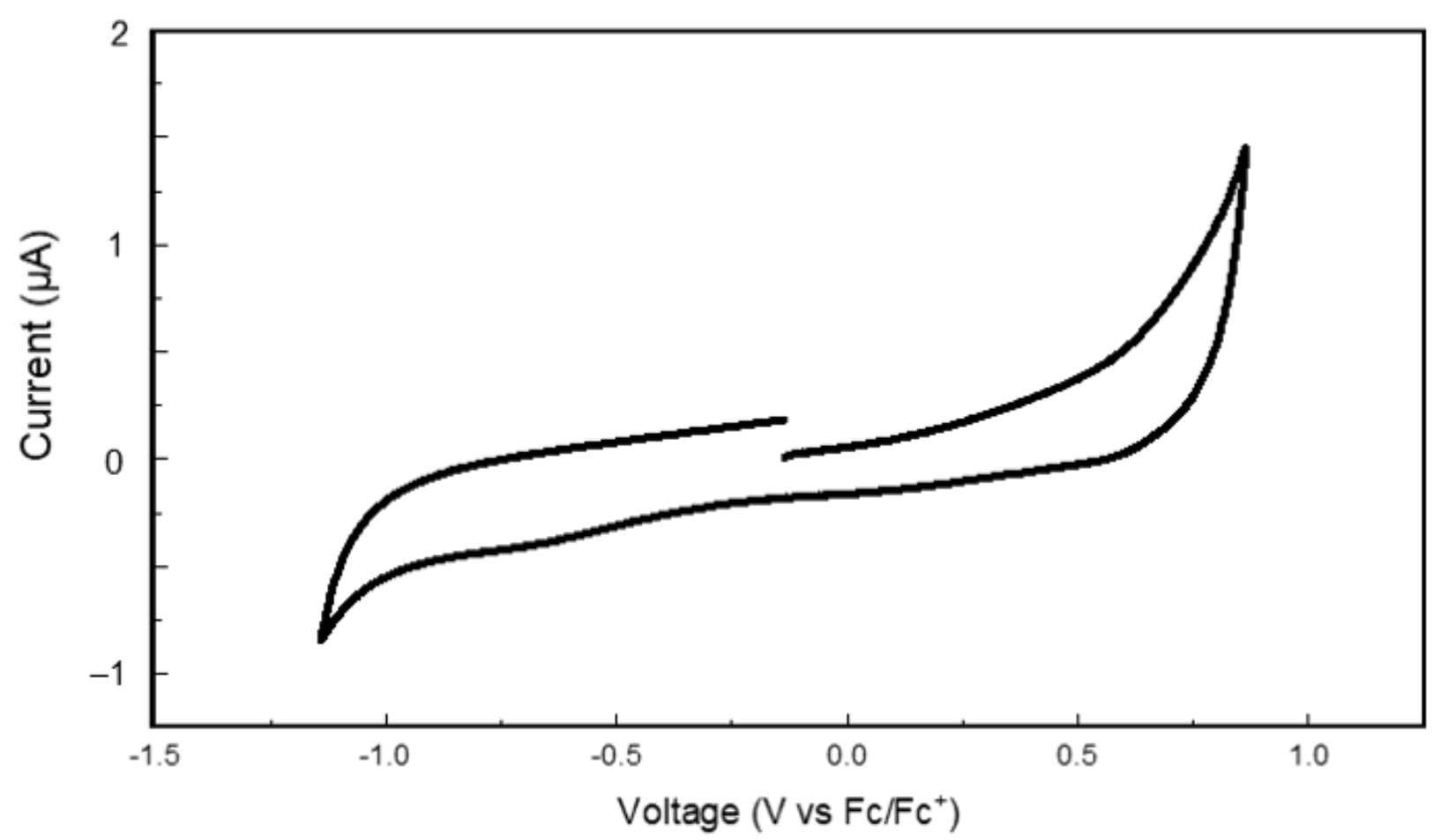

Fig. S30. Full-range cyclic voltammogram of the electrolyte (0.1 M n-Bu_4NPF_6 in MeCN) without analyte, recorded on the Pt working electrode. Scan rate = 100 mV/s.

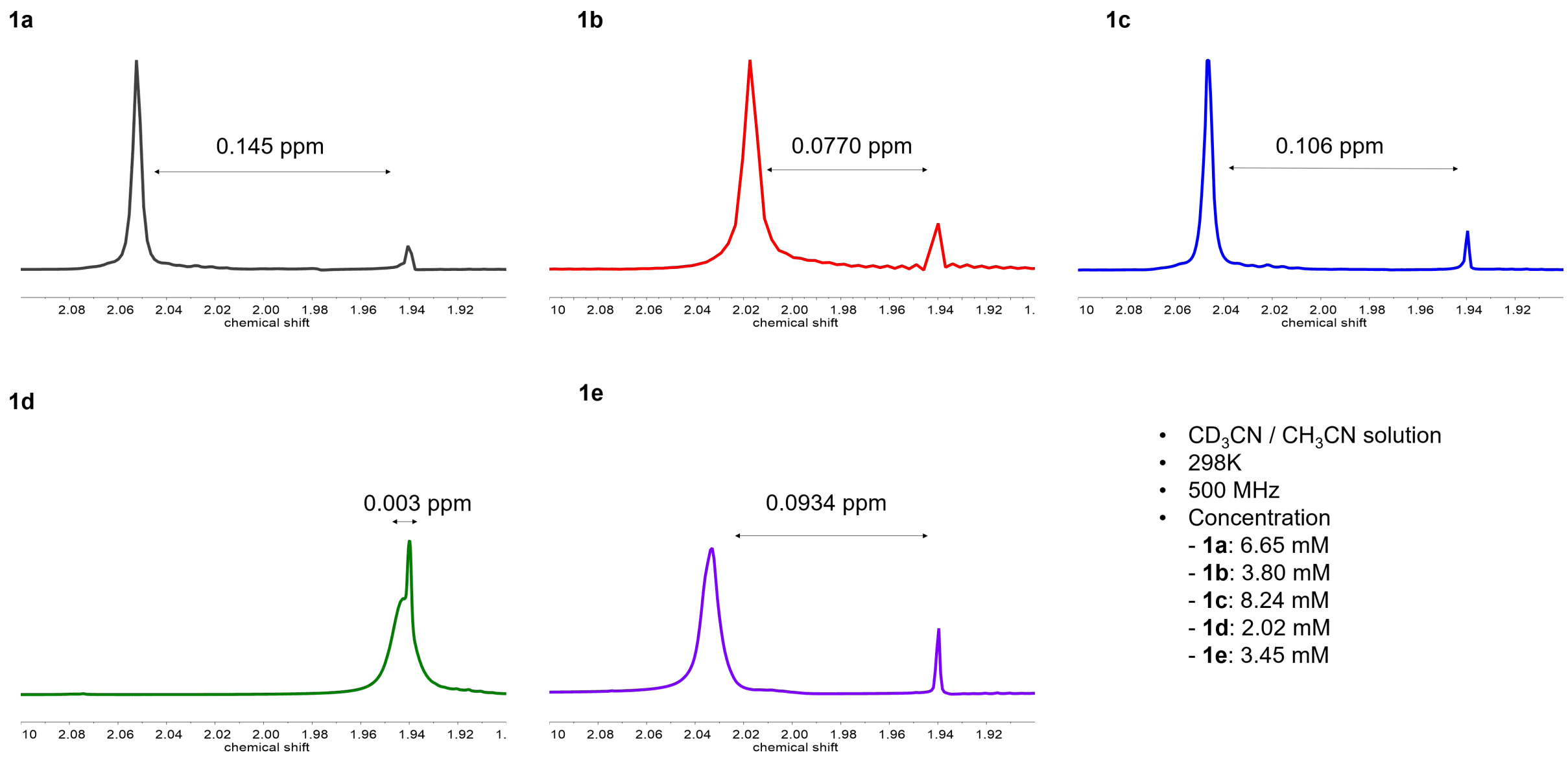

Fig. S31. ^{1}H NMR (500 MHz) spectra of **1a–e** in CD_3CN/CH_3CN = 1:50 (v/v) at T = 298 K, together with other parameters used for the Evans method analysis.

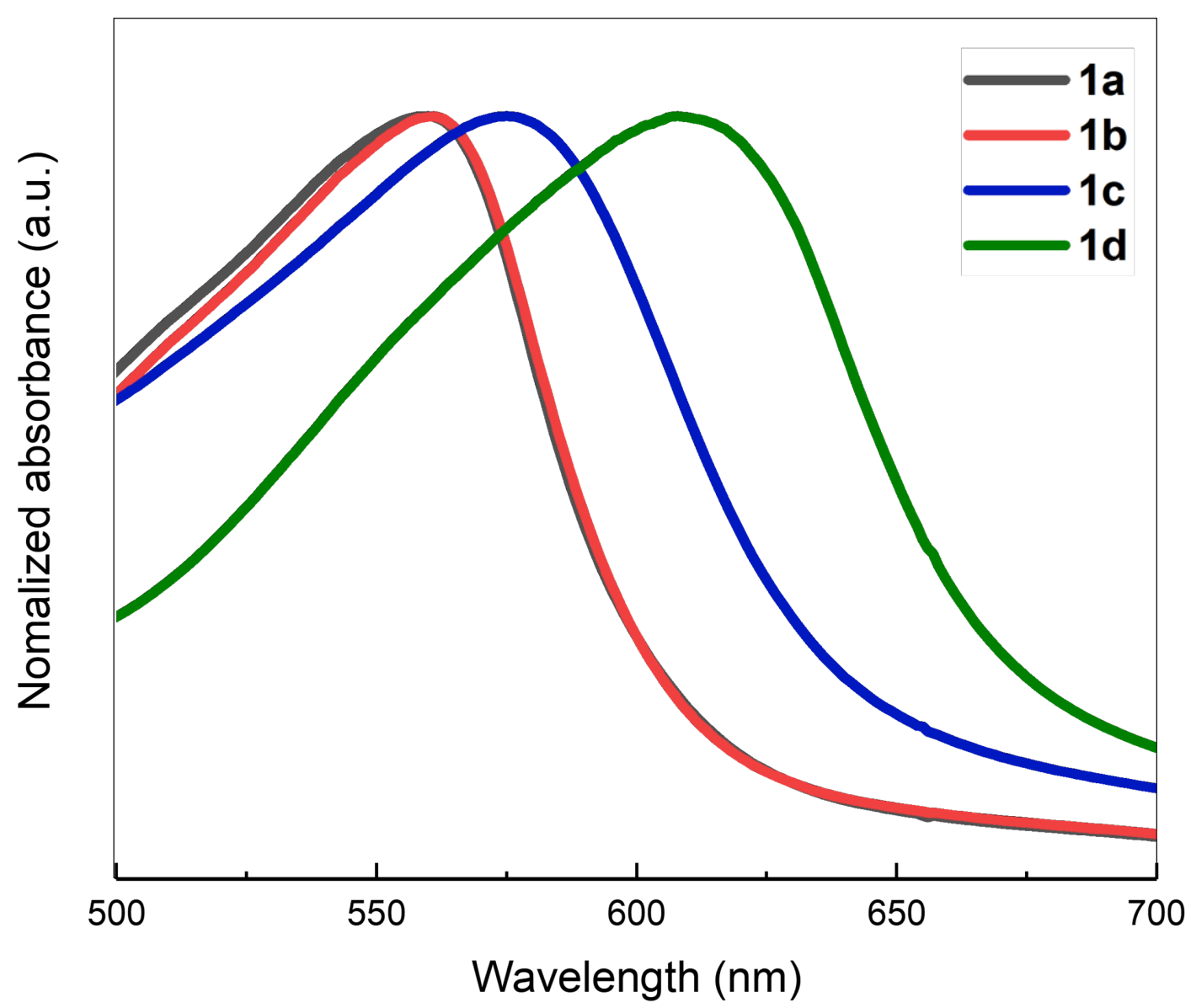

Fig. S32. UV–vis absorption spectra of **1a–d** in CH_3CN at 298 K.

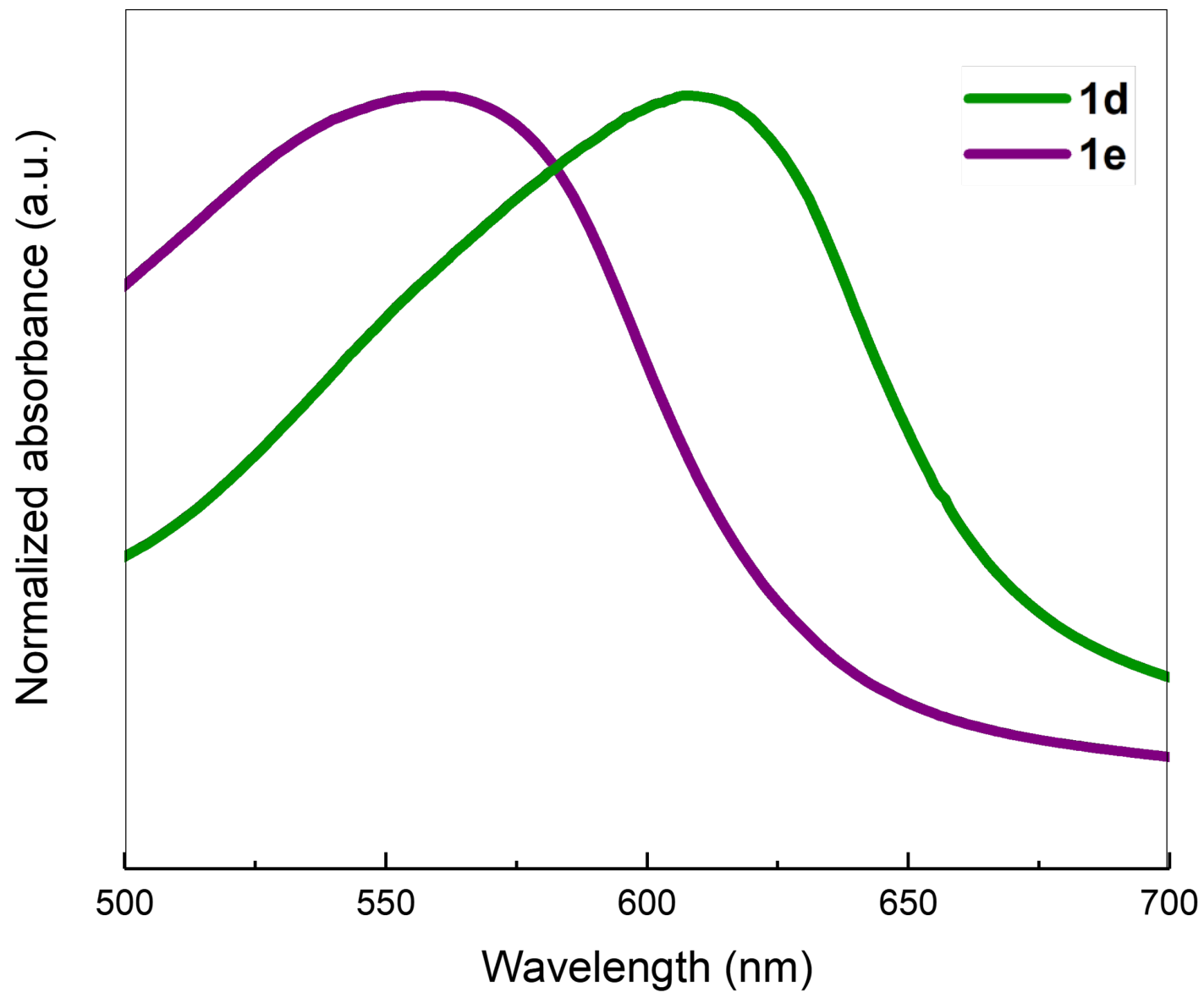

Fig. S33. UV–vis absorption spectra of **1d** and **1e** in CH_3CN at 298 K.

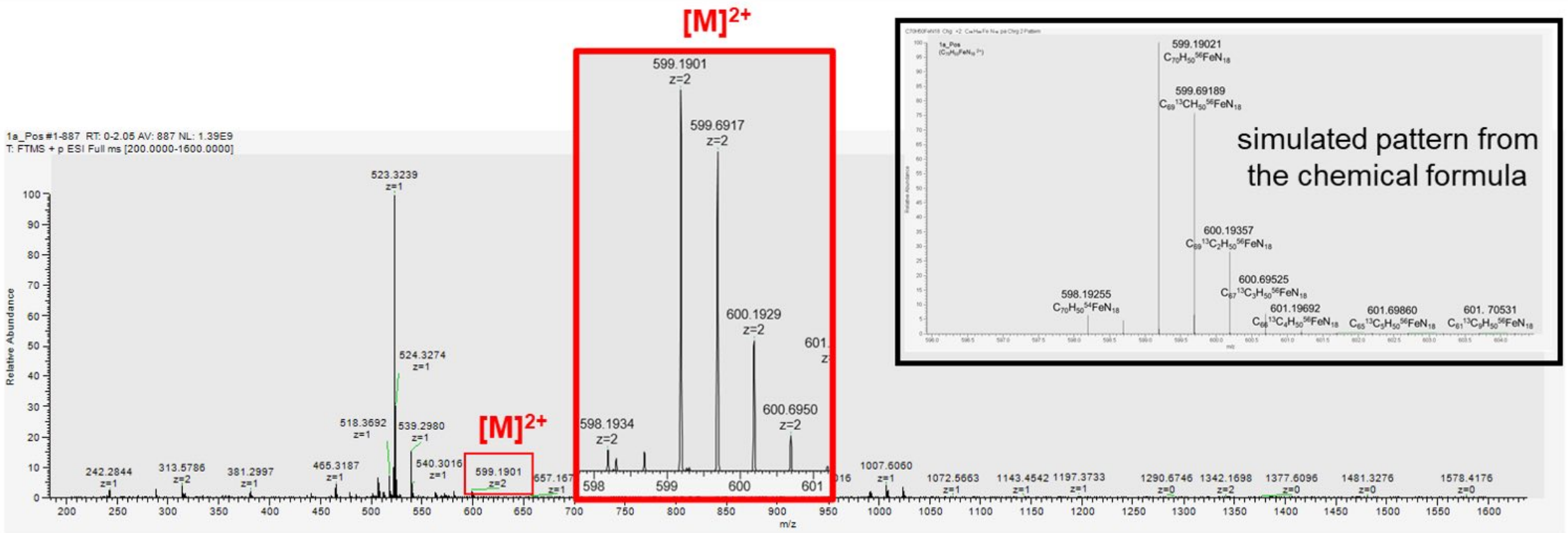

Fig. S34. Experimental and simulated high-resolution electrospray ionization mass spectrometry (HR-ESI-MS) spectra of **1a**.

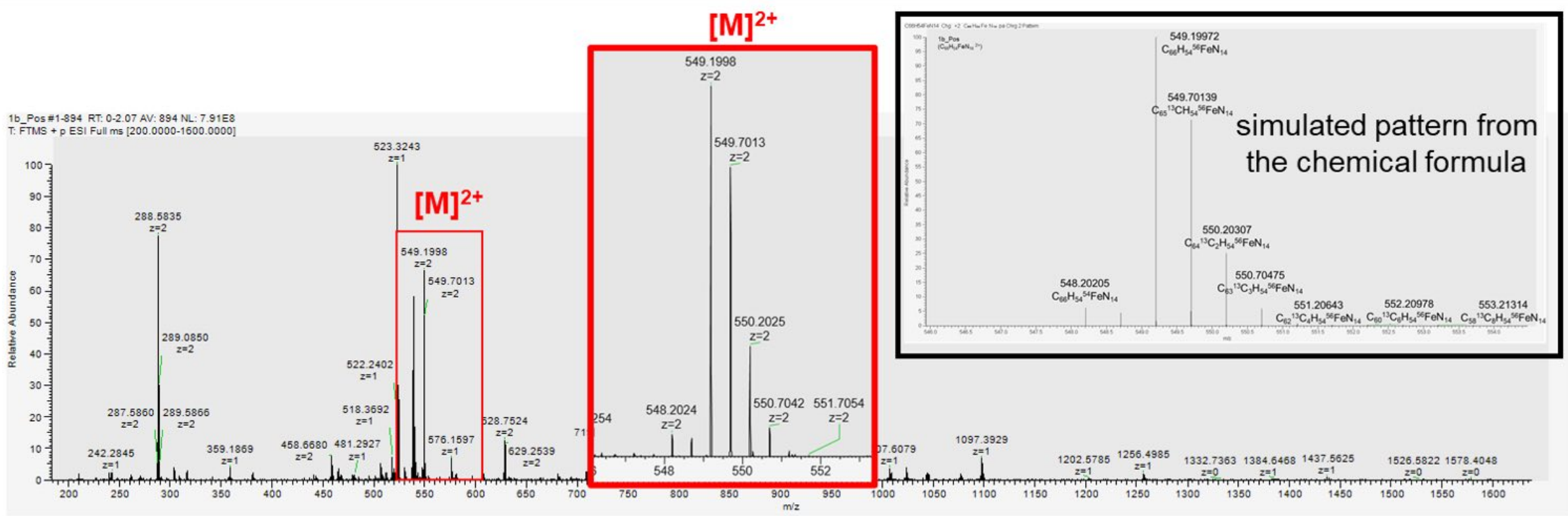

Fig. S35. Experimental and simulated HR-ESI-MS spectra of **1b**.

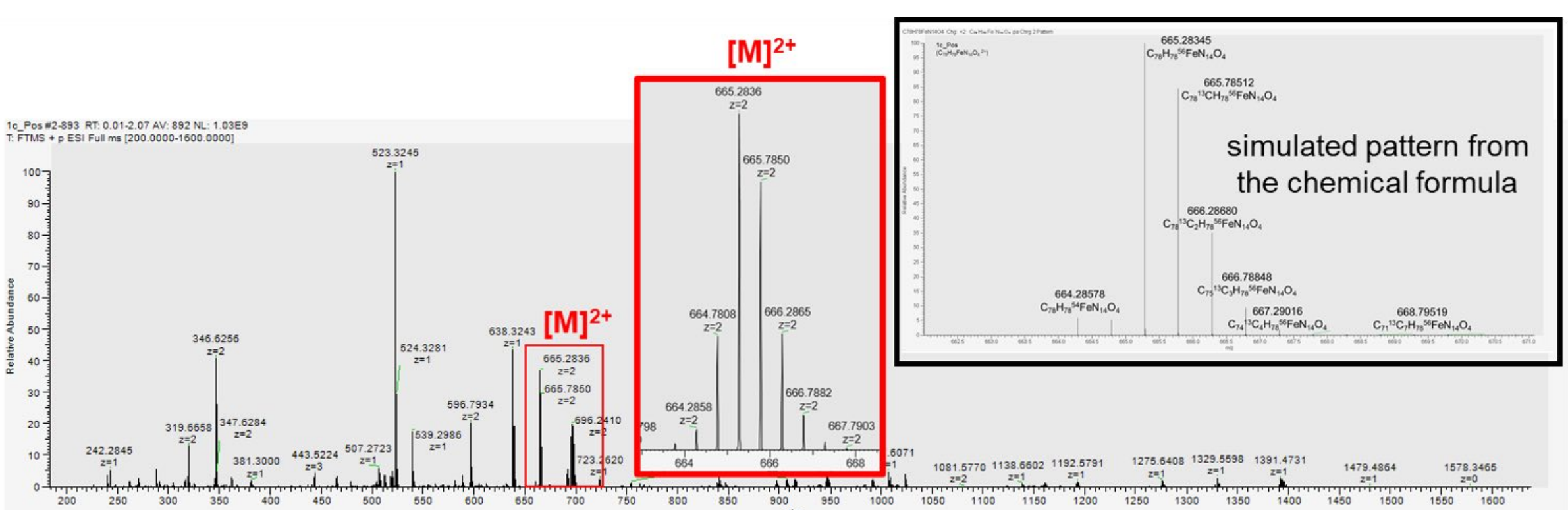

Fig. S36. Experimental and simulated HR-ESI-MS spectra of **1c**.

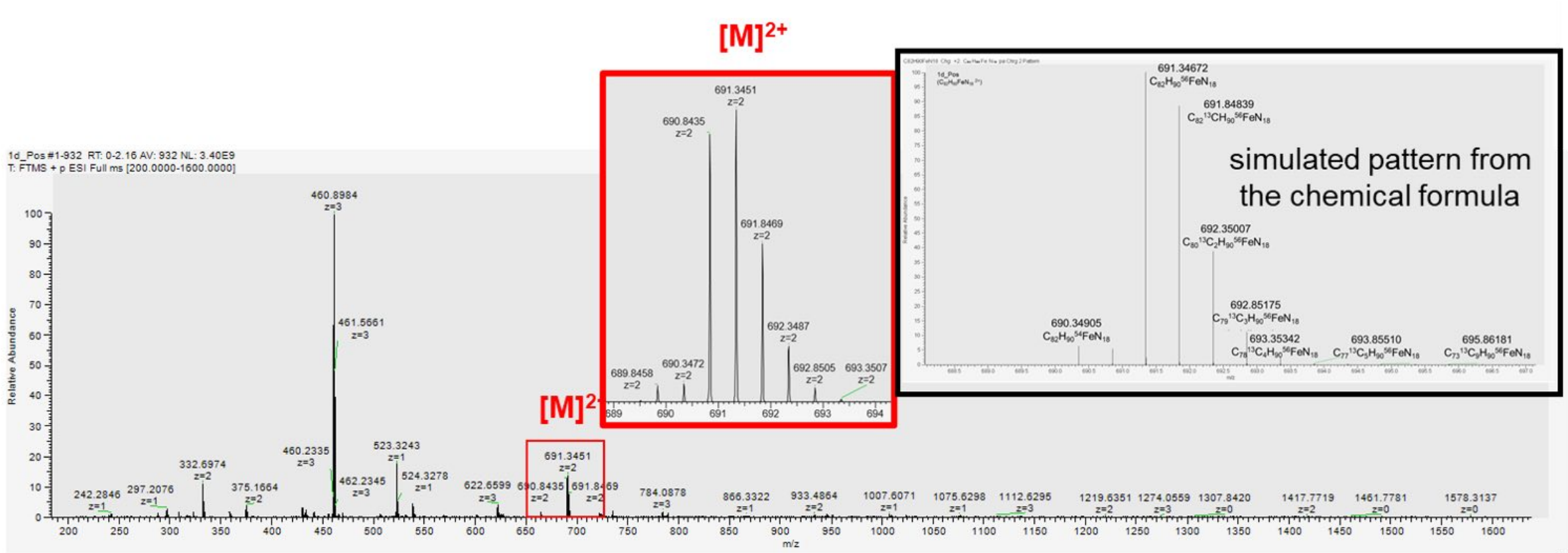

Fig. S37. Experimental and simulated HR-ESI-MS spectra of **1d**.

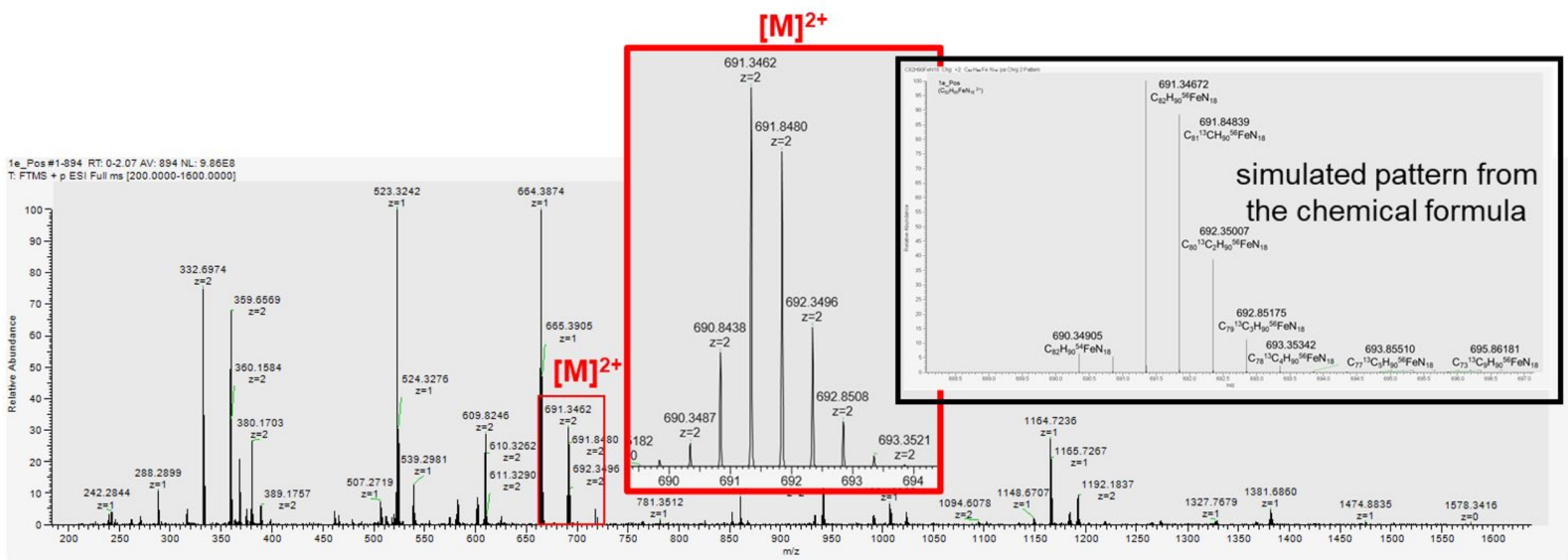

Fig. S38. Experimental and simulated HR-ESI-MS spectra of **1e**.

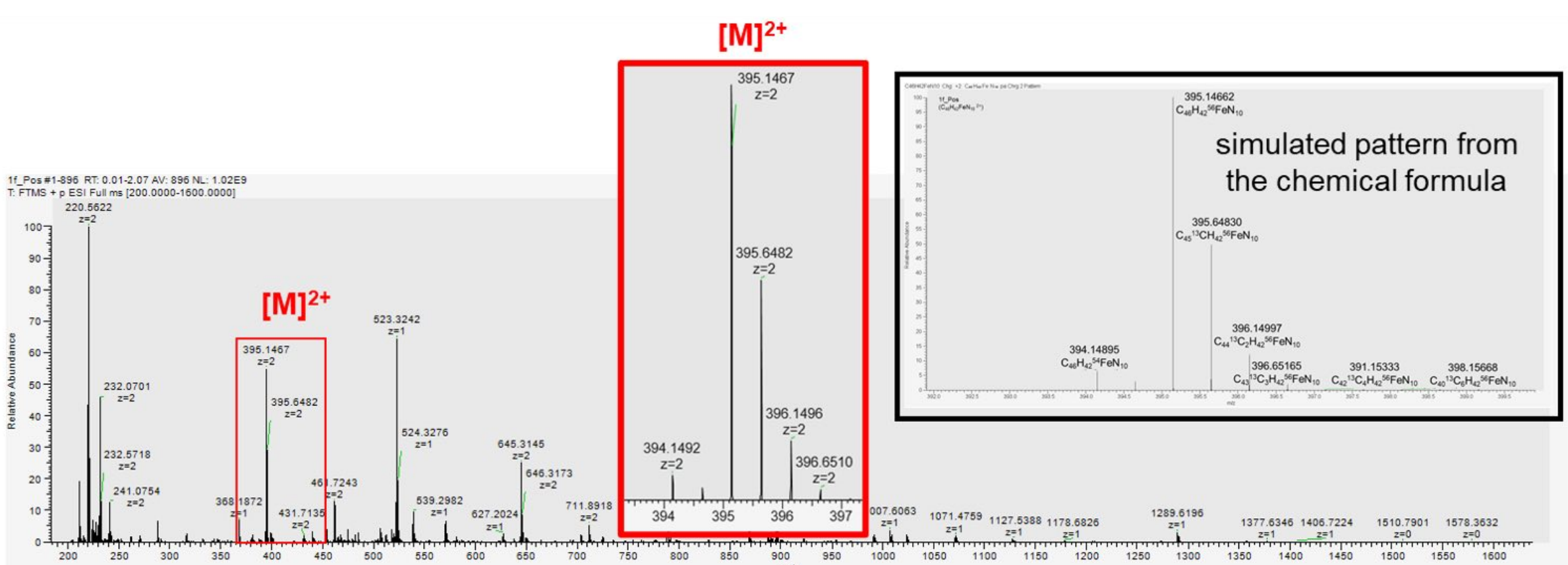

Fig. S39. Experimental and simulated high-resolution electrospray ionization mass spectrometry (HR-ESI-MS) spectra of **1f**.

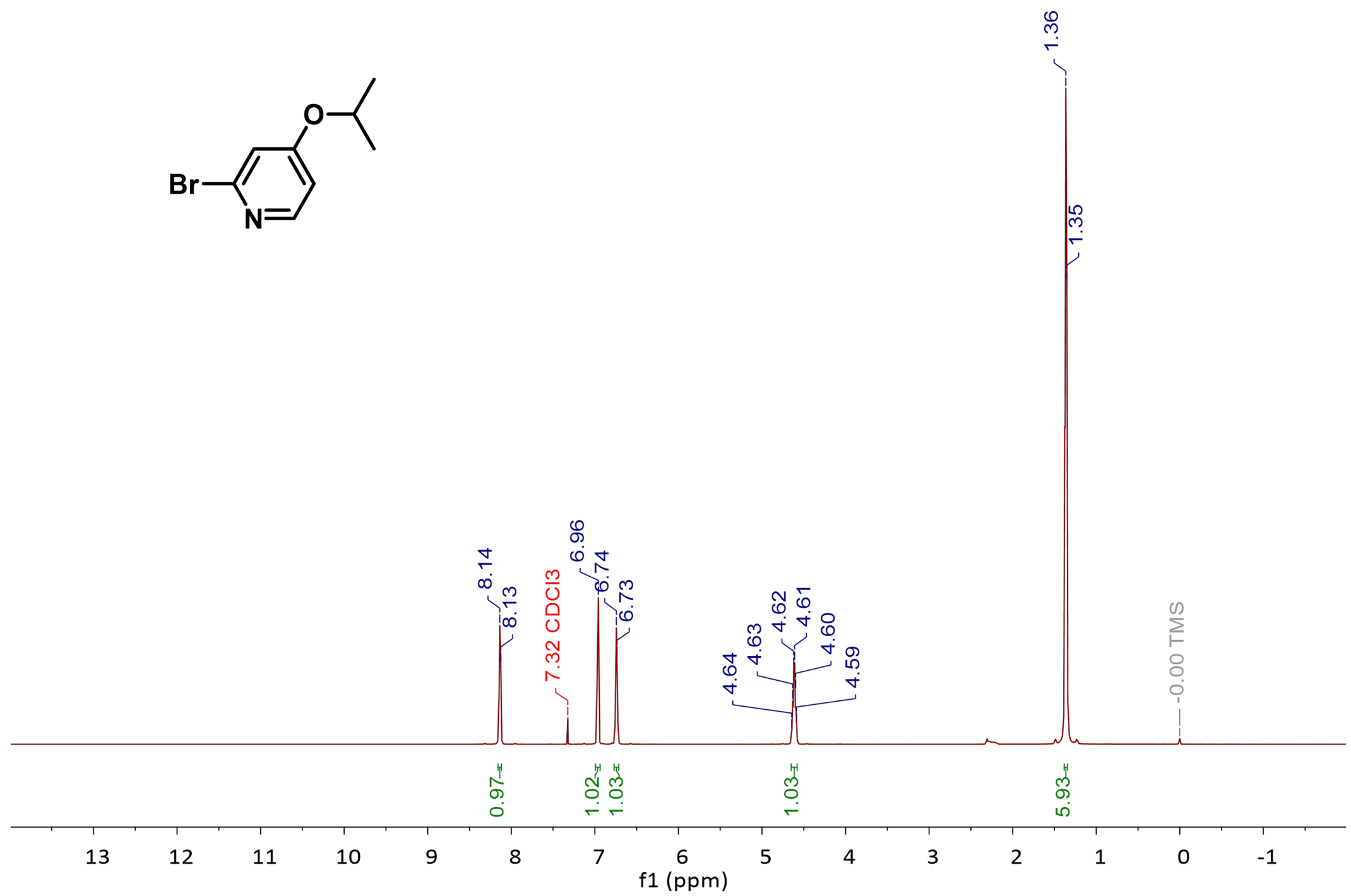

Fig. S40. ^{1}H NMR (500 MHz) spectrum of **6** in $CDCl_3$ (T = 298 K).

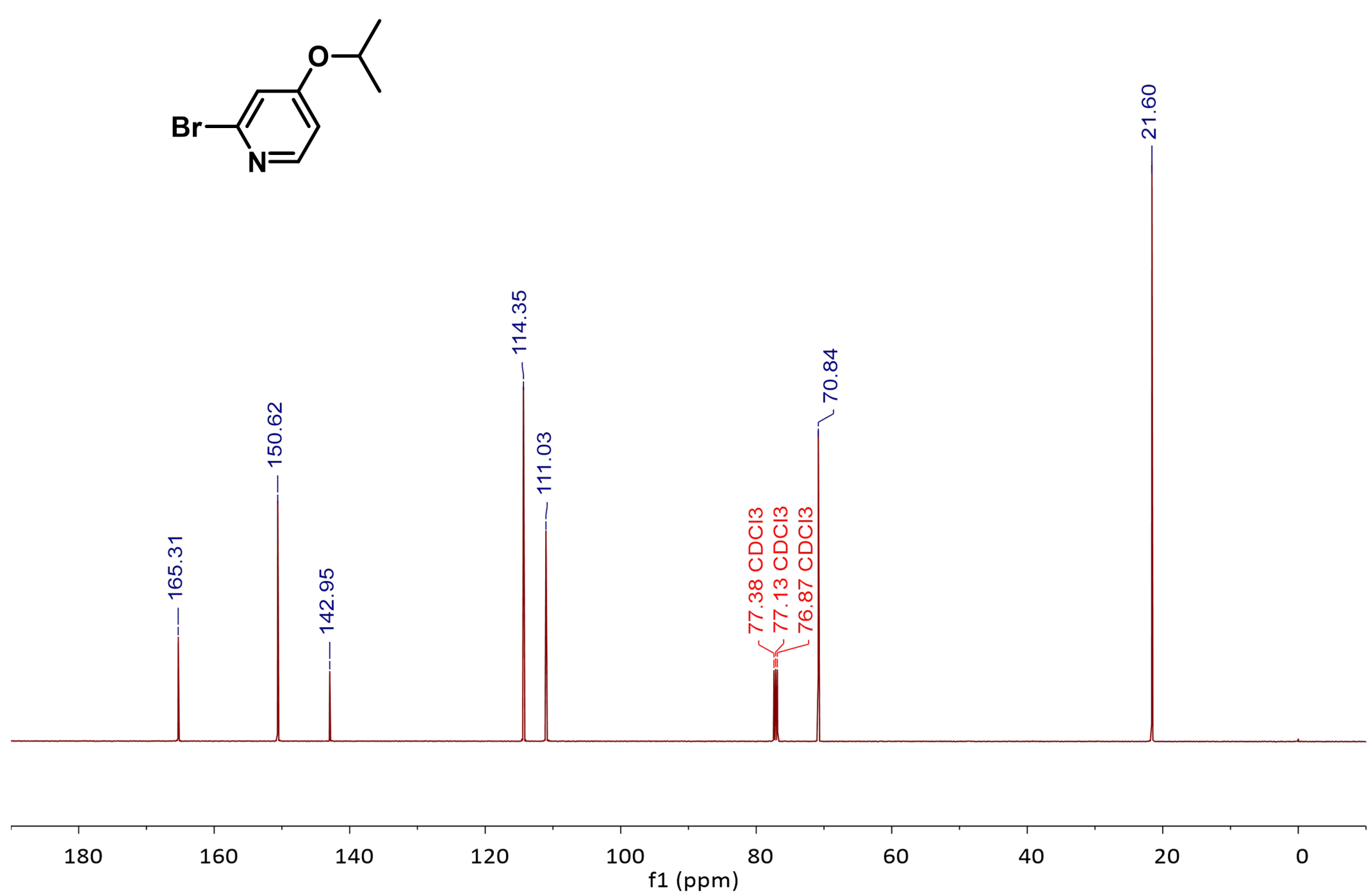

Fig. S41. ^{13}C NMR (125 MHz) spectrum of **6** in $CDCl_3$ (T = 298 K).

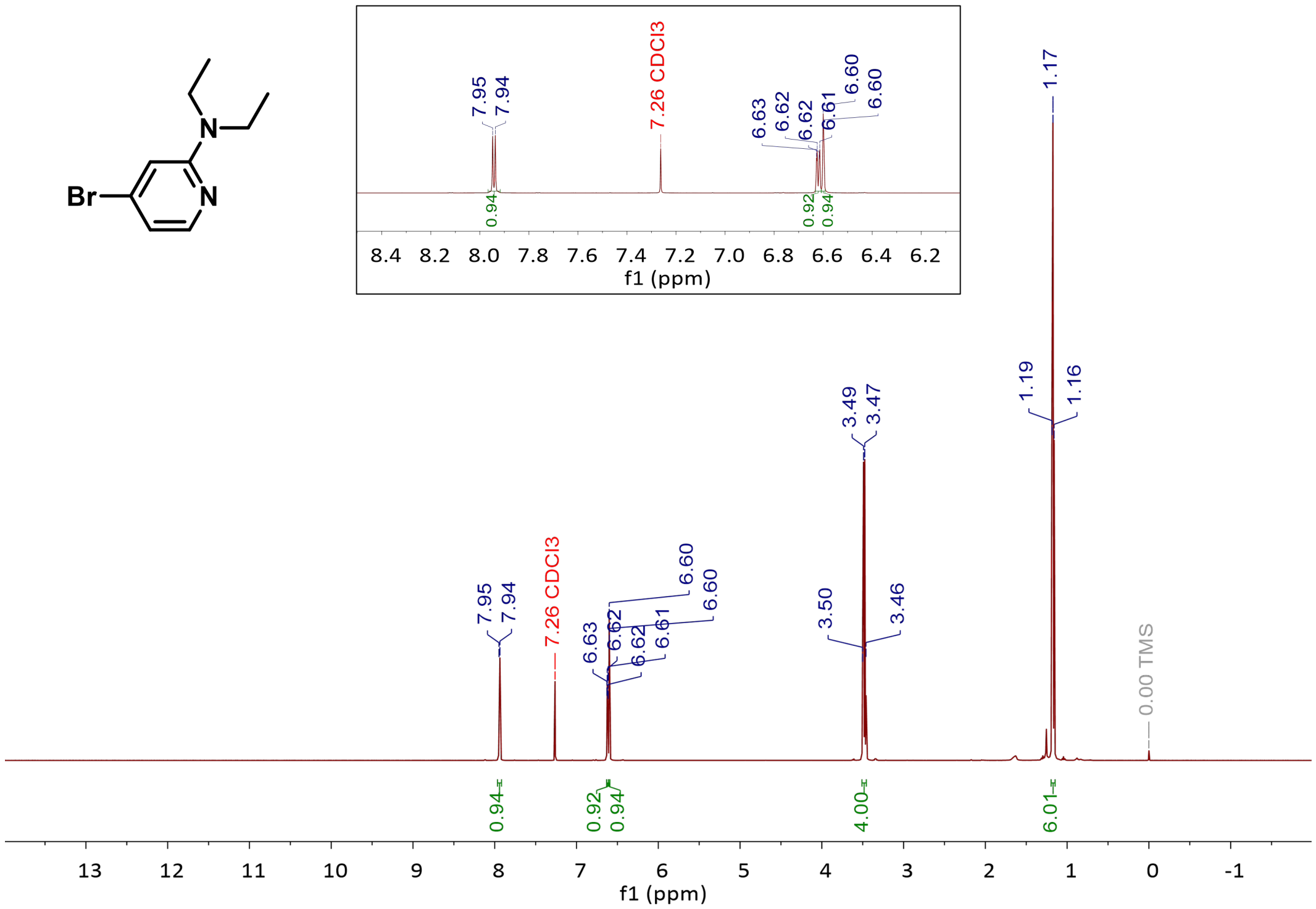

Fig. S42. ^{1}H NMR (500 MHz) spectrum of **7** in $CDCl_3$ (T = 298 K).

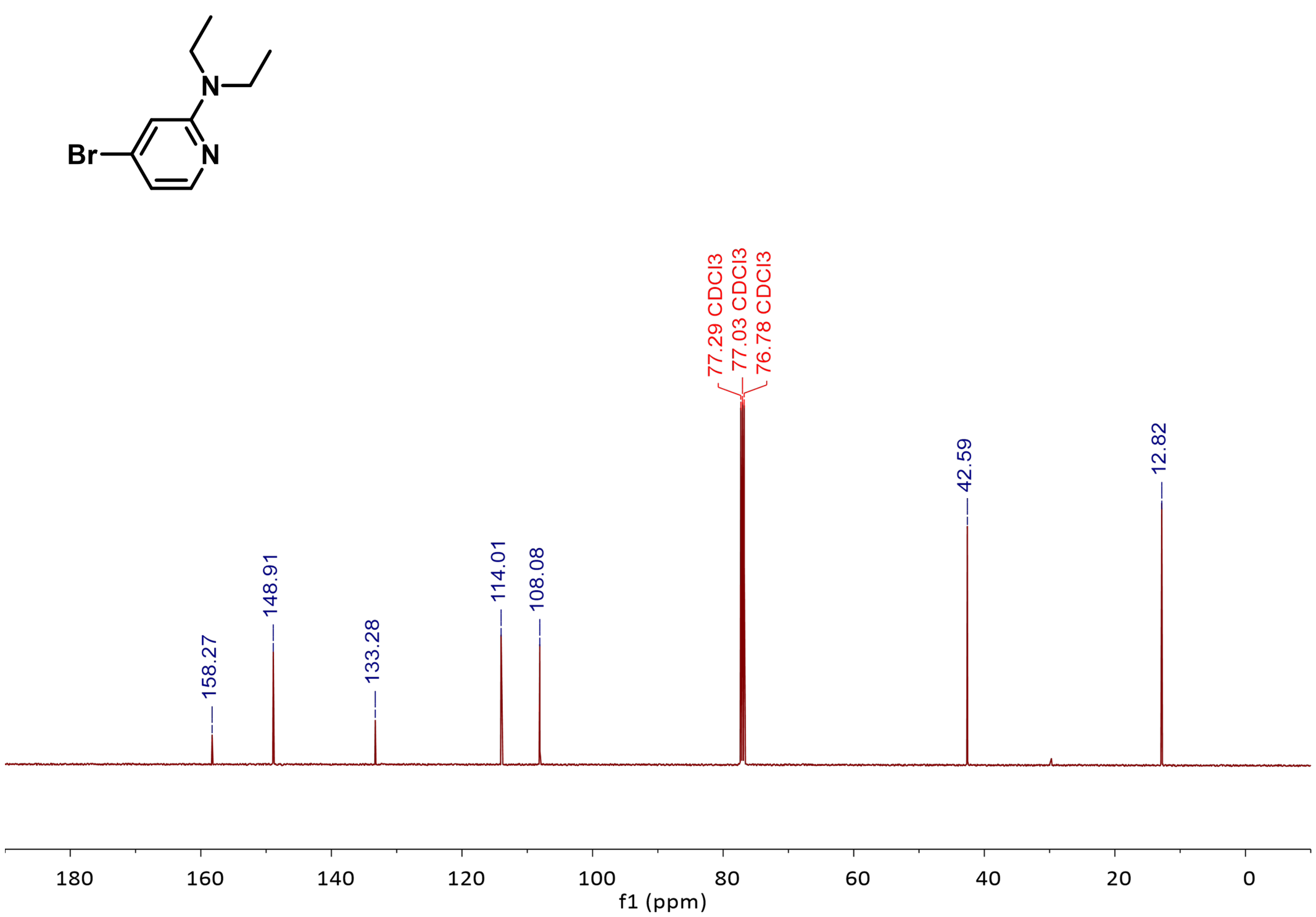

Fig. S43. ^{13}C NMR (125 MHz) spectrum of **7** in $CDCl_3$ (T = 298 K).

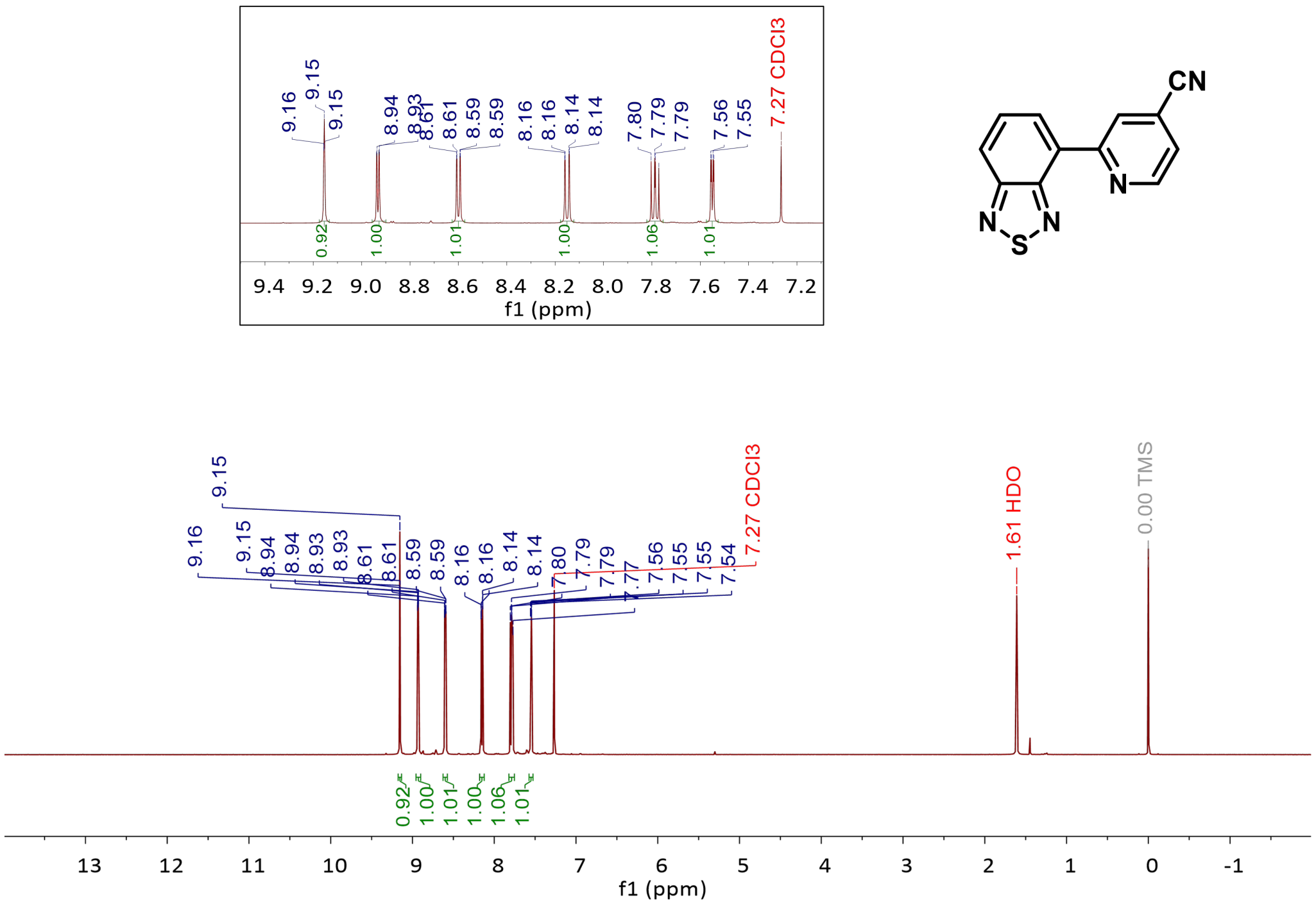

Fig. S44. ^{1}H NMR (500 MHz) spectrum of **3a** in $CDCl_3$ (T = 298 K).

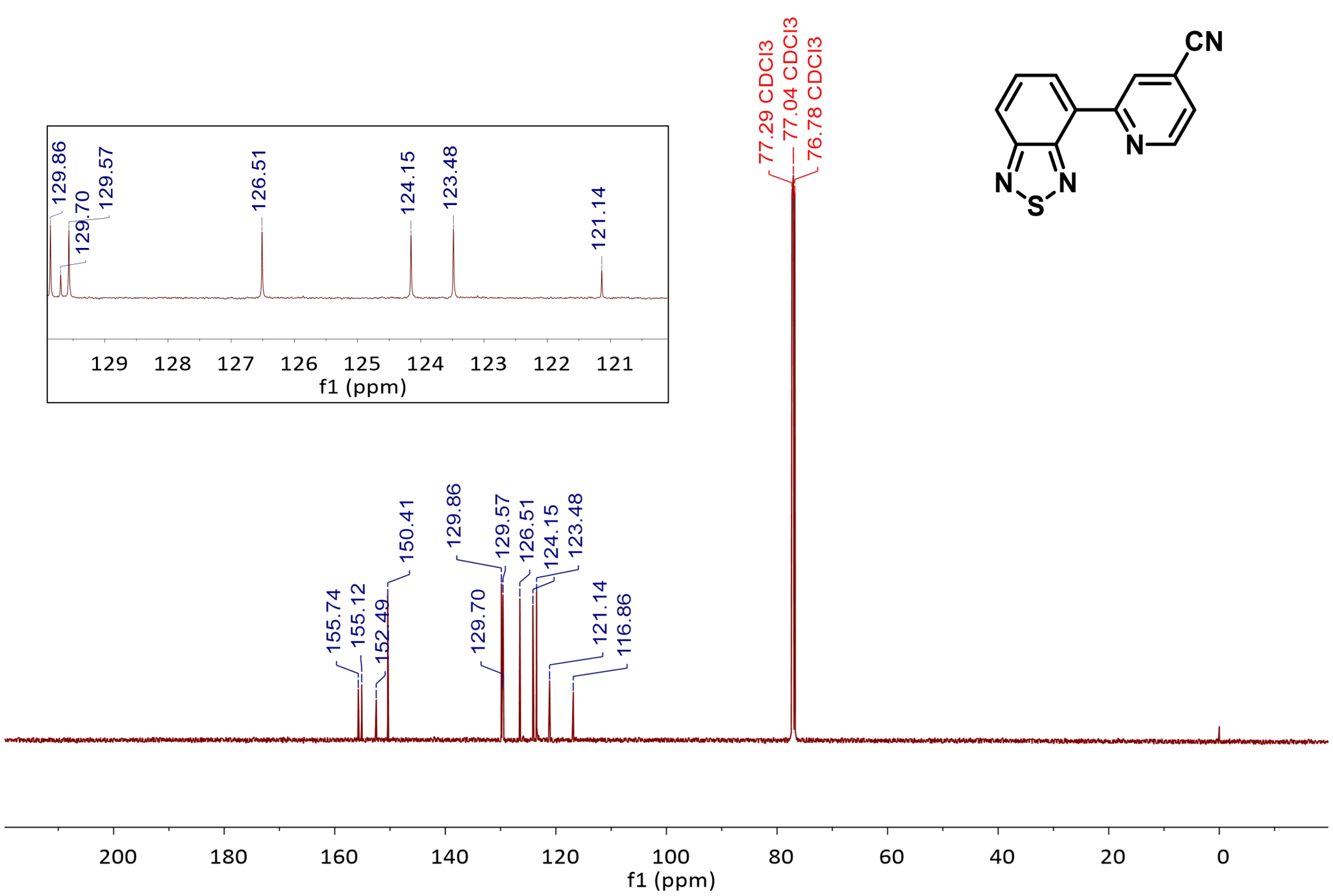

Fig. S45. ^{13}C NMR (125 MHz) spectrum of **3a** in $CDCl_3$ (T = 298 K).

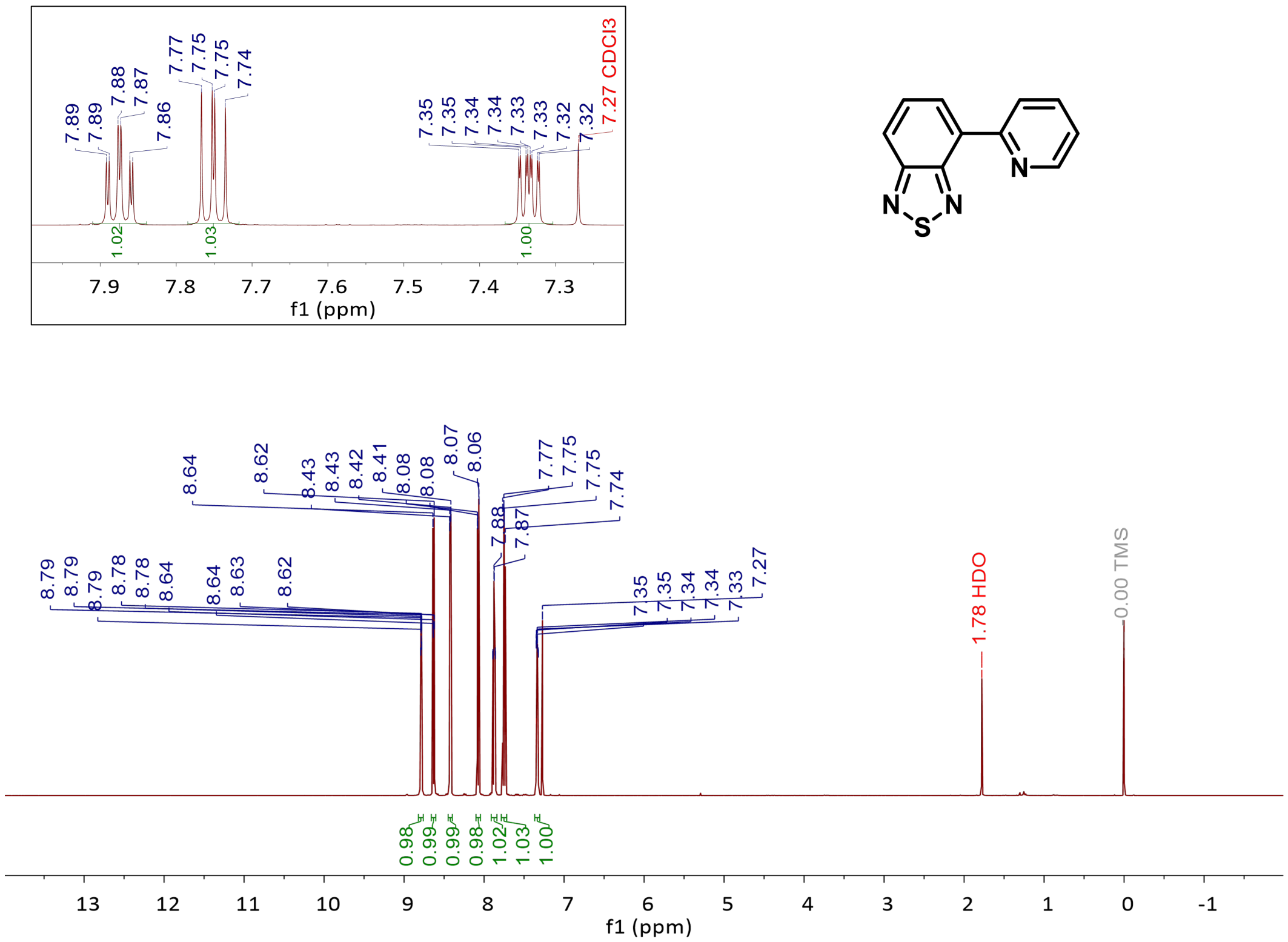

Fig. S46. ^{1}H NMR (500 MHz) spectrum of **3b** in $CDCl_3$ (T = 298 K).

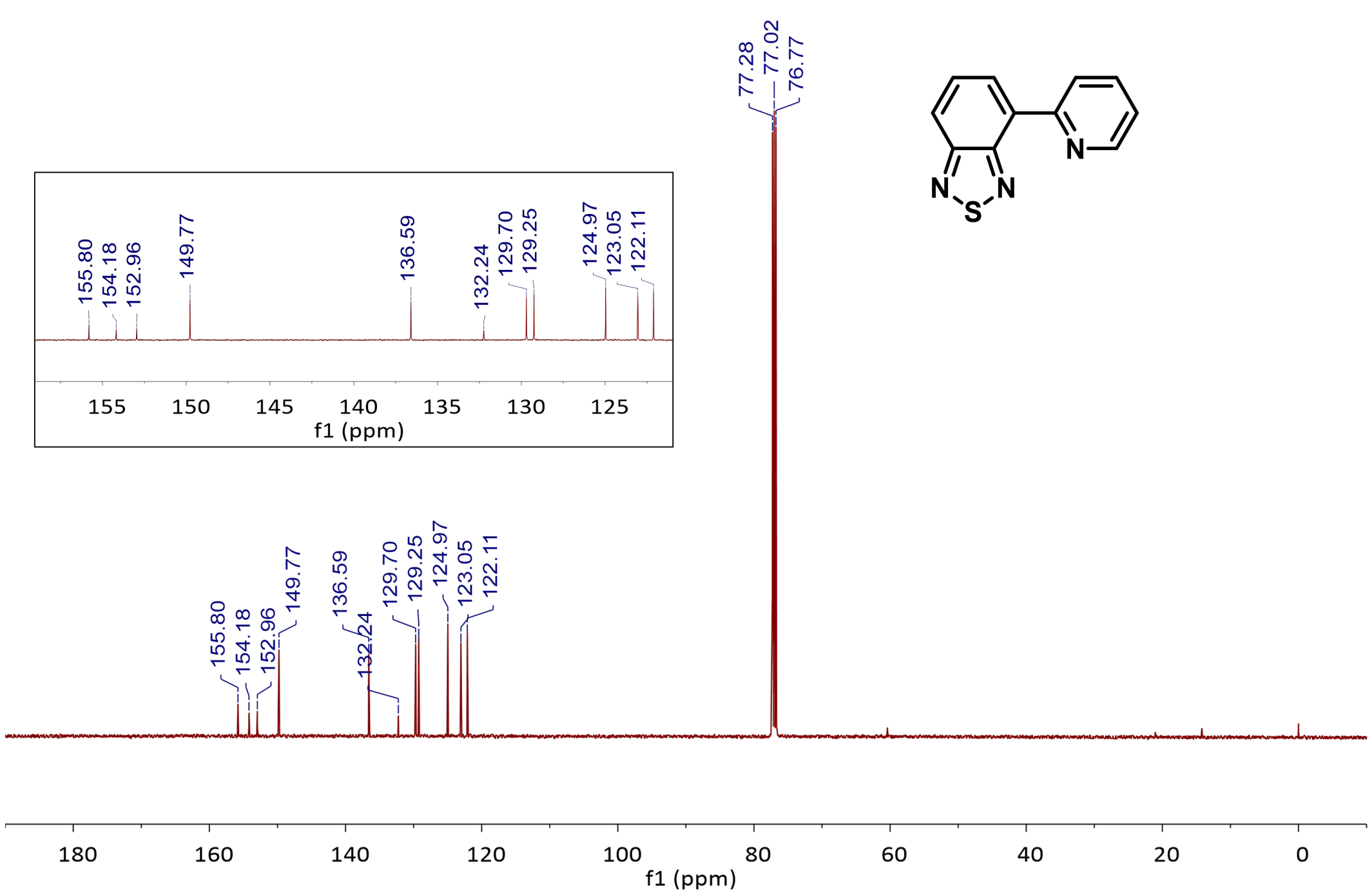

Fig. S47. ^{13}C NMR (125 MHz) spectrum of **3b** in $CDCl_3$ (T = 298 K).

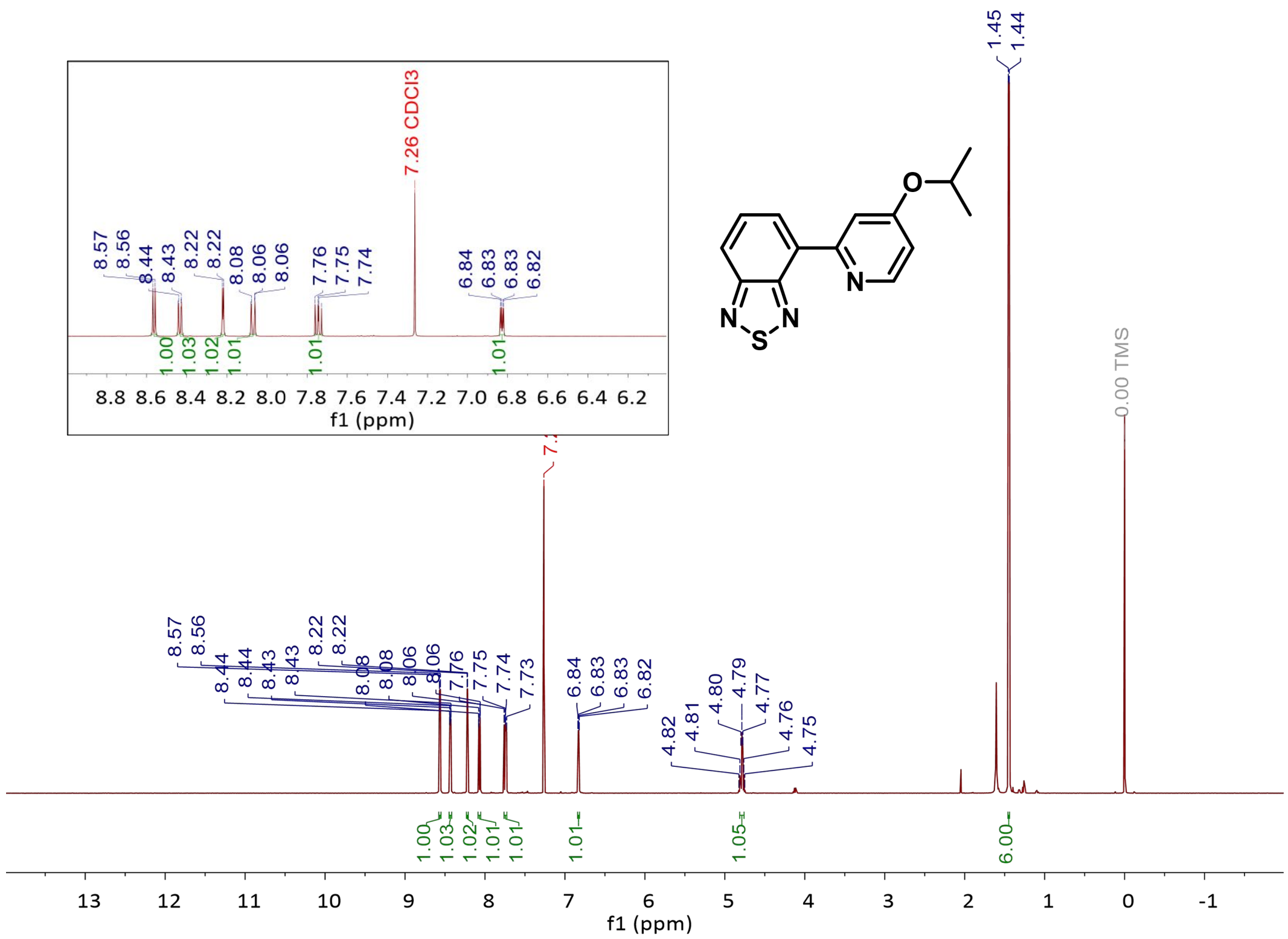

Fig. S48. ^{1}H NMR (500 MHz) spectrum of **3c** in $CDCl_3$ (T = 298 K).

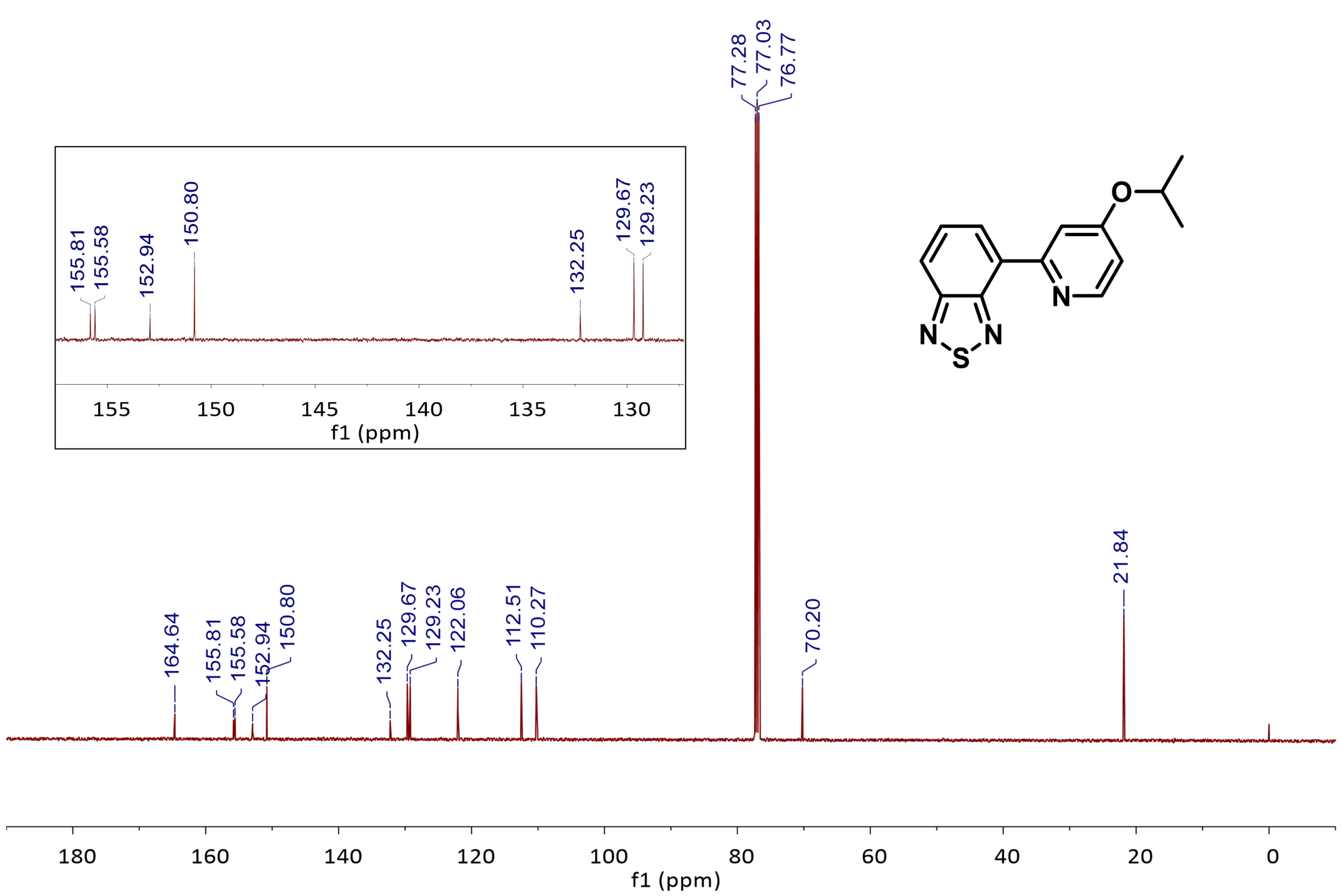

Fig. S49. ^{13}C NMR (125 MHz) spectrum of **3c** in $CDCl_3$ (T = 298 K).

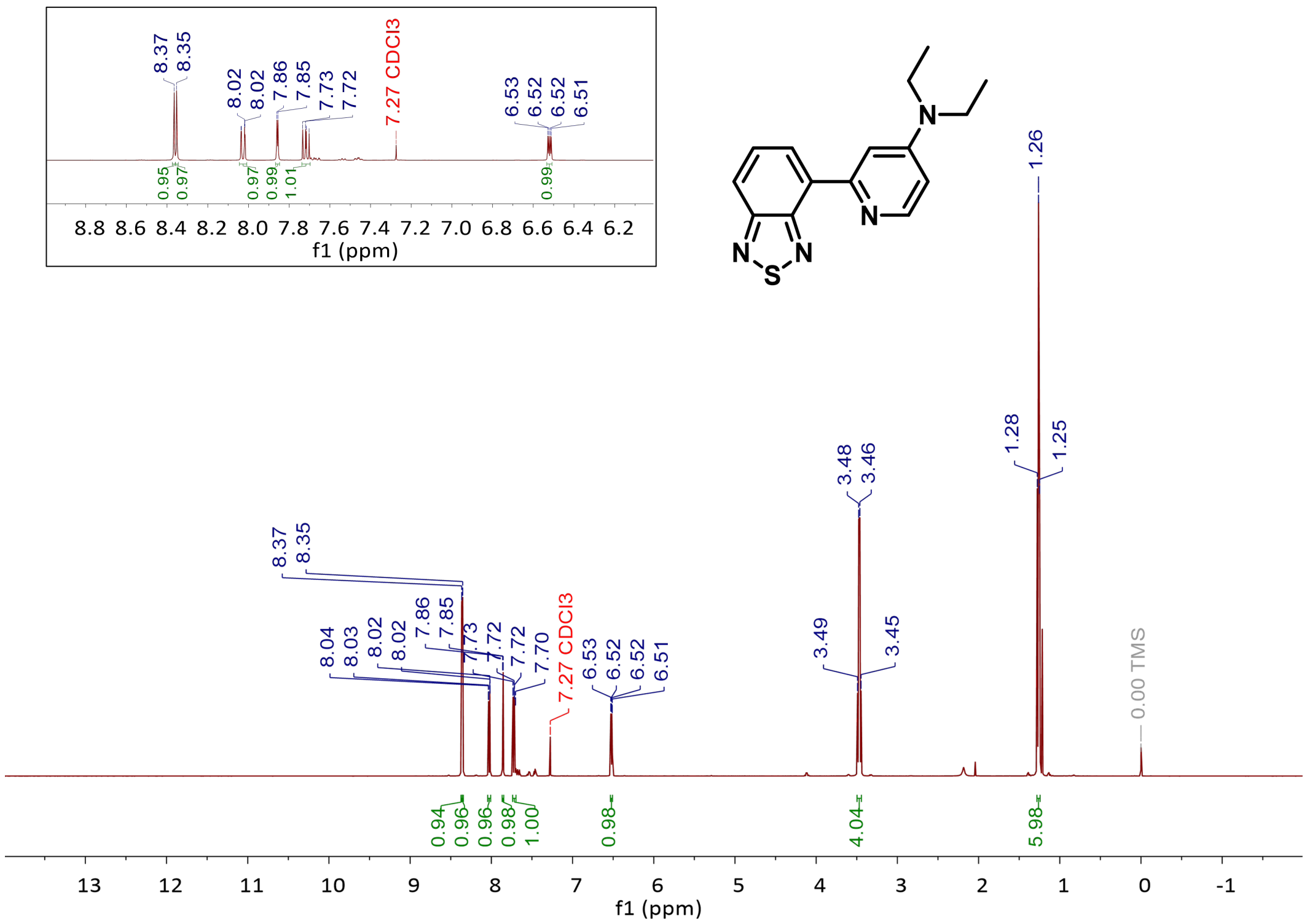

Fig. S50. ^{1}H NMR (500 MHz) spectrum of **3d** in $CDCl_3$ (T = 298 K).

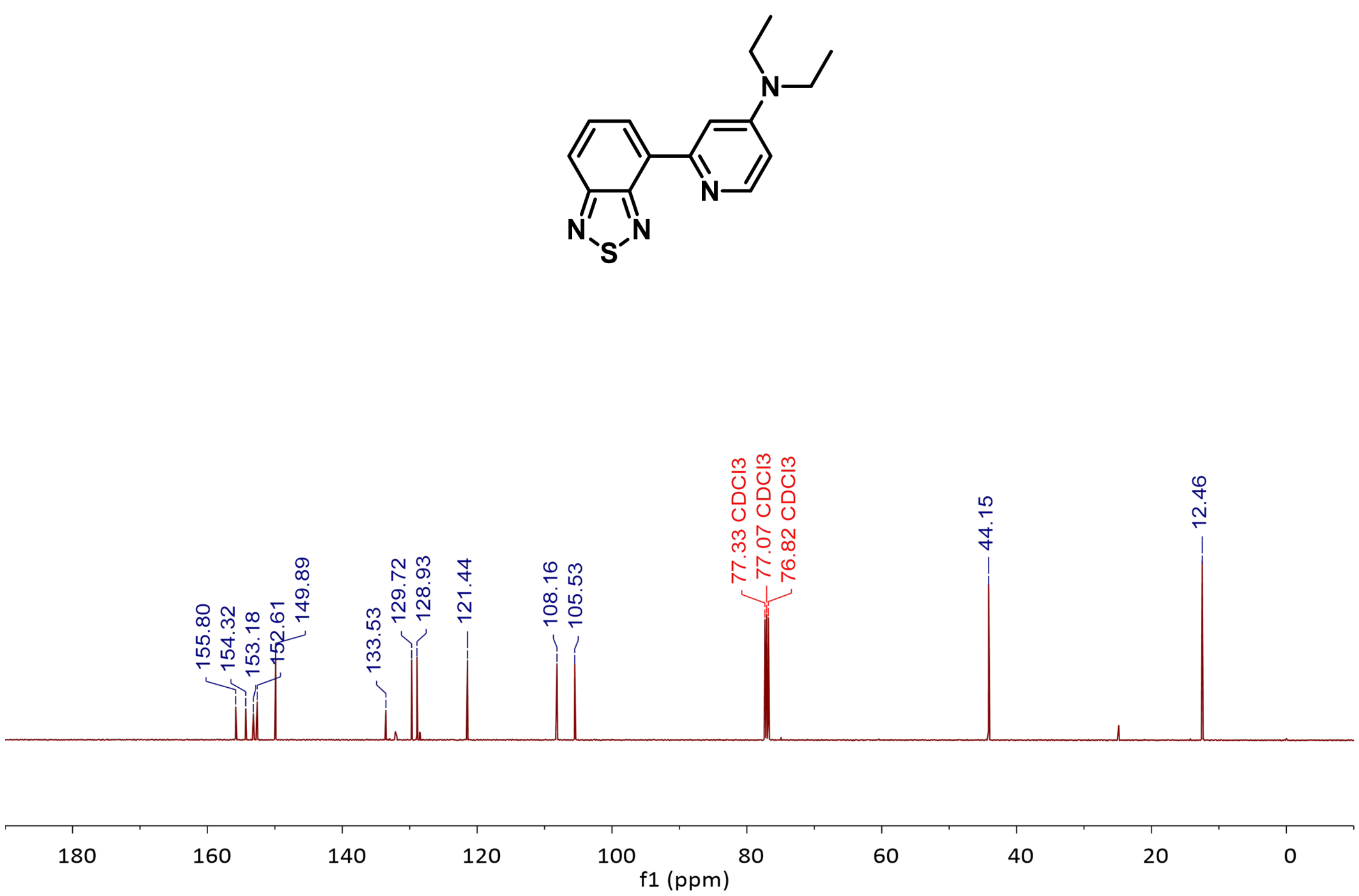

Fig. S51. ^{13}C NMR (125 MHz) spectrum of **3d** in $CDCl_3$ (T = 298 K).

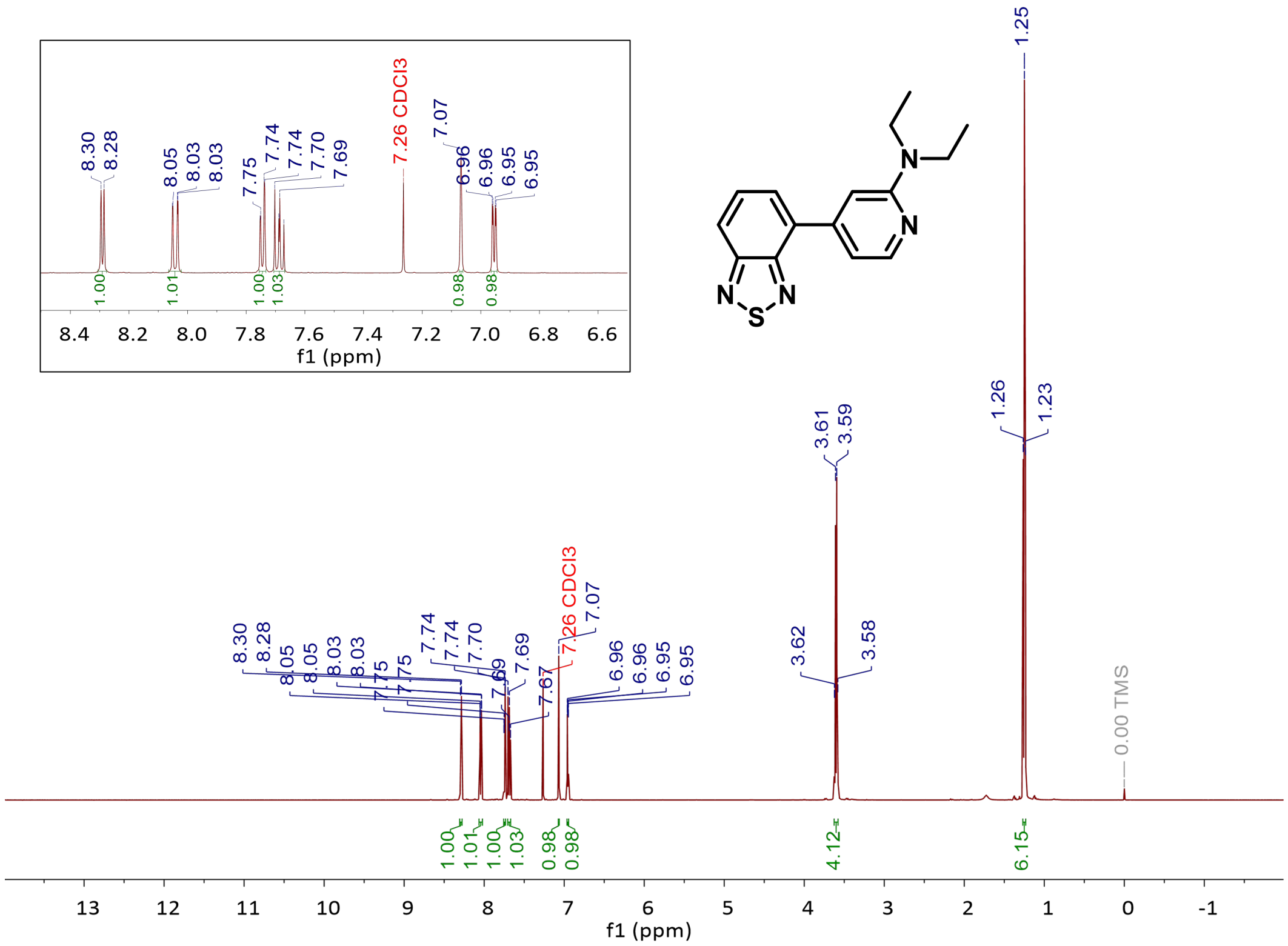

Fig. S52. ^{1}H NMR (500 MHz) spectrum of **3e** in $CDCl_3$ (T = 298 K).

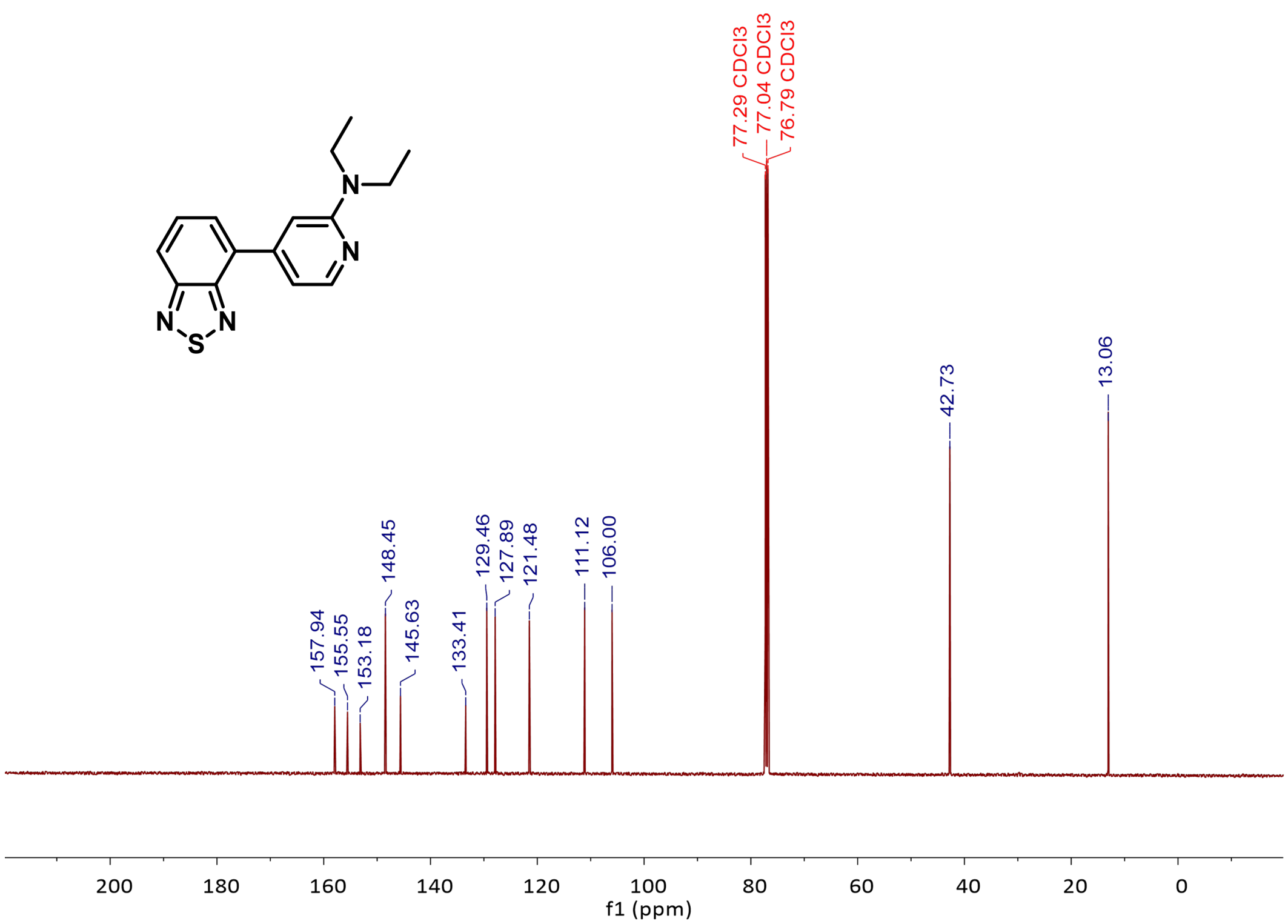

Fig. S53. ^{13}C NMR (125 MHz) spectrum of **3e** in $CDCl_3$ (T = 298 K).

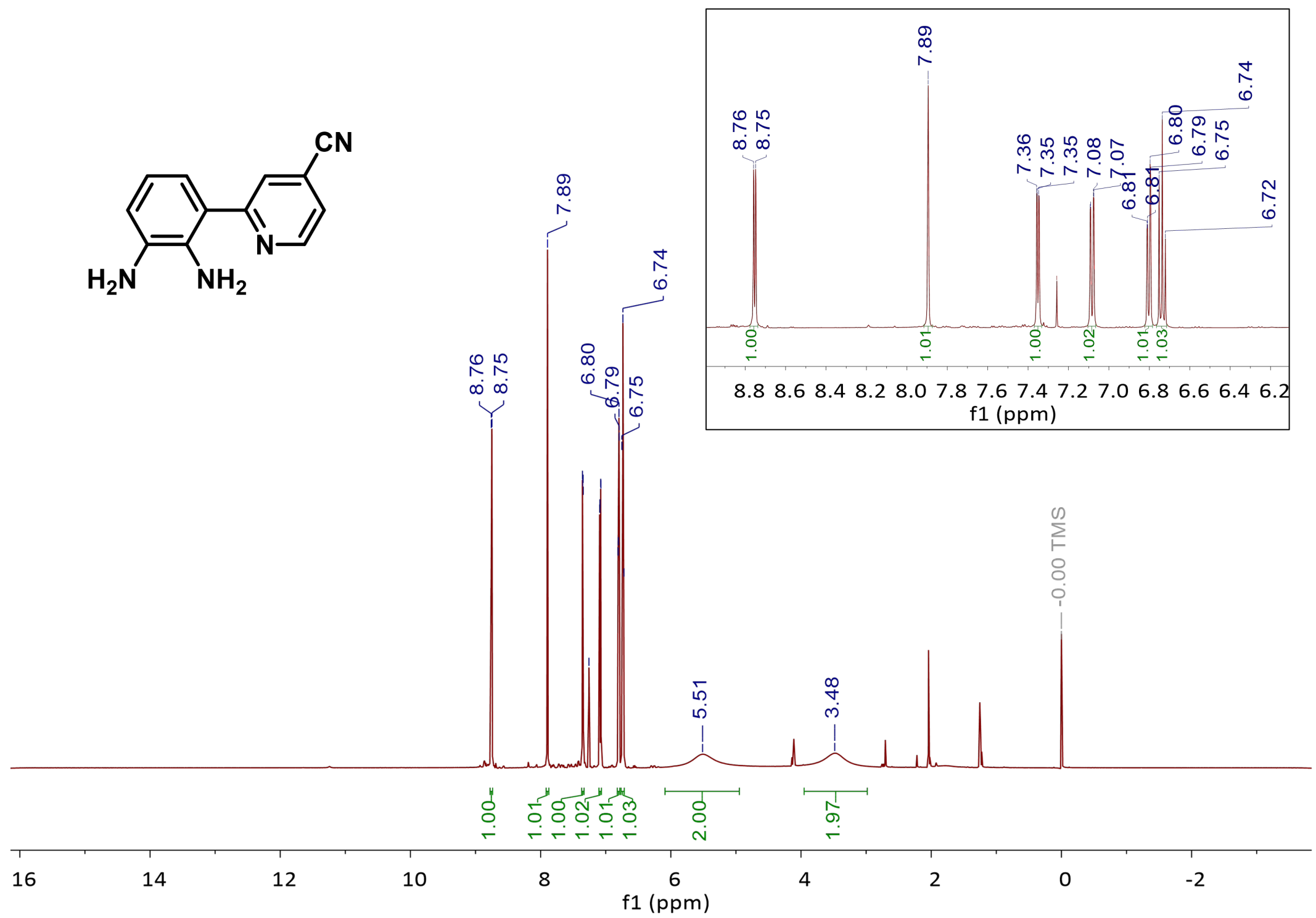

Fig. S54. ^{1}H NMR (500 MHz) spectrum of **4a** in $CDCl_3$ (T = 298 K).

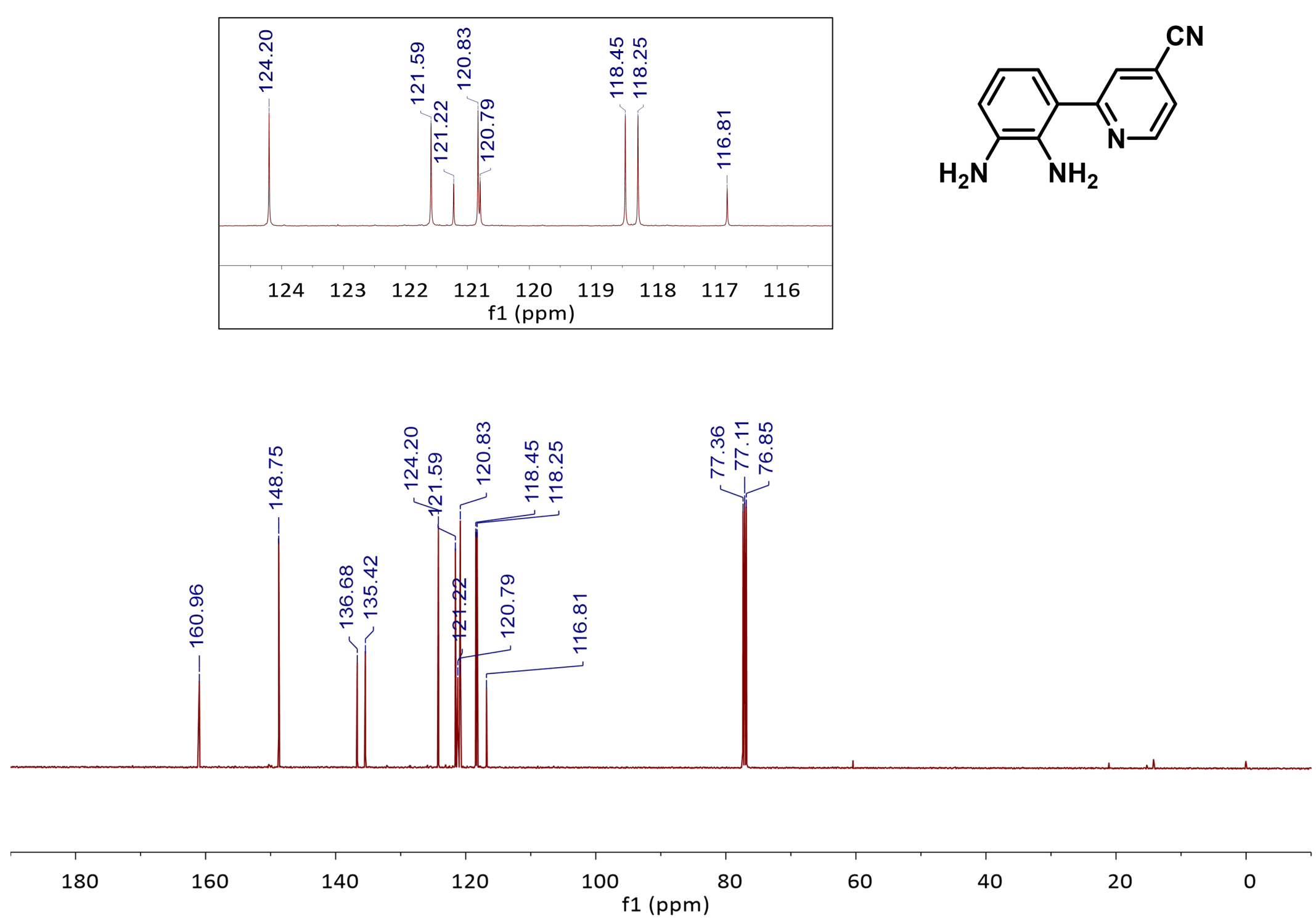

Fig. S55. ^{13}C NMR (125 MHz) spectrum of **4a** in $CDCl_3$ (T = 298 K).

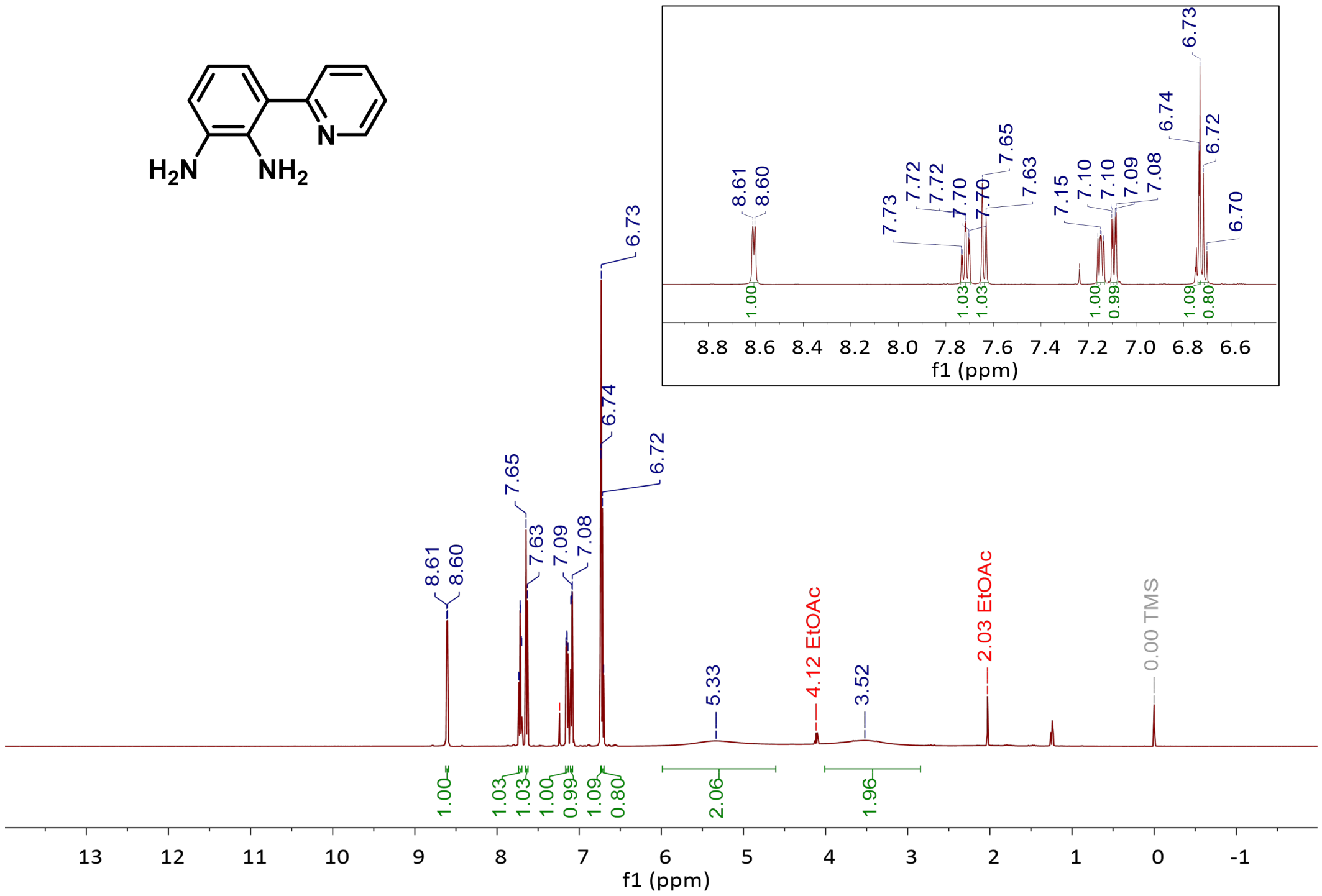

Fig. S56. ^{1}H NMR (500 MHz) spectrum of **4b** in $CDCl_3$ (T = 298 K).

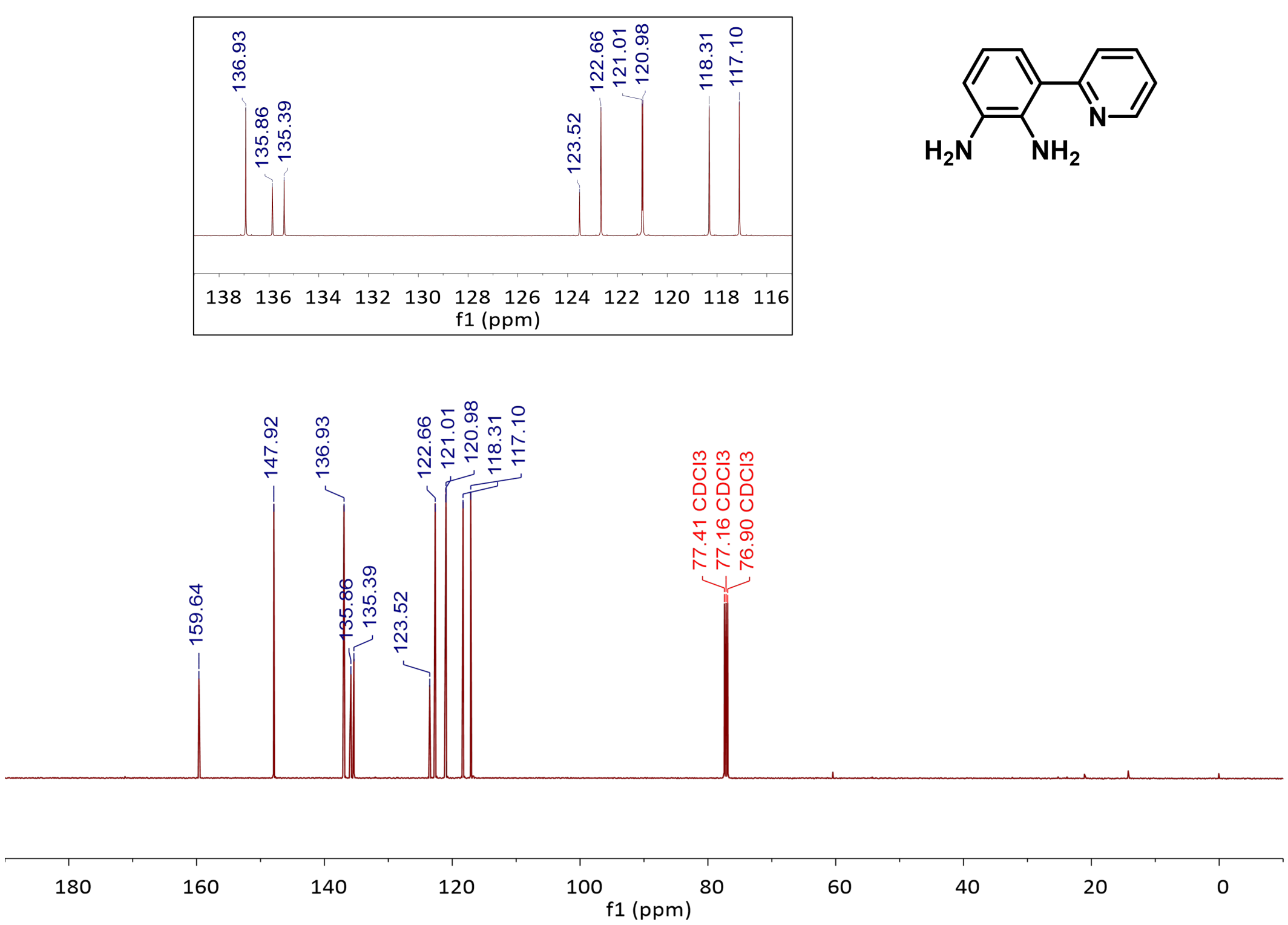

Fig. S57. ^{13}C NMR (125 MHz) spectrum of **4b** in $CDCl_3$ (T = 298 K).

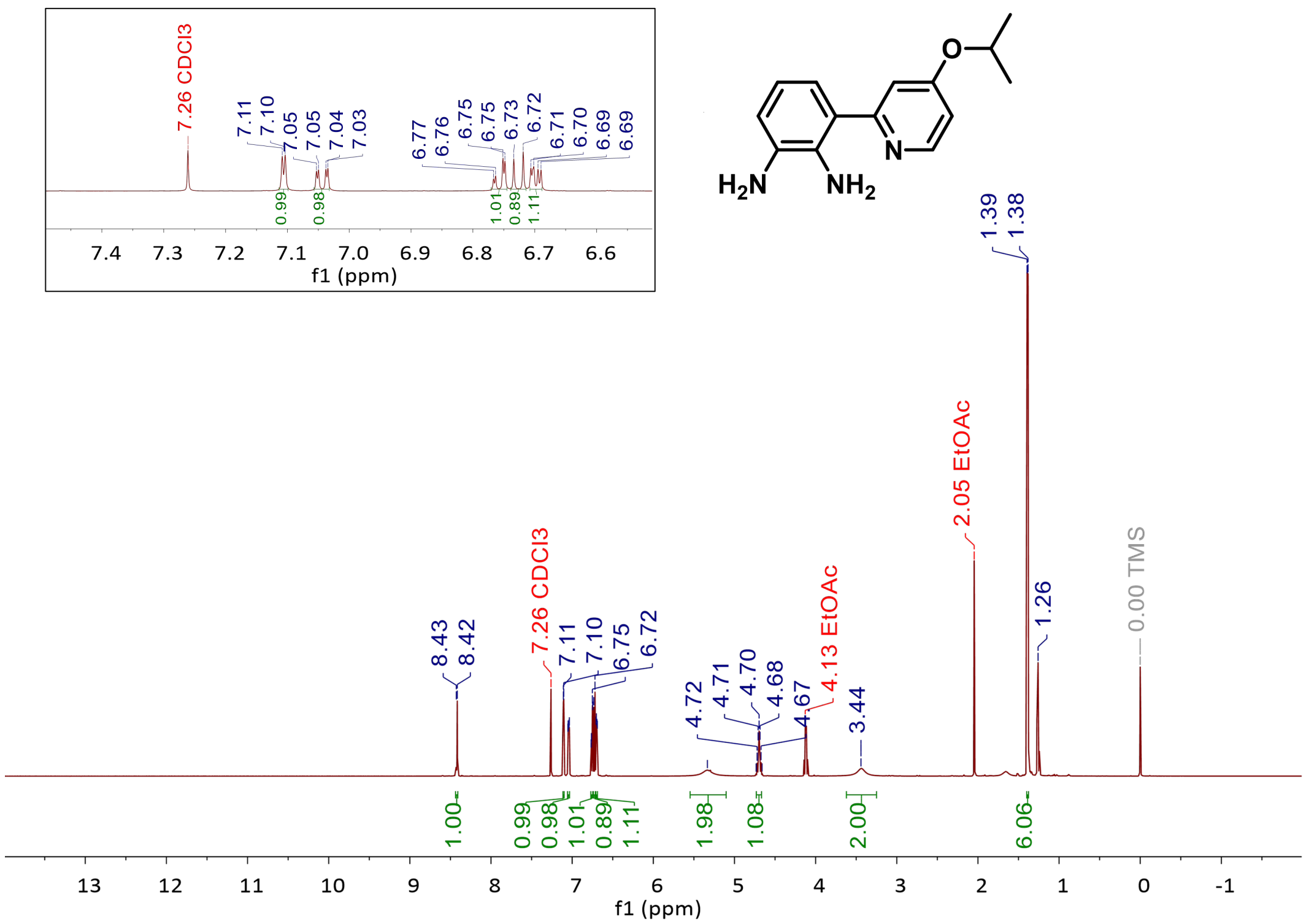

Fig. S58. ^{1}H NMR (500 MHz) spectrum of **4c** in $CDCl_3$ (T = 298 K).

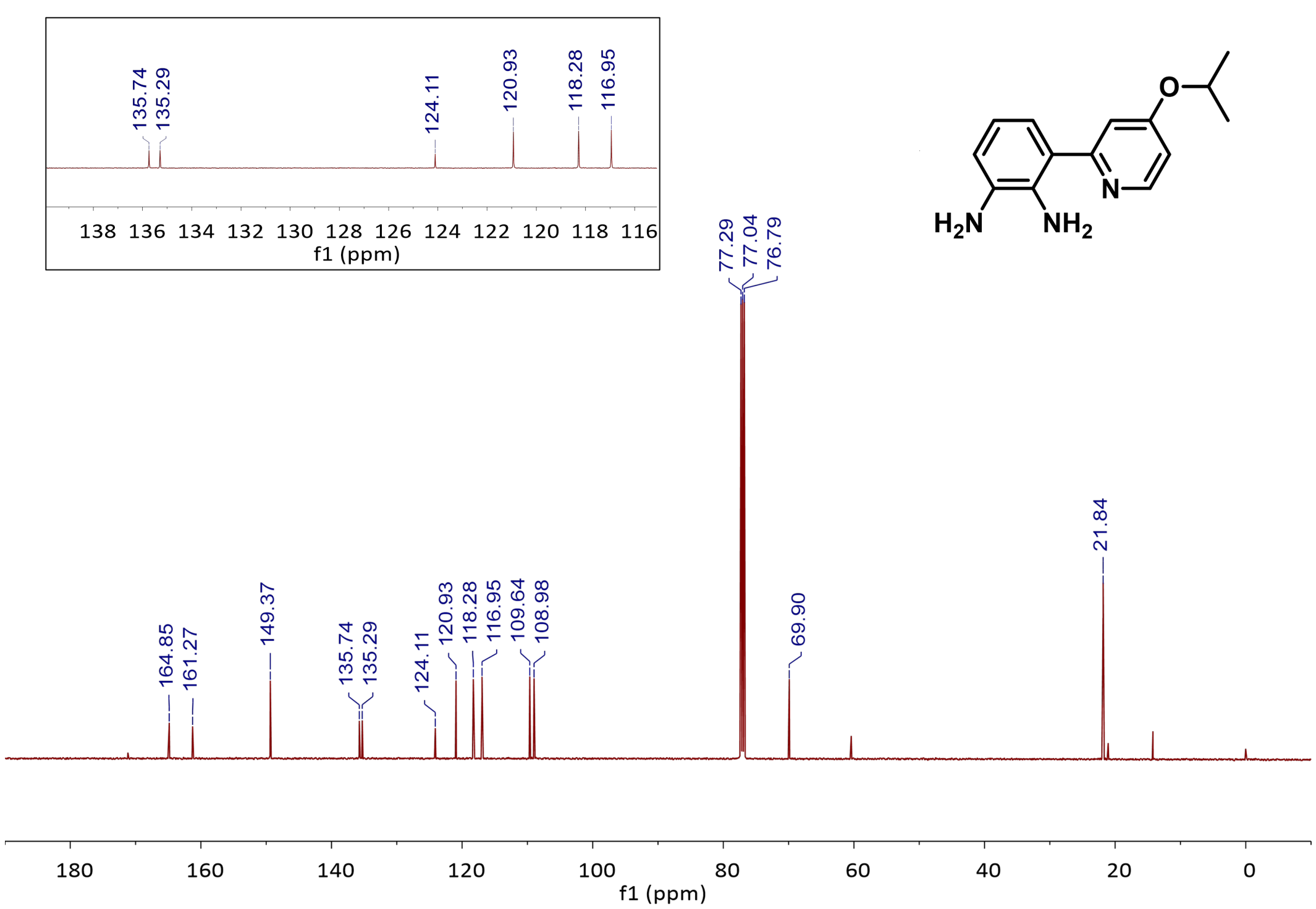

Fig. S59. ^{13}C NMR (125 MHz) spectrum of **4c** in $CDCl_3$ (T = 298 K).

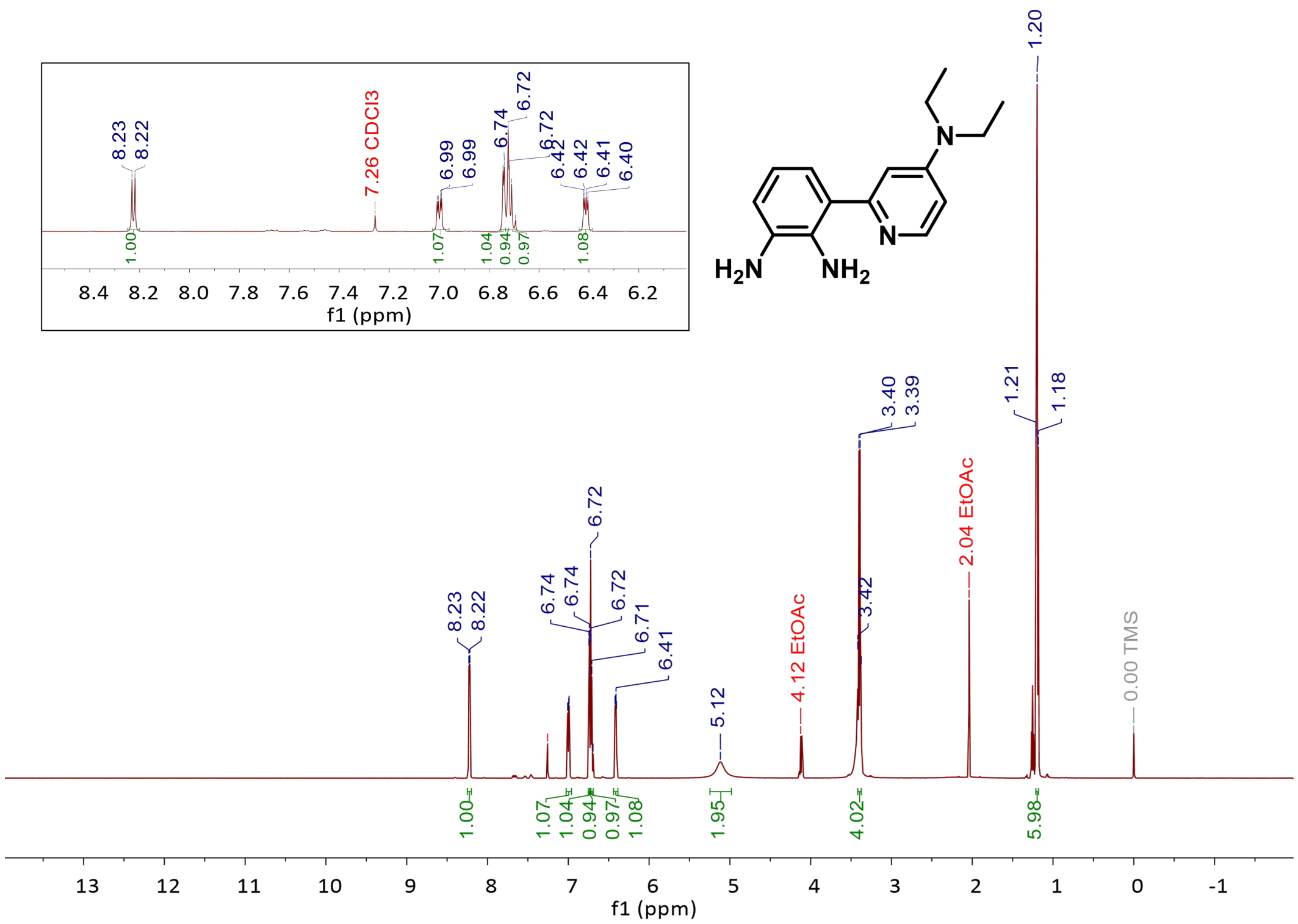

Fig. S60. ^{1}H NMR (500 MHz) spectrum of **4d** in $CDCl_3$ (T = 298 K).

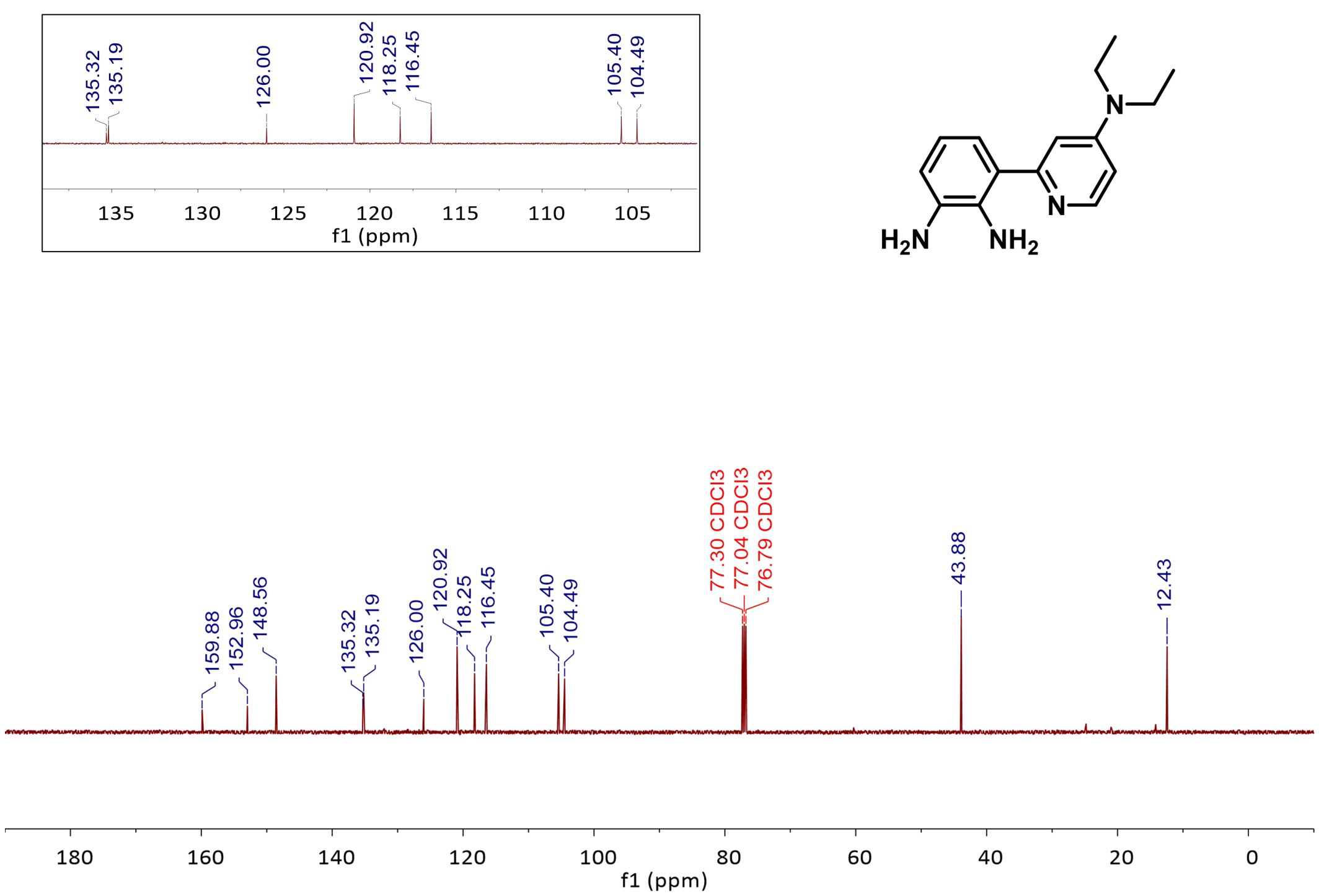

Fig. S61. ^{13}C NMR (125 MHz) spectrum of **4d** in $CDCl_3$ (T = 298 K).

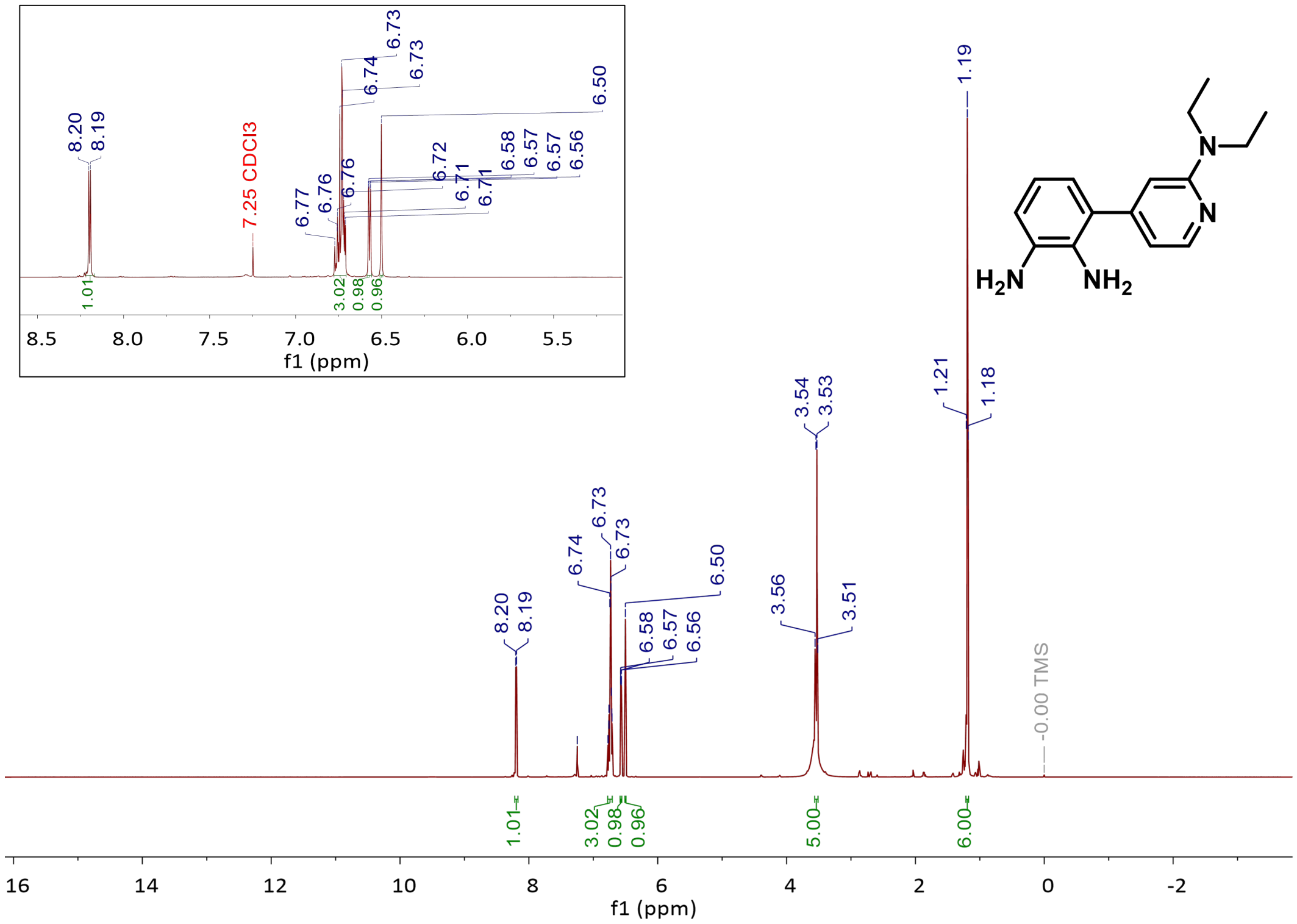

Fig. S62. ^{1}H NMR (500 MHz) spectrum of **4e** in $CDCl_3$ (T = 298 K).

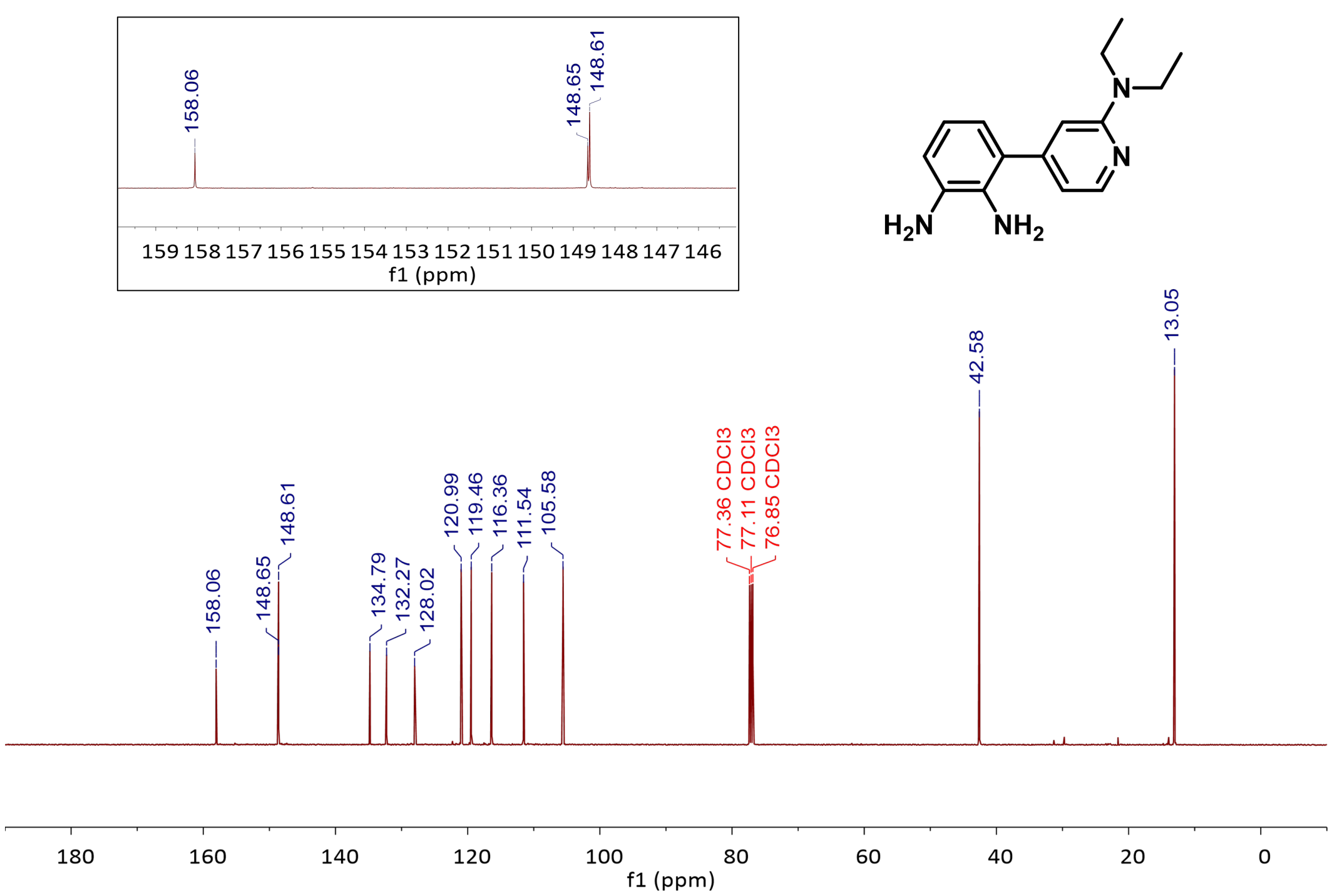

Fig. S63. ^{13}C NMR (125 MHz) spectrum of **4e** in $CDCl_3$ (T = 298 K).

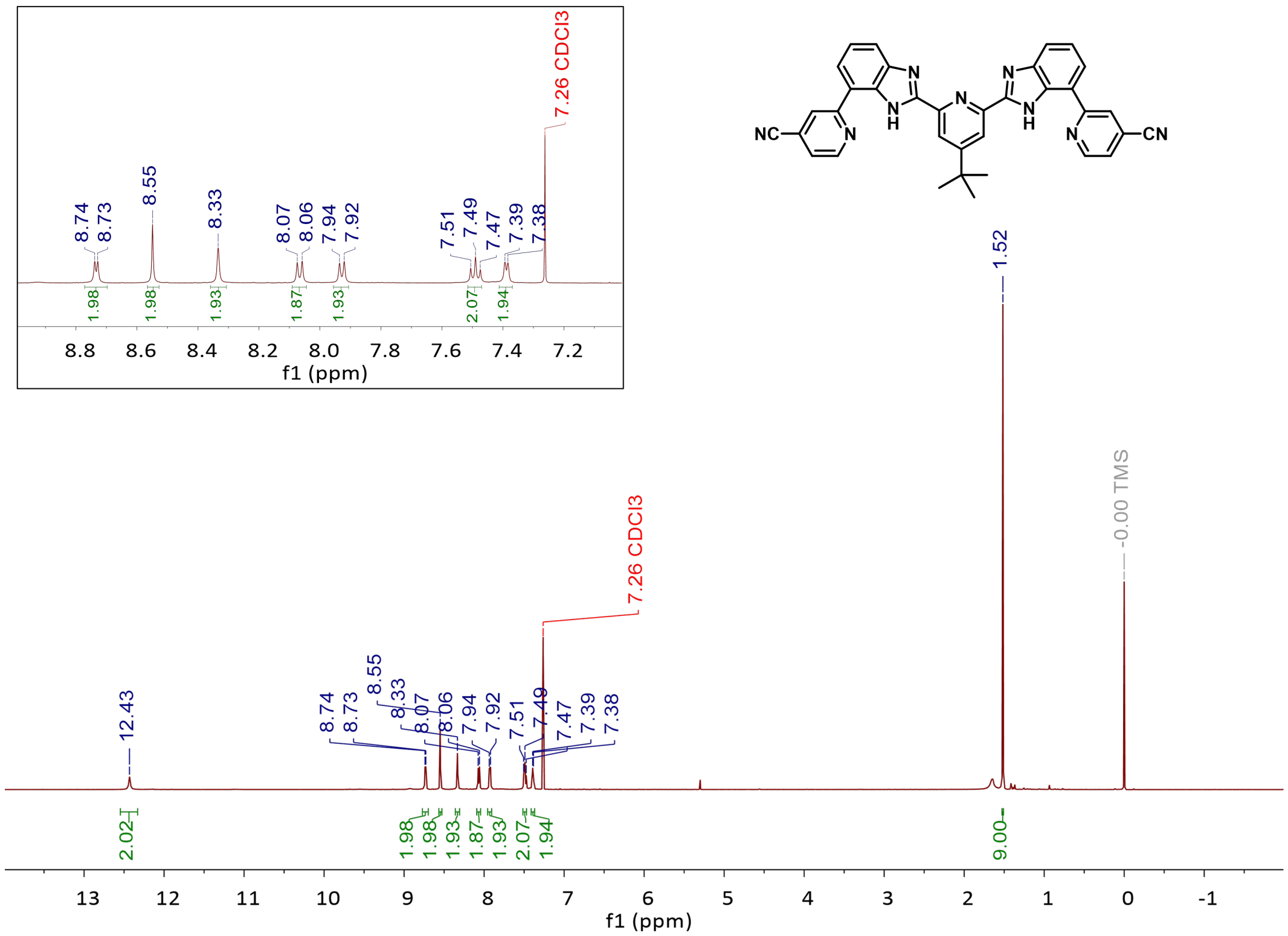

Fig. S64. ^{1}H NMR (500 MHz) spectrum of **5a** in $CDCl_3$ (T = 298 K).

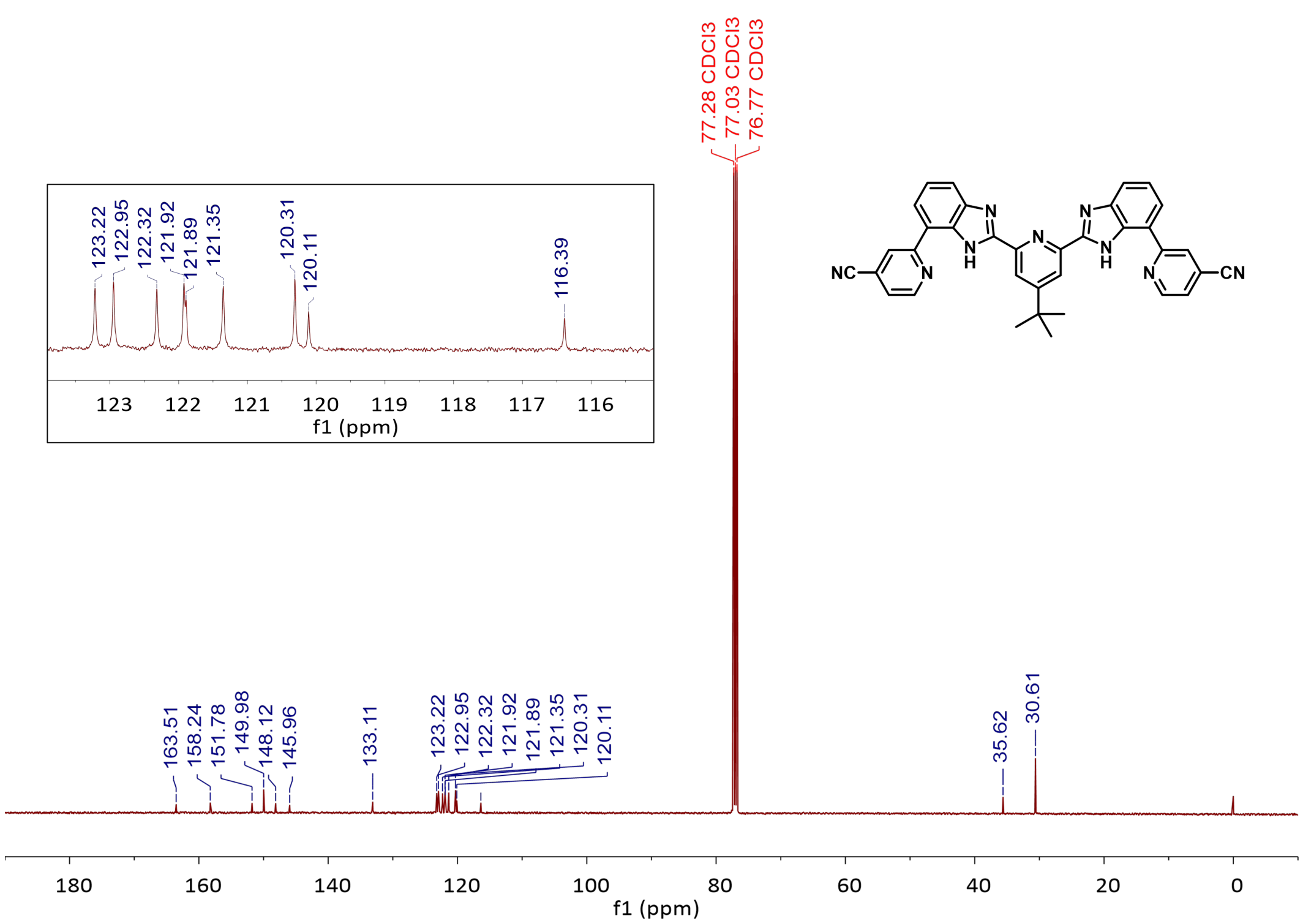

Fig. S65. ^{13}C NMR (125 MHz) spectrum of **5a** in $CDCl_3$ (T = 298 K).

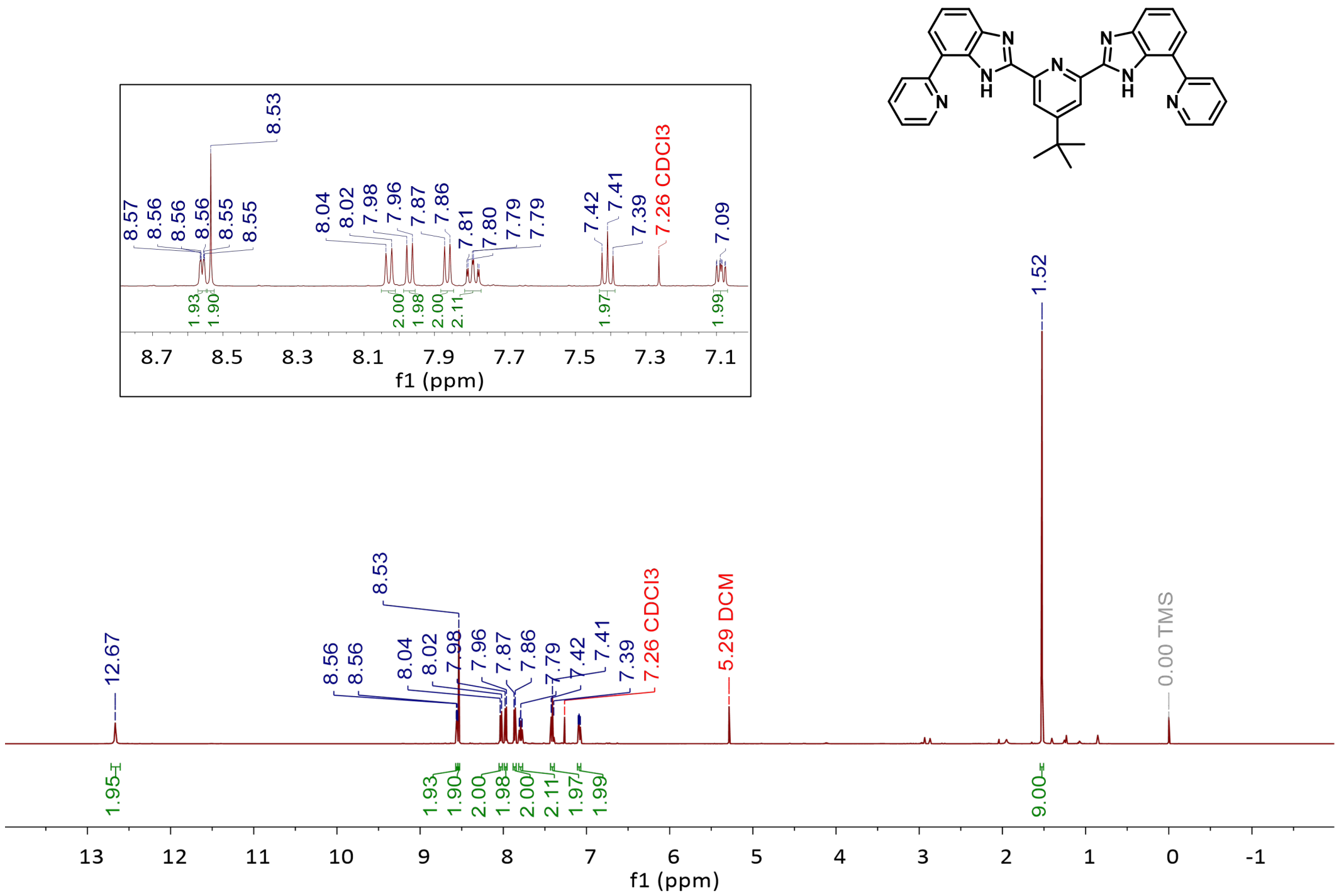

Fig. S66. ^{1}H NMR (500 MHz) spectrum of **5b** in $CDCl_3$ (T = 298 K).

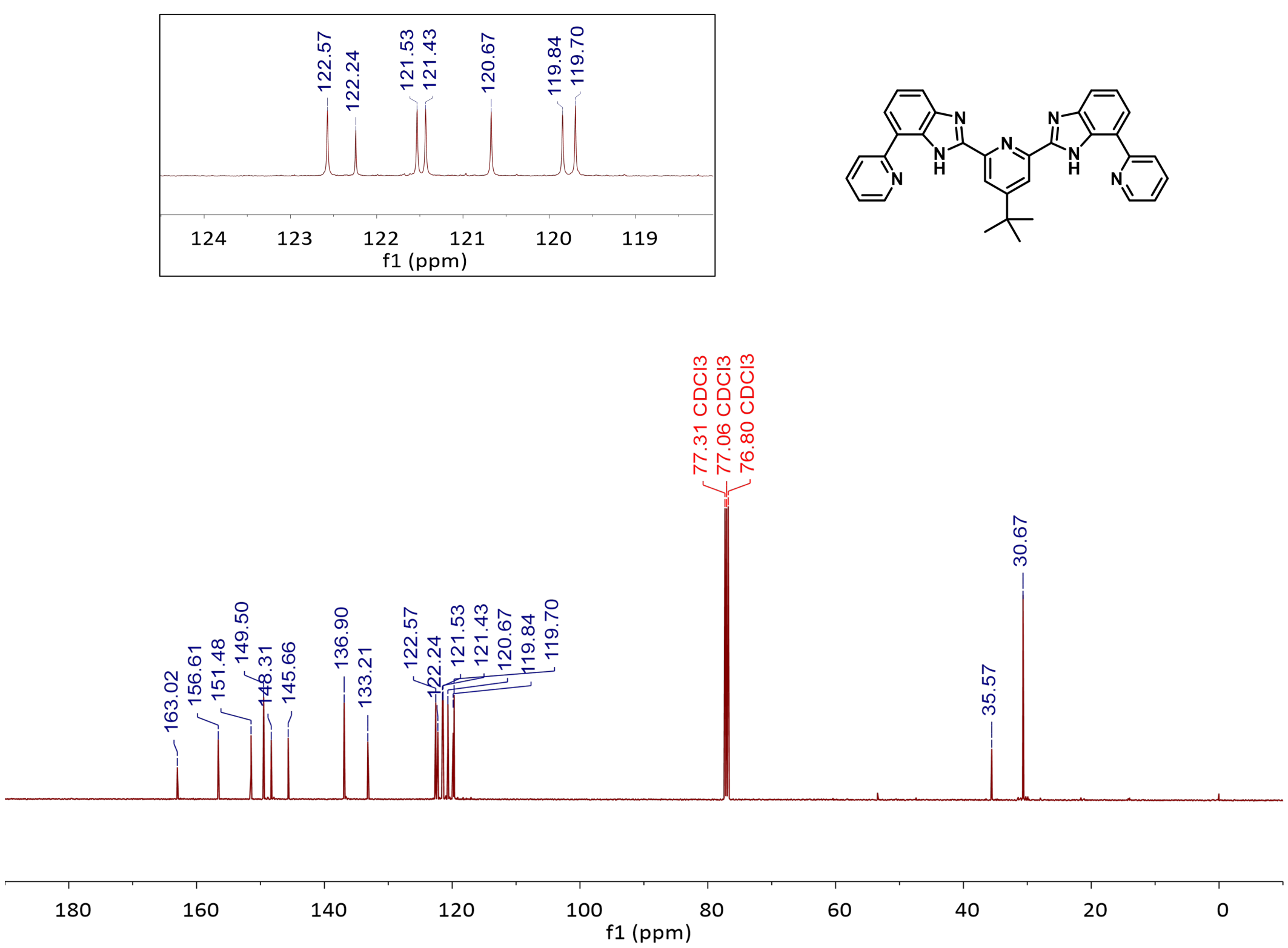

Fig. S67. ^{13}C NMR (125 MHz) spectrum of **5b** in $CDCl_3$ (T = 298 K).

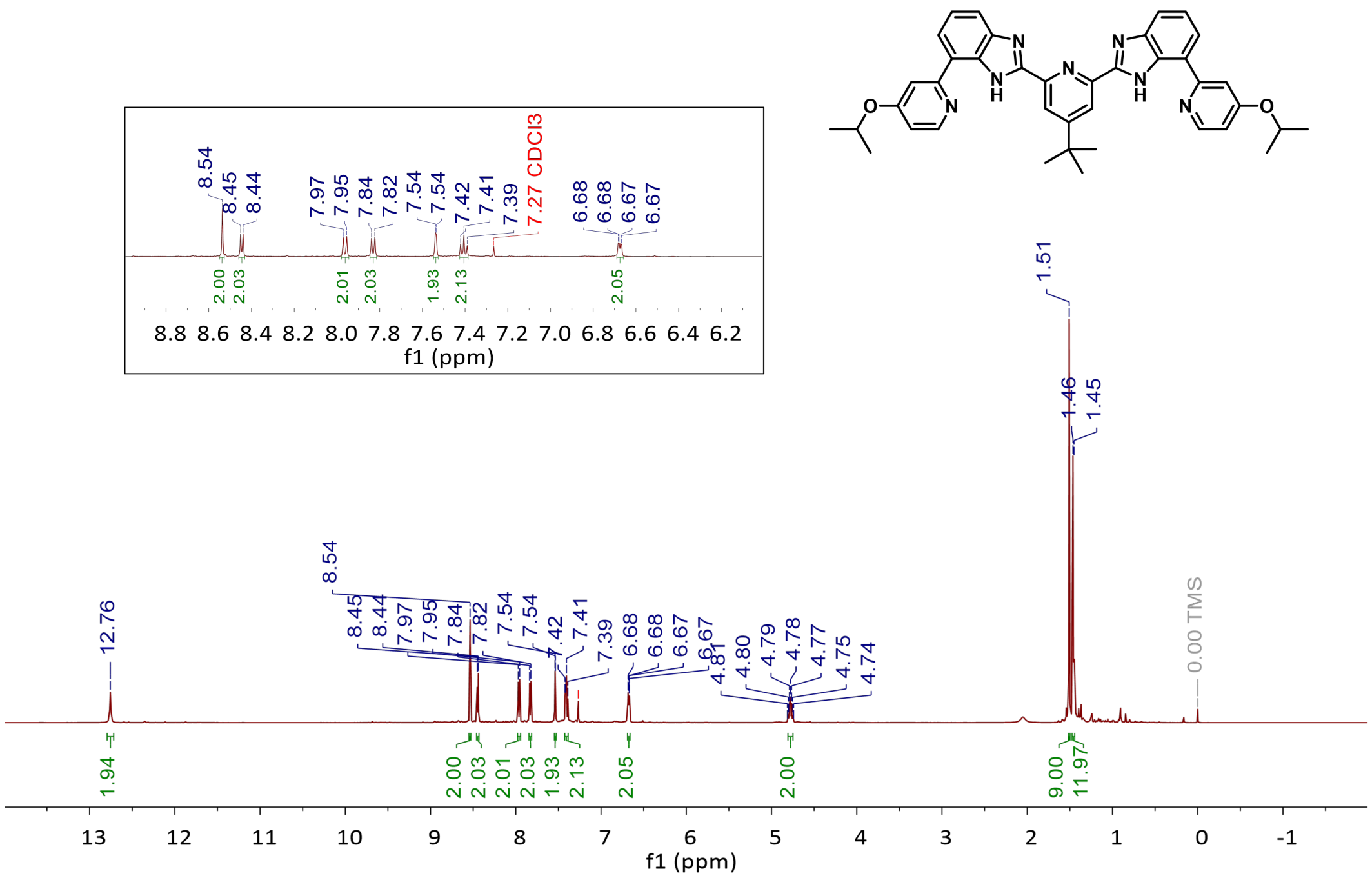

Fig. S68. ^{1}H NMR (500 MHz) spectrum of **5c** in $CDCl_3$ (T = 298 K).

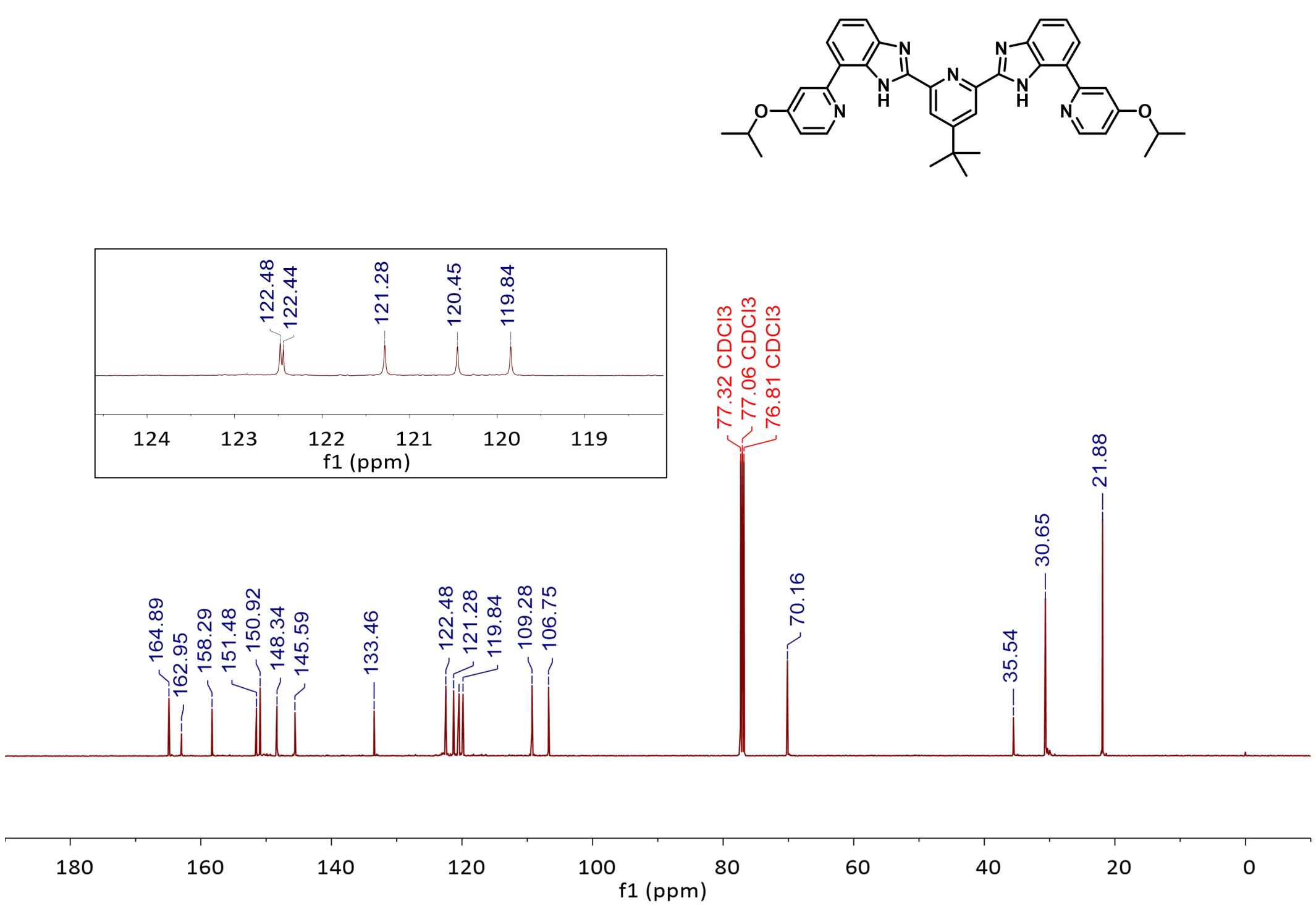

Fig. S69. ^{13}C NMR (125 MHz) spectrum of **5c** in $CDCl_3$ (T = 298 K).

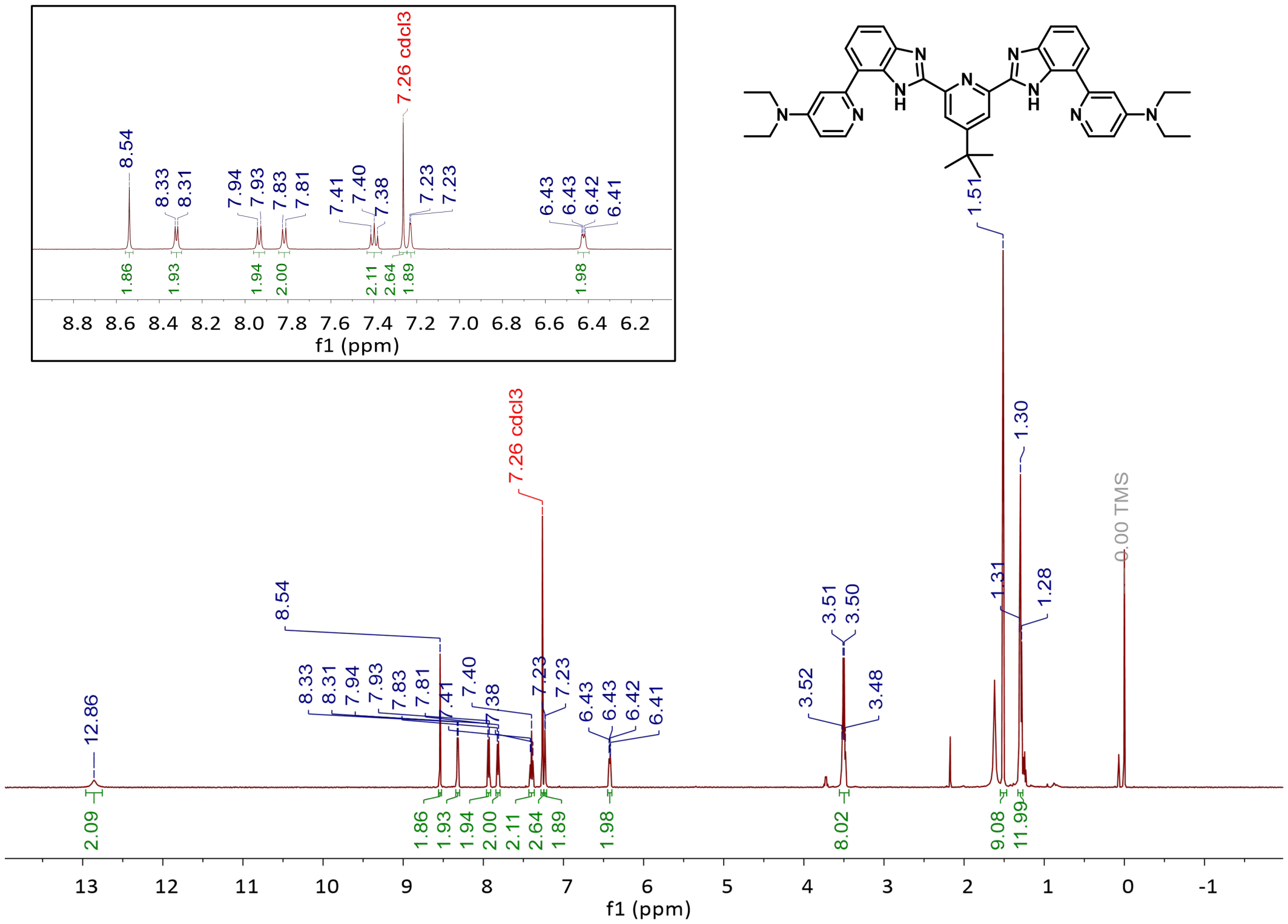

Fig. S70. ^{1}H NMR (500 MHz) spectrum of **5d** in $CDCl_3$ (T = 298 K).

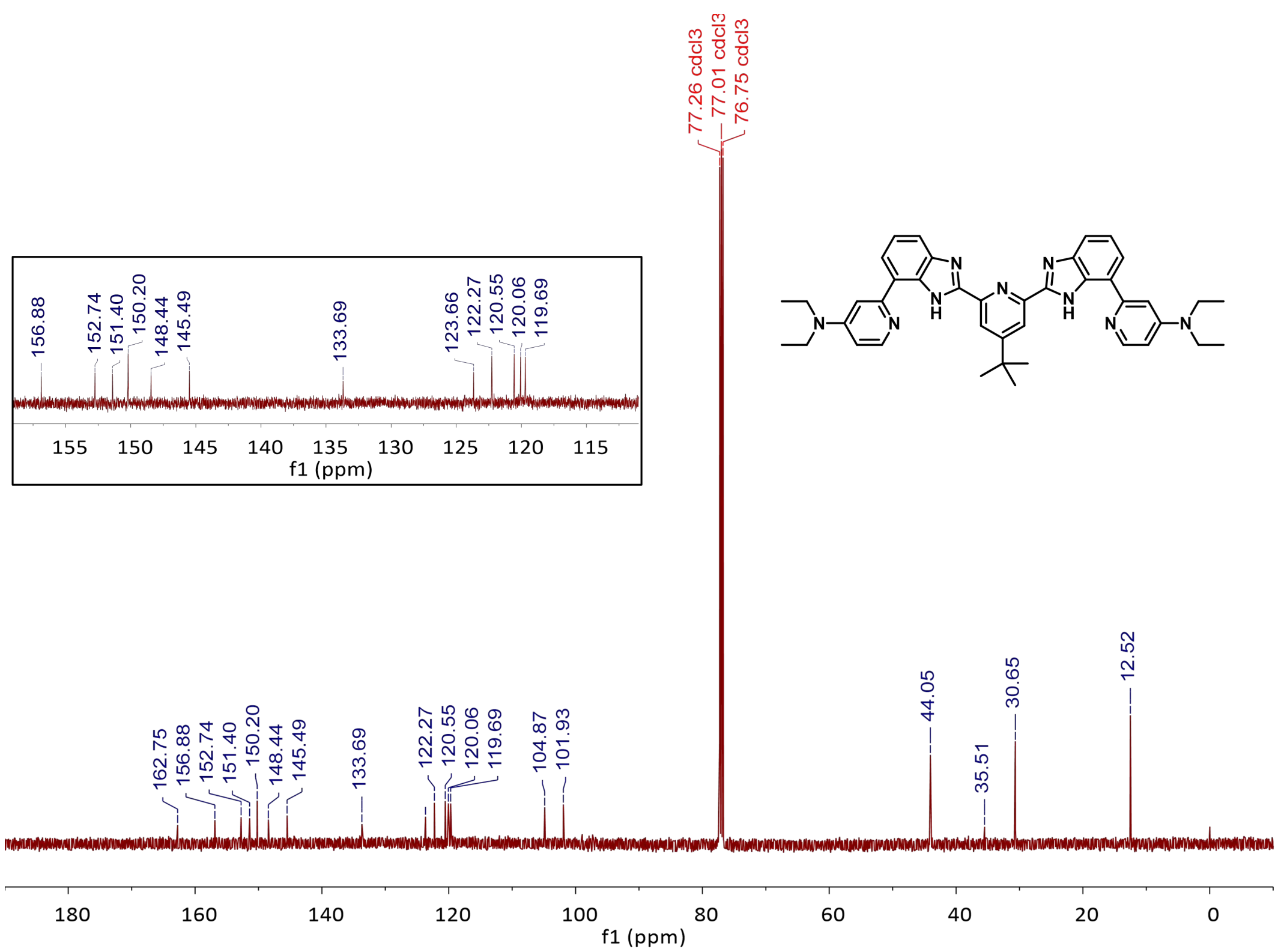

Fig. S71. ^{13}C NMR (125 MHz) spectrum of **5d** in $CDCl_3$ (T = 298 K).

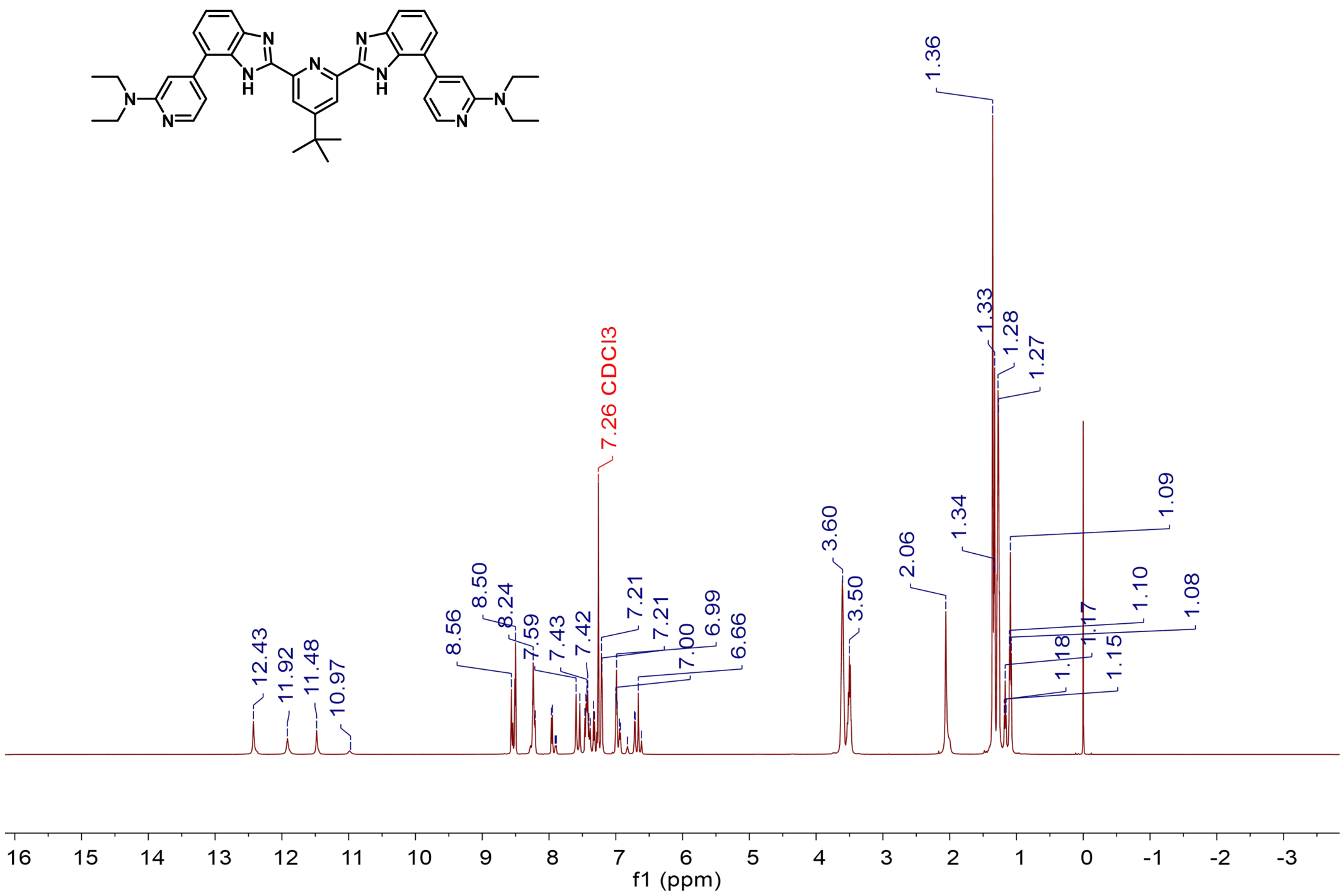

Fig. S72. ^{1}H NMR (500 MHz) spectrum of **5e** in $CDCl_3$ (T = 298 K). Four distinct benzimidazole N–H resonances are observed for **5e** with substantially upfield shifts (δ = 10.98–12.43 ppm), suggesting weaker and poorly organized hydrogen bonding. Deuterium exchange at the basic N–H sites (in a 99:1 (v/v) mixture of CD_3OD and TFA-d_1) or the use of HB-accepting solvent (THF-d_8) restore the simple ^{1}H and ^{13}C NMR spectral pattern anticipated from the chemical structure having mirror symmetry (See below for Figs. S60–S62).

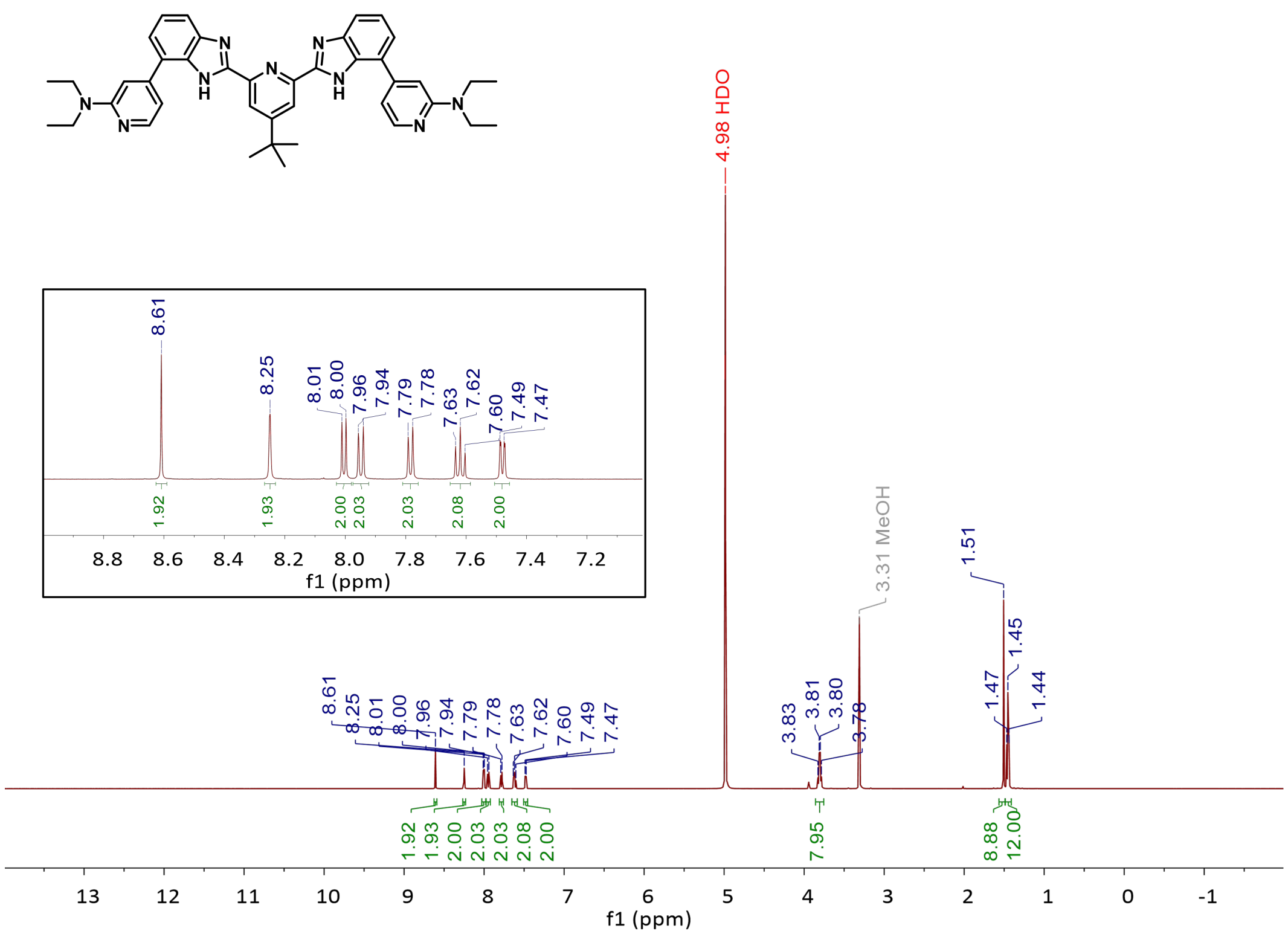

Fig. S73. ^{1}H NMR (500 MHz) spectrum of **5e** in 1% TFA-d_1 in CD_3OD (T = 298 K).

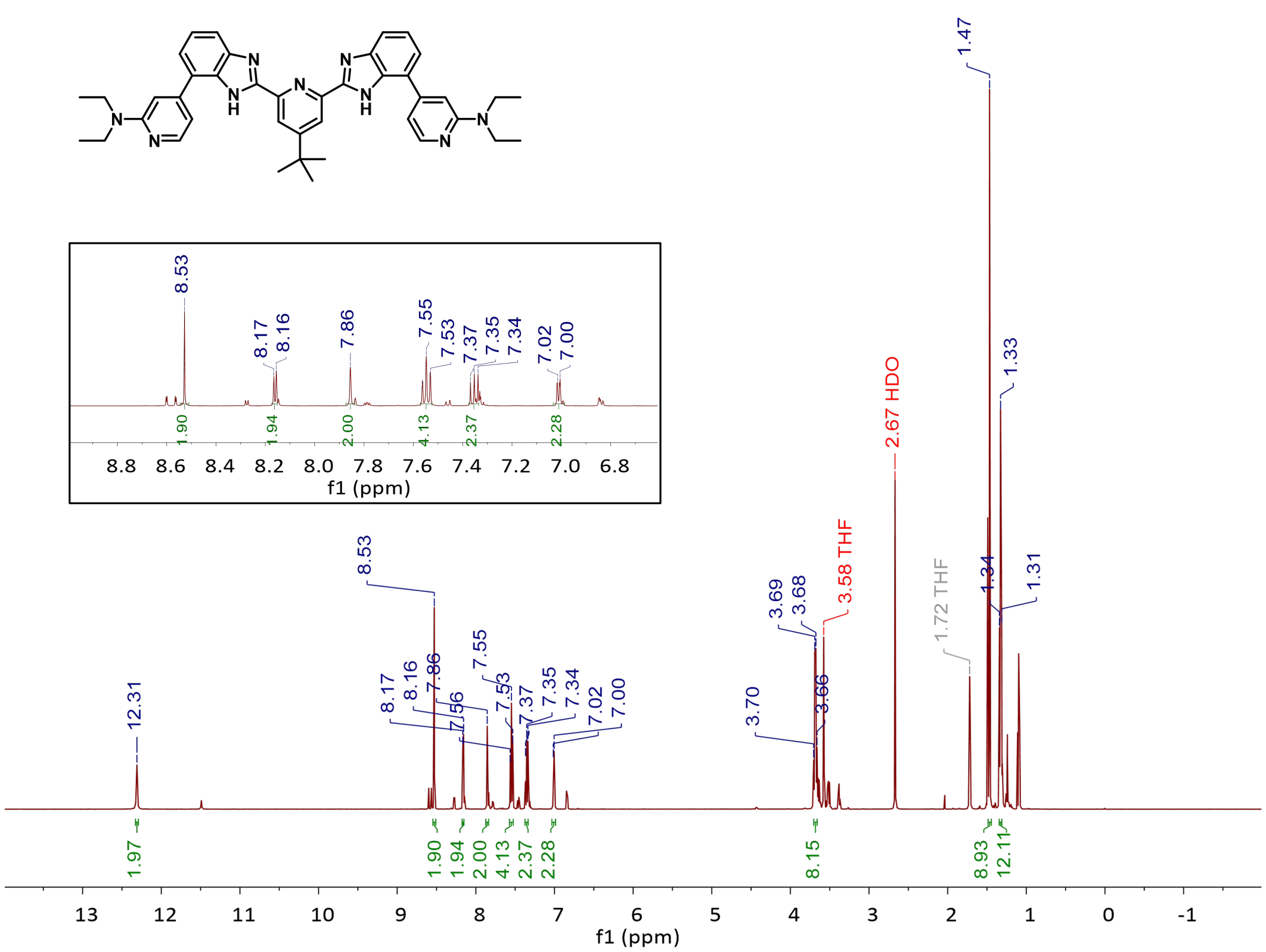

Fig. S74. ^{1}H NMR (500 MHz) spectrum of **5e** in THF-d_8 (T = 298 K).

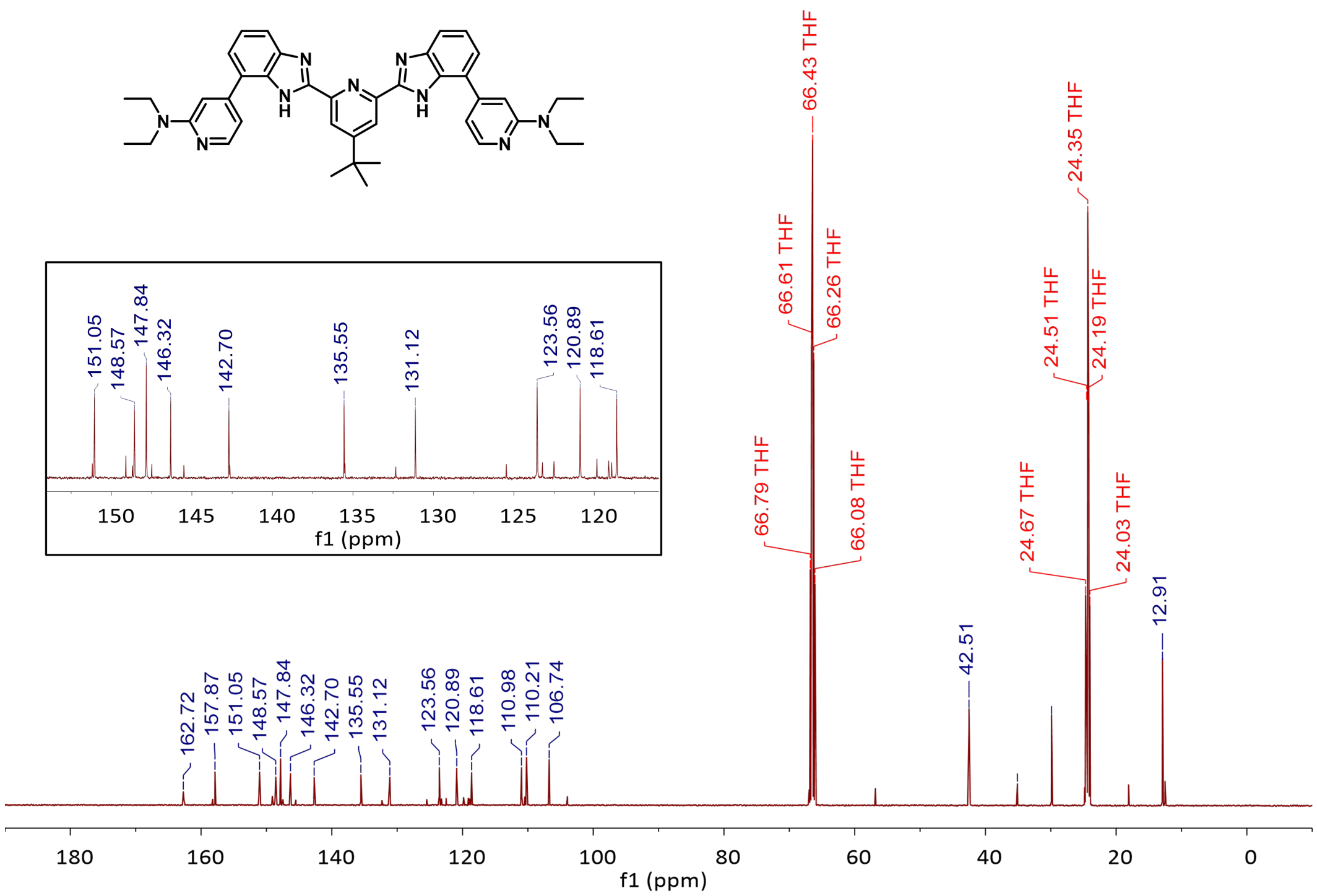

Fig. S75. ^{13}C NMR (125 MHz) spectrum of **5e** in THF-d_8 (T = 298 K).

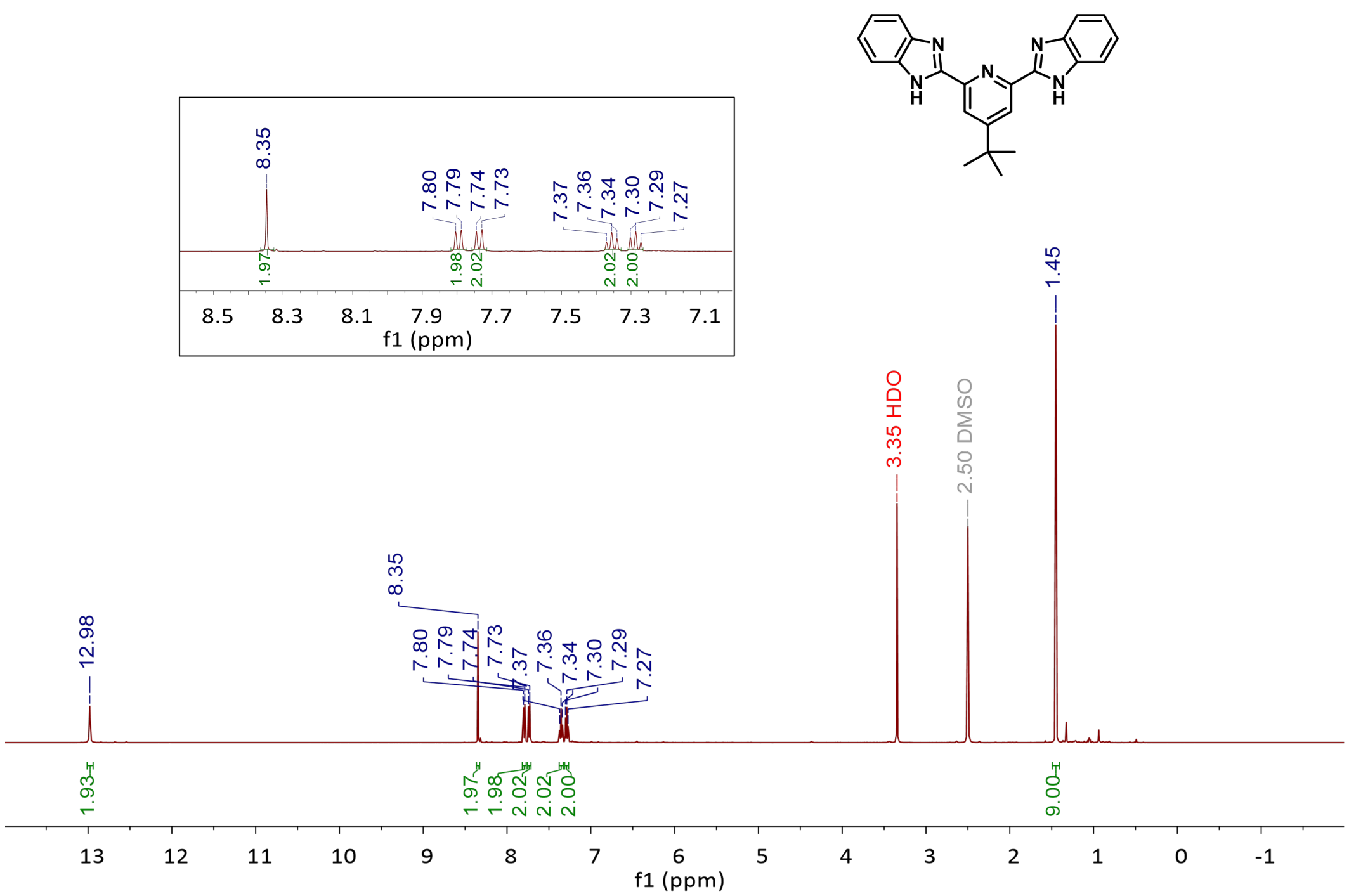

Fig. S76. ^{1}H NMR (500 MHz) spectrum of **5f** in DMSO-d_6 (T = 298 K).

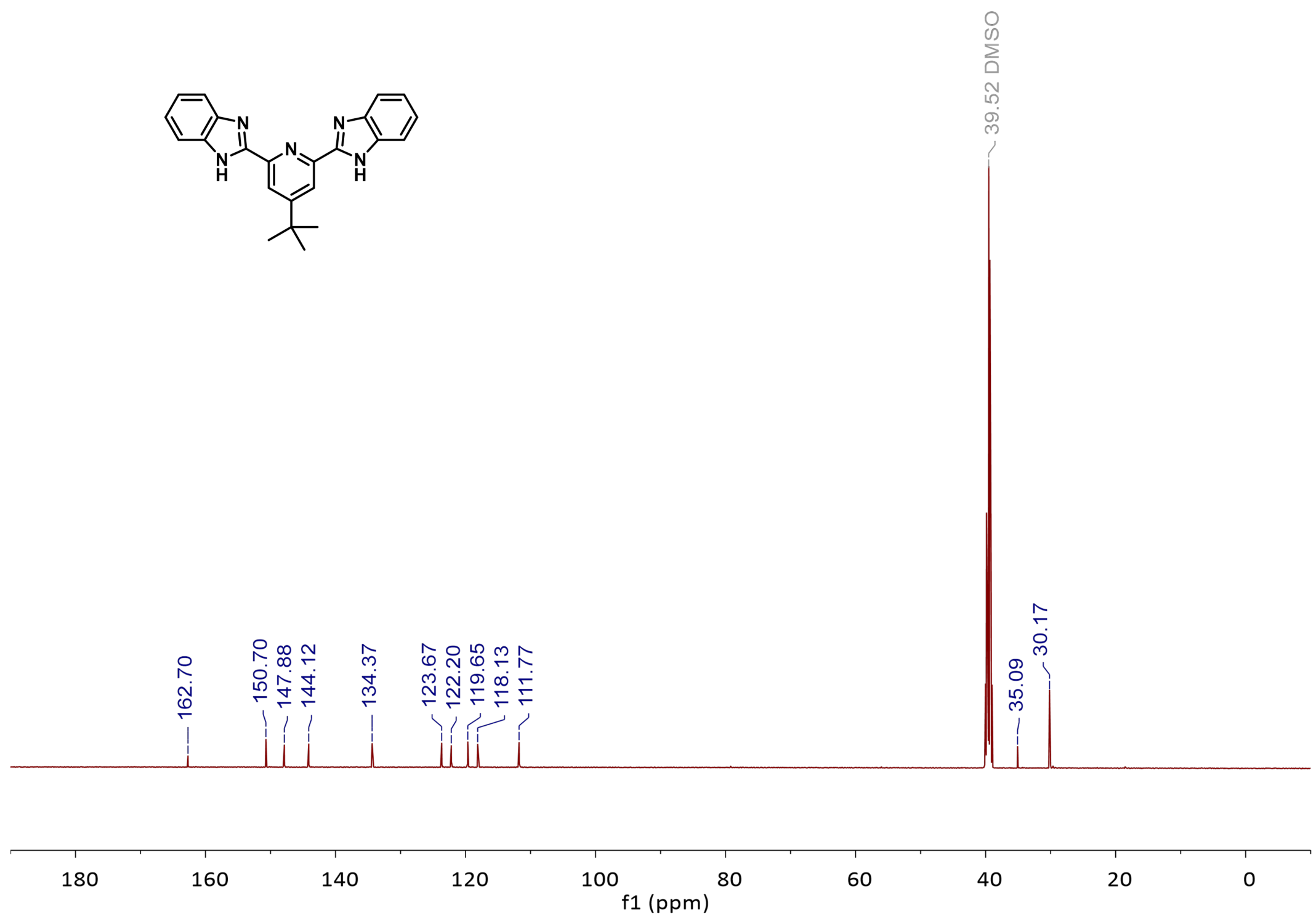

Fig. S77. ^{13}C NMR (500 MHz) spectrum of **5f** in DMSO-d_6 (T = 298 K).
